# The earliest lead ore processing in Europe. 5^th^ millennium BC finds from Pietrele on the Lower Danube

**DOI:** 10.1371/journal.pone.0214218

**Published:** 2019-04-10

**Authors:** Svend Hansen, Ignacio Montero-Ruiz, Salvador Rovira, Daniel Steiniger, Meda Toderaş

**Affiliations:** 1 Eurasien-Abteilung des Deutschen Archäologischen Instituts, Berlin, Germany; 2 Centro de Ciencias Humanas y Sociales, Madrid, Spain; 3 Museo Arqueológico Nacional - Department of Conservation, Madrid, Spain; 4 Institutul de Arheologie "Vasile Pârvan" Academia Română, Bucharest, Romania; University at Buffalo - The State University of New York, UNITED STATES

## Abstract

Eleven biconical vessels from the Copper Age sites Pietrele and Blejeşti (Romania) have been investigated using p-XRF. In most cases, traces of lead could be measured on their surfaces. Samples of slag-like material from two vessels and the clay of one vessel were investigated using laboratory methods, namely SEM, XRD, LIA and optical microscopy. The vessels were obviously used as a kind of crucible in which slag-like remains and galena ore were detected. It still remains unclear as to what final product was gained by smelting galena in this way. The amount of these such vessels in the Pietrele settlement, their appearance as grave goods in Pietrele and Vărăști (Romania), and their supposed occurrence in a number of other Copper Age settlements in Romania and Bulgaria show the significance of this phenomenon. It must have been a widespread and more or less well known practice, an important part of cultural habit during a particular period in the Lower Danube region and likely even farther afield. For the first time, extensive experimentation with lead ore can be shown in a clear chronological horizon, ca. 4400–4300 BCE in southeastern Europe.

## Introduction and archaeological context

Metallurgy is one of the basic innovations that changed the lives of prehistoric societies and still plays a decisive role in modern societies; it is worth highlighting here the enormous importance of the Rare Earth Elements for industrial purposes.

**Fig 1:**
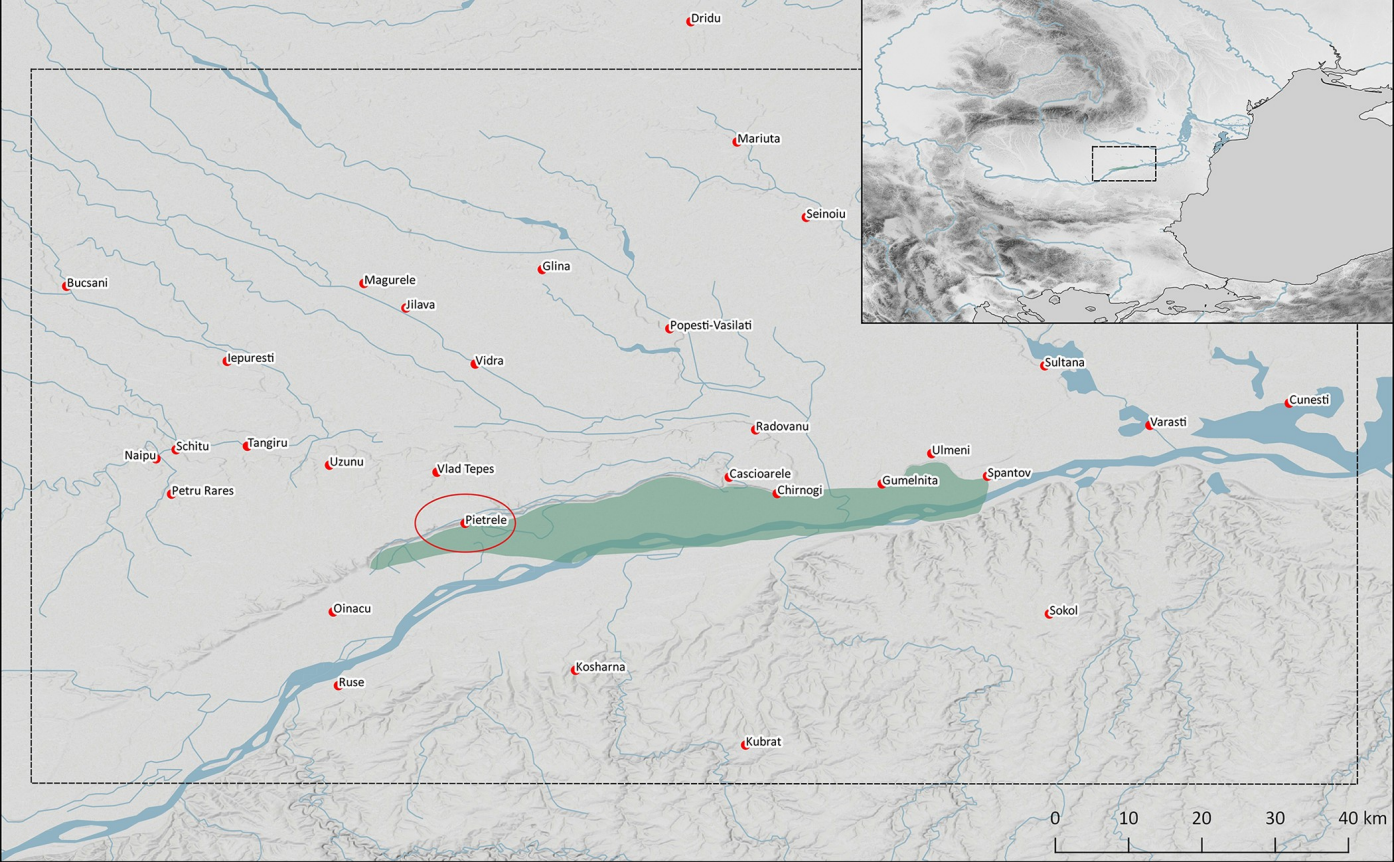
Map of Copper Age sites in the Lower Danube area and Bulgaria mentioned in the text (Map M. Karaucak).

According to the present state of research, copper mining, processing and casting started at the turn of the 6^th^ to the 5^th^ millennium BCE [1]. The Balkan peninsula (Fig 1) played an important role at that time, but archaeological finds from the Levant (Tel Tsaf) [2] and the Iranian plateau (Tal-e Eblis) [3] also provide evidence for a comparable early metallurgy. The significant amount of metal objects and the meticulous studies conducted in Europe suggest the origin of metallurgy to have been located in the Balkans [4]. In fact, it is impossible to identify one sole region as the origin of copper casting; instead, the rapid dissemination of metallurgical knowledge seems to be one of the characteristic properties of early metallurgy [5]. The reasons for this are manifold; one factor could, for instance, have been the ongoing search for new raw material sources. The oft-mentioned special status of the metallurgist might also have played a further role and, finally, the far-reaching transfer of metallurgical knowledge was the condition and the reason for the preservation and development of this knowledge. As early as the 5^th^ millennium BCE, metallurgical activities can be attested in the Alps [6] and on the Iberian Peninsula [7].

Nevertheless, the Balkan Peninsula offers the largest inventory of copper artefacts from the 5^th^ millennium BCE. Alongside copper pins and bracelets, awls played an important role as universal tools. Larger and heavier implements included adzes and axes that could be used as tools and weapons. Gold objects like bracelets, discs and sceptres furthermore became components of the representation of power, especially in graves. The cemetery of Varna on the Black Sea Coast shows blatant social differentiation, allowing us to speak rightly of one of the origins of social inequality [8]. New radiocarbon dates have pushed back the beginning of the Varna cemetery into the 46^th^ century BCE [9].

**Fig 2:**
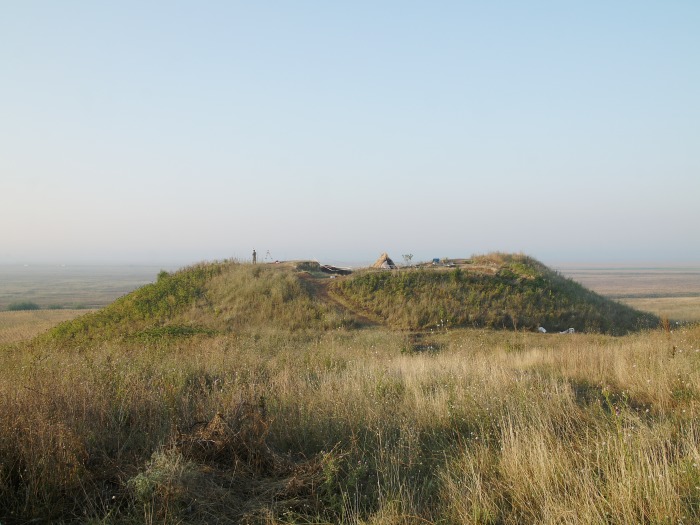
View of the settlement mound from the North (Photo S. Hansen).

Metal objects played an enormous role in settlements as well. In the Copper Age settlement mound of Măgura Gorgana (Figs 1 and 2) near Pietrele, jud. Giurgiu in the Wallachian plain of Romania, more than 250 copper and two gold objects have been recorded since 2004 [10]. Excavations there have uncovered a continuous sequence of seven settlement phases dated between 4550 and 4300 BCE. The oldest phase of the settlement mound has not yet been reached, but it can be estimated to date to around 4600 BCE. The thus far longest radiocarbon sequence in a Copper Age settlement in southern Romania offers insight into the economic development over ca. 300 years’ time. There is clear evidence for specialisation within households. In Trench F, a sequence of seven houses could be excavated that are thought to be the homes of hunters and fishermen. These clearly represent the early beginnings of professions, also evident in the production of flint blades and pottery.

Traces of copper and gold metallurgy are absent in Pietrele, the objects having likely been brought from elsewhere. Four different mines have been identified as the origin of the copper items in Pietrele [11]. Mining activities could be identified already in 1970, when Evgeny Cernych started his investigations in Ai Bunar, Bulgaria [12]. Whereas other mines have been identified in the meantime, relics of copper casting are still lacking across the entire Balkan Peninsula. Hence, the casting method of the heavy metal axes is still a point of discussion.

**Fig 3:**
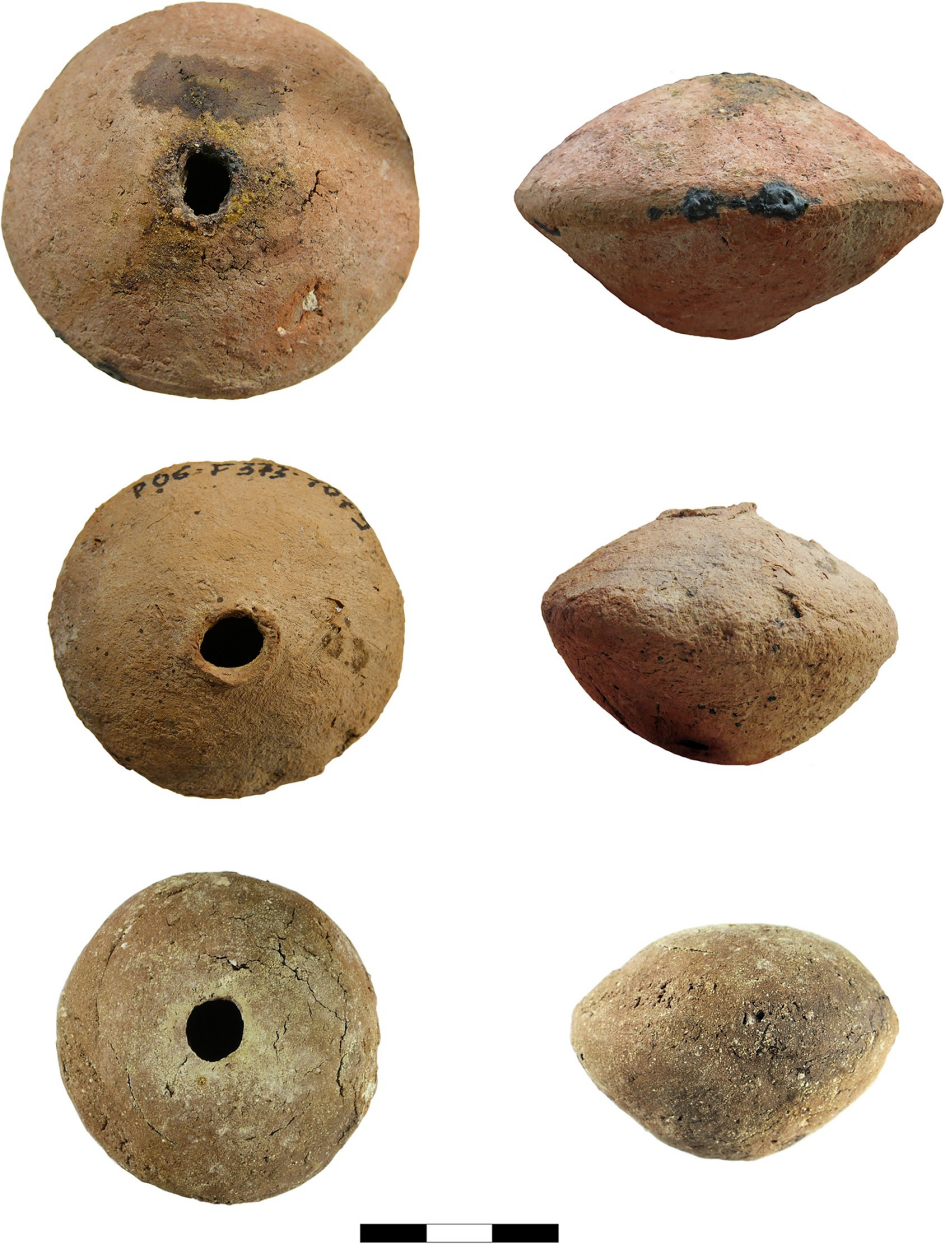
P06F3741096, P06F3731079, P13F7230116 (Photos D. Steiniger).

**Fig 4:**
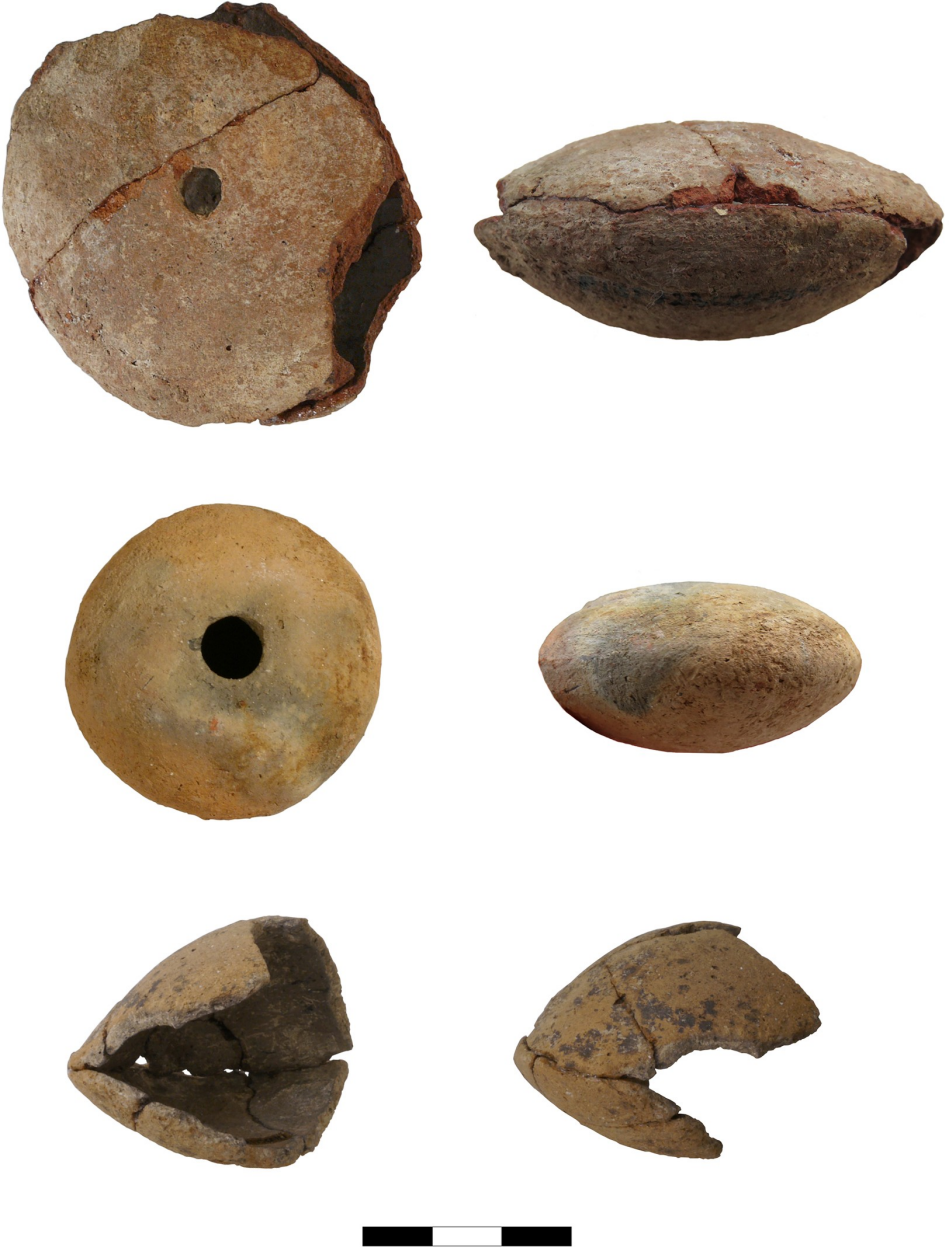
10 P13F723CER53.4, P06B2360140, P12R1190577 (Photos S. Hansen, D. Steiniger).

**Fig 5:**
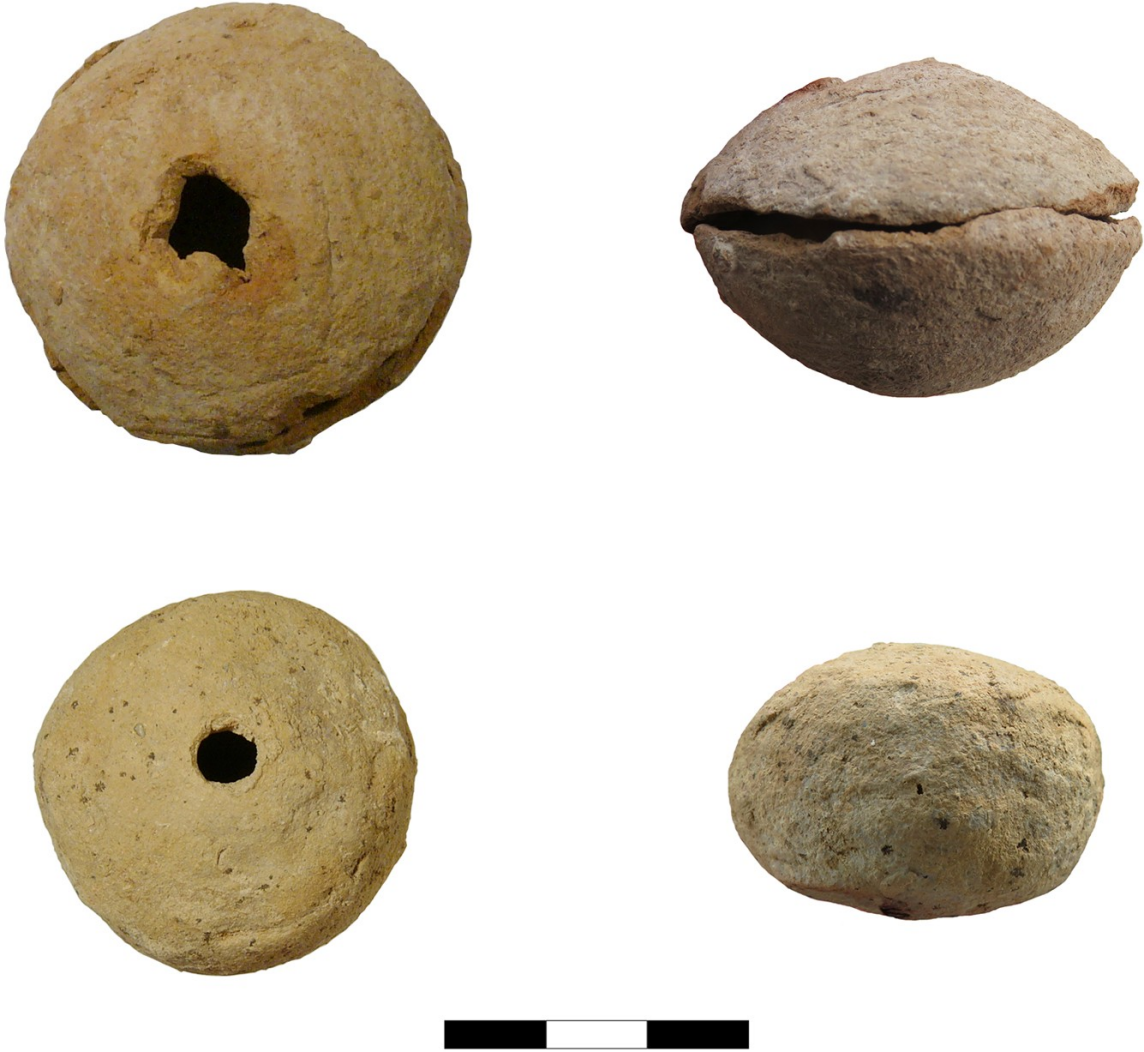
P05B1220055, P06F3610857 (Photos D. Steiniger).

**Fig 6:**
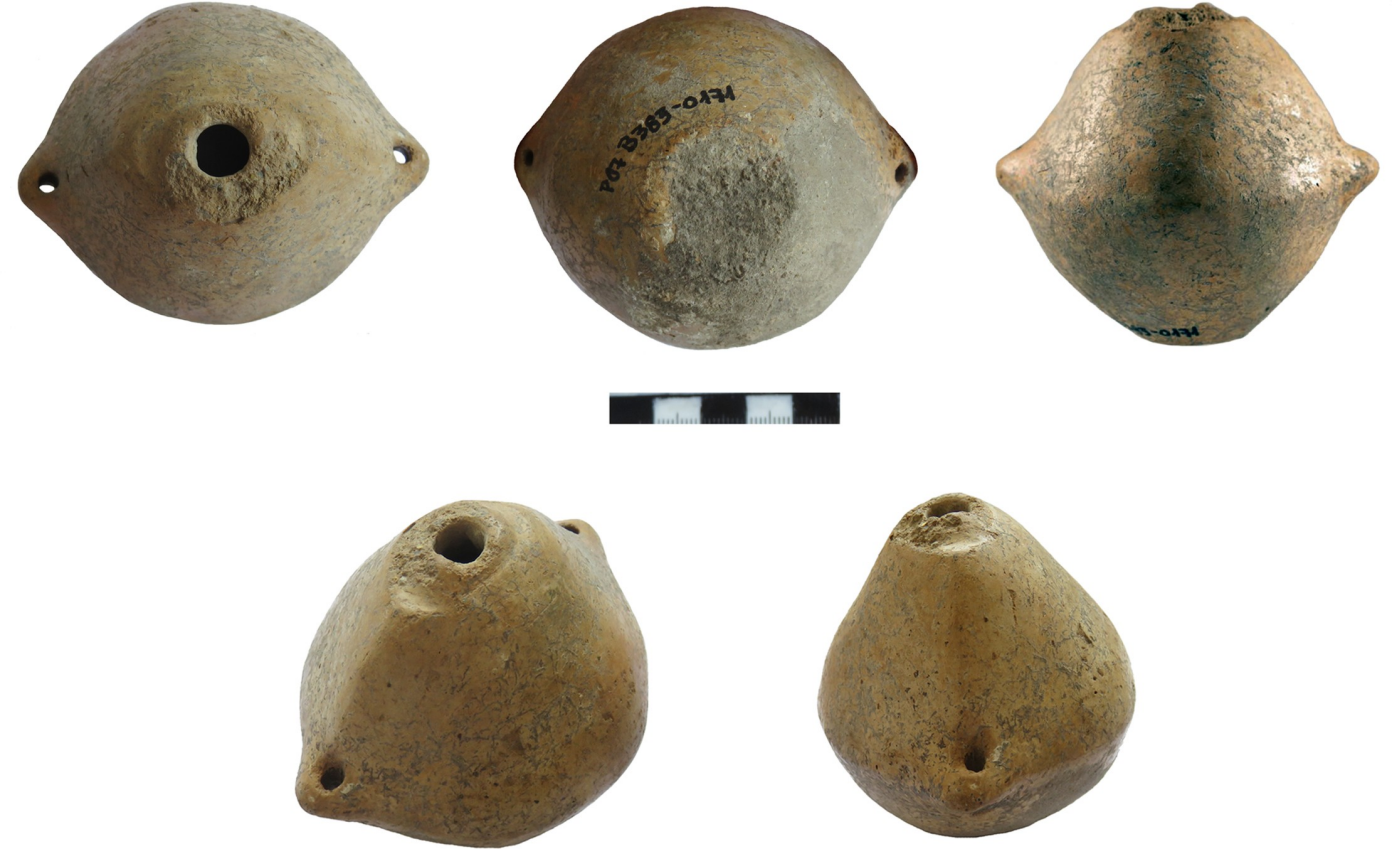
P07B3830171 (Photos S. Hansen, D. Steiniger).

During the 2016 field campaign in Pietrele, we were able to examine several small vessels by means of a portable XRF device, whereupon traces of lead were identified on most of these vessels (Figs 3–6). With one exception (P07B3830171), all of these vessels are of a flat biconical shape. They were produced in two halves, which were dried until they had a leathery consistency, then joined together using water and some wet clay as an adhesive.

Traces of fire on the bottom side and melted clay and slag-like material in the small hole on the top as well as at the point where the two halves of the vessels were joined make clear that the small vessels were in fact crucibles. Before joining the two halves, the ore was probably placed in the vessel or it was filled in later through the small hole. Then it was ready to be set into the fire, where the metal could melt under relatively high temperatures without destroying the vessel, which was burnt in the fire as well. The molten material could ultimately be poured out through the hole in the top of the vessel, through which it could have also been possible to blow oxygen, in all probability with a reed used as a blowpipe.

Apart from the finds from recent excavations, two similar vessels had already been found in earlier excavations in Pietrele in 1943 and 1948. The slaggy material on one of the two containers with a diameter of 8 cm was then analysed and determined to be ‘lead carbonate’ (Pb 71.41% and CO_3_ 20.89%) [13]. Unfortunately, the location of these vessels is currently unknown.

**Fig 7:**
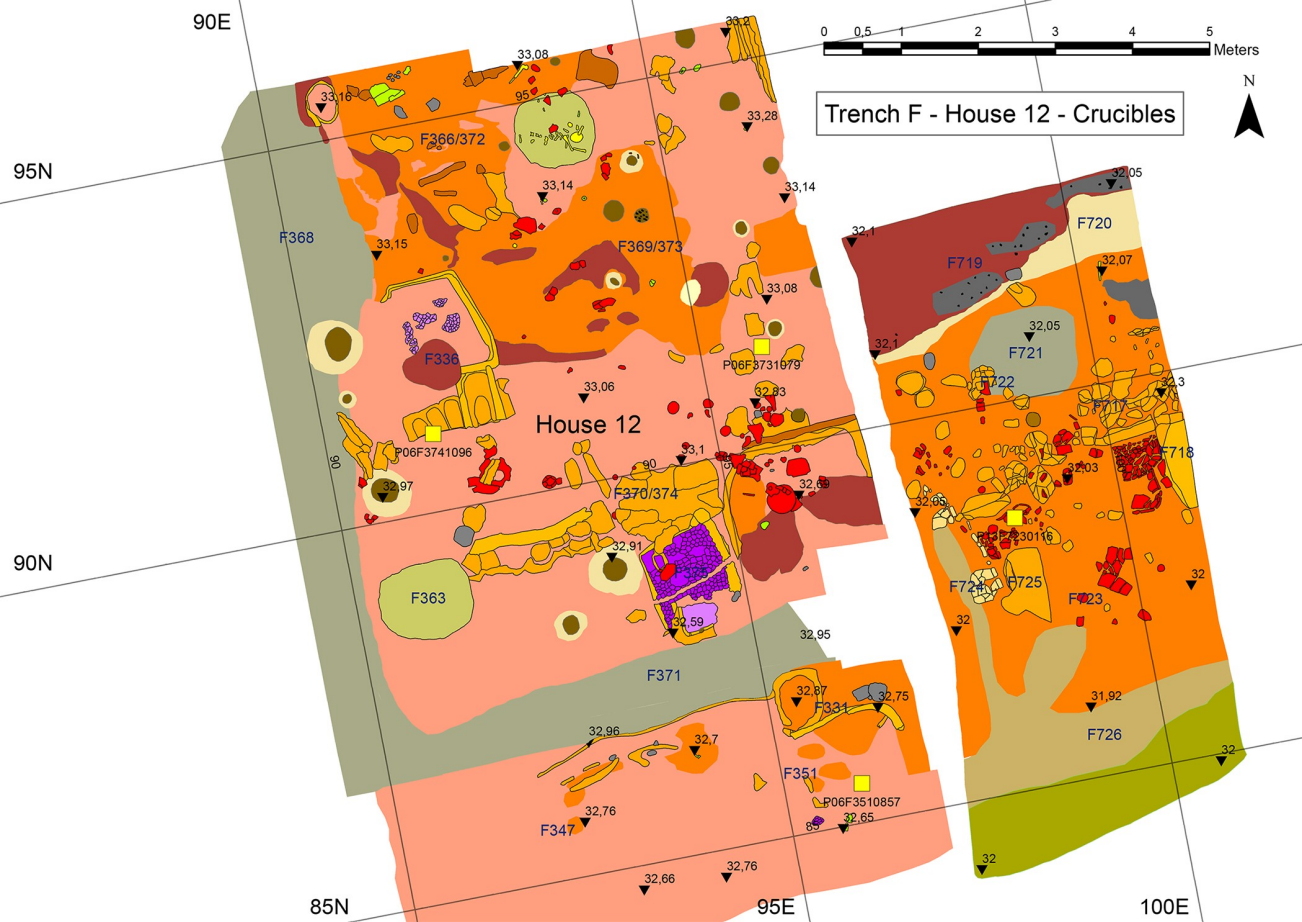
Plan of the context of the three crucibles in the two-storey house (Digital plan O. Joumarin).

**Fig 8:**
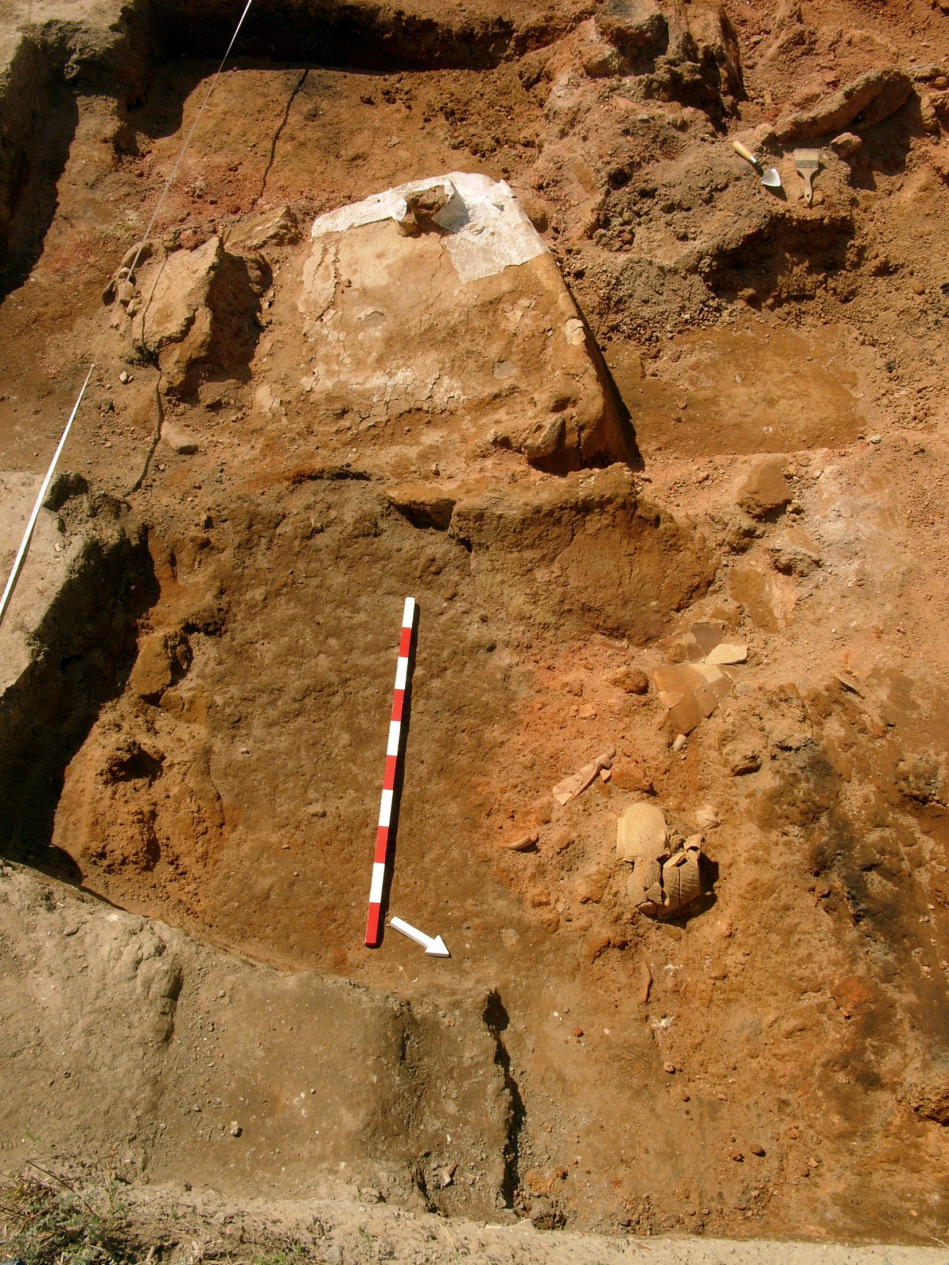
The oven and the surrounding area with the collapsed walls from the east (Photo S. Hansen).

Since the start of the new excavations in 2005, nine crucibles have been discovered (Table 1). Three crucibles were found in the southern part of a two-storey house (House 12) that had burnt down, probably by accident (Figs 7 and 8) [14]. The crucibles were found in the area around the oven on the ground floor; it cannot, however, be testified that they were used in the oven. The inventory of the house is unusually rich, as the finds were sealed under the collapsed massive clay walls. There are no other clear indicators of metalworking in this house.

**Table 1:** Vessels from Pietrele and Blejeşti.

site	find no.	Pb	h	d	m	w	condition	comment
Pietrele	P06F3741096	25.5	4.0	6.4	0.9	40.1	complete	melted mouth and carination
Pietrele	P06B2360140	0.13	2.4	4.6	0.9	19.2	complete	grey patches
Pietrele	P06F3510857	2.3	2.7	3.7	0.6	17.4	complete	no heat traces
Pietrele	P06F3731079	19.5	3.9	5.3	0.9	31.5	complete	no heat traces
Pietrele	P13F7230116	0.14	3.9	4.8	0.9	31.7	complete	heat cracks
Pietrele	P05B1220055	2.3	3.4	4.6	0.9	23.9	in 2 halves	no heat traces
Pietrele	P13F723CER53.4	0.006	3.1	6.6–7.0	0.6	37.3	fragmentary	no lead traces
Pietrele	P12R1190577	1.01	c. 4.4	c. 8.0	c. 0.7	36.9	fragments of half vessel	no heat traces grave find
Pietrele	P07B3830171	6.1	6.5	7.4	0.7	136	complete	no heat traces polished surface
Blejeşti	Blejeşti—1	20.6	4.5	6.1	0.9	94.2	complete	with slag filling
Blejeşti	Blejeşti—2	0.003	4.3	6.1	0.7	60.4	complete	no clean surface

Pb: max. lead content by pXRF in wt% on the surface of the vessels (for discussion see text); h, d, m: max. height, max. diameter, max mouth diameter, all in cm; w: weight in gram.

**Fig 9:**
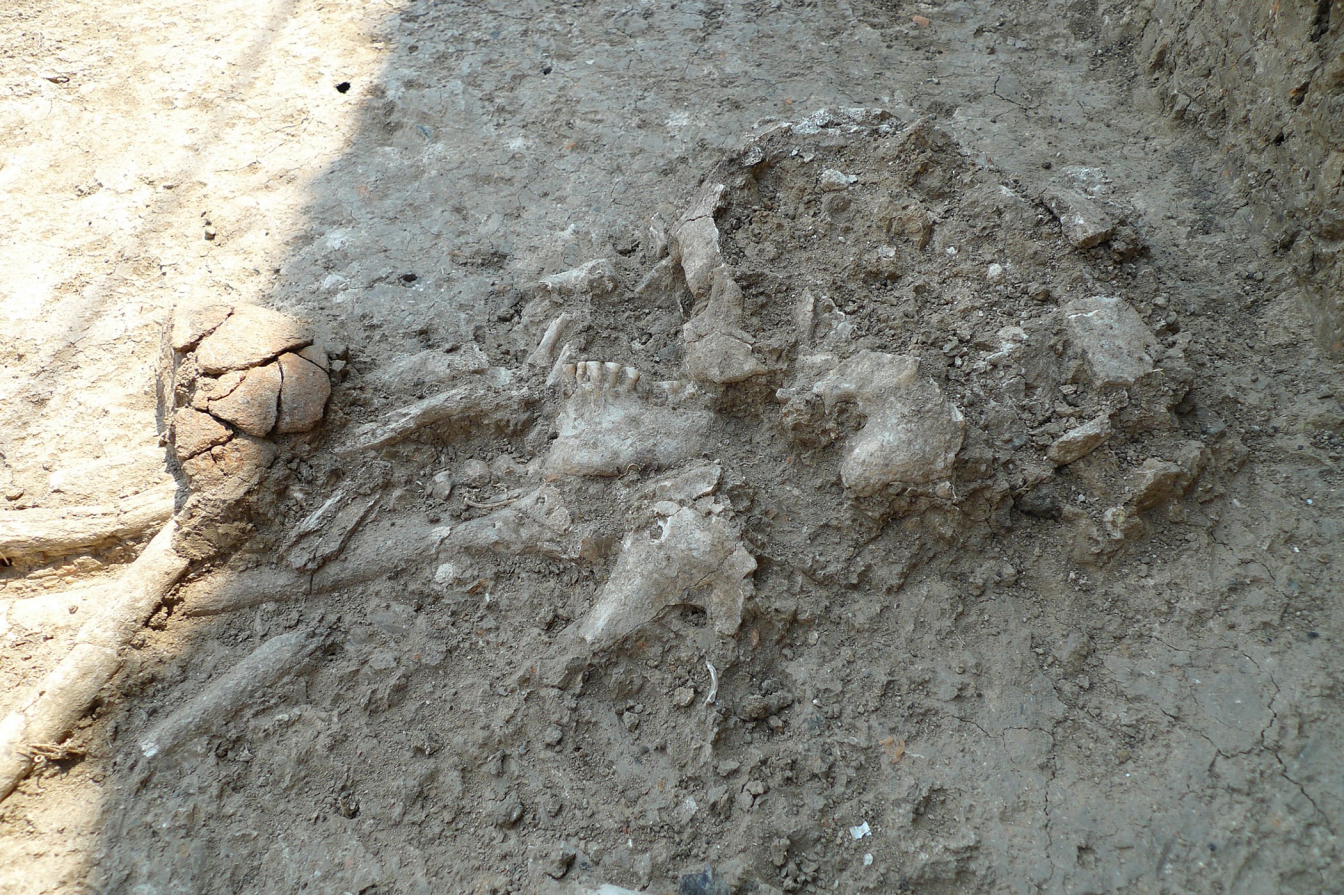
Position of the crucible in the grave R119 (Photo S. Hansen).

**Fig 10:**
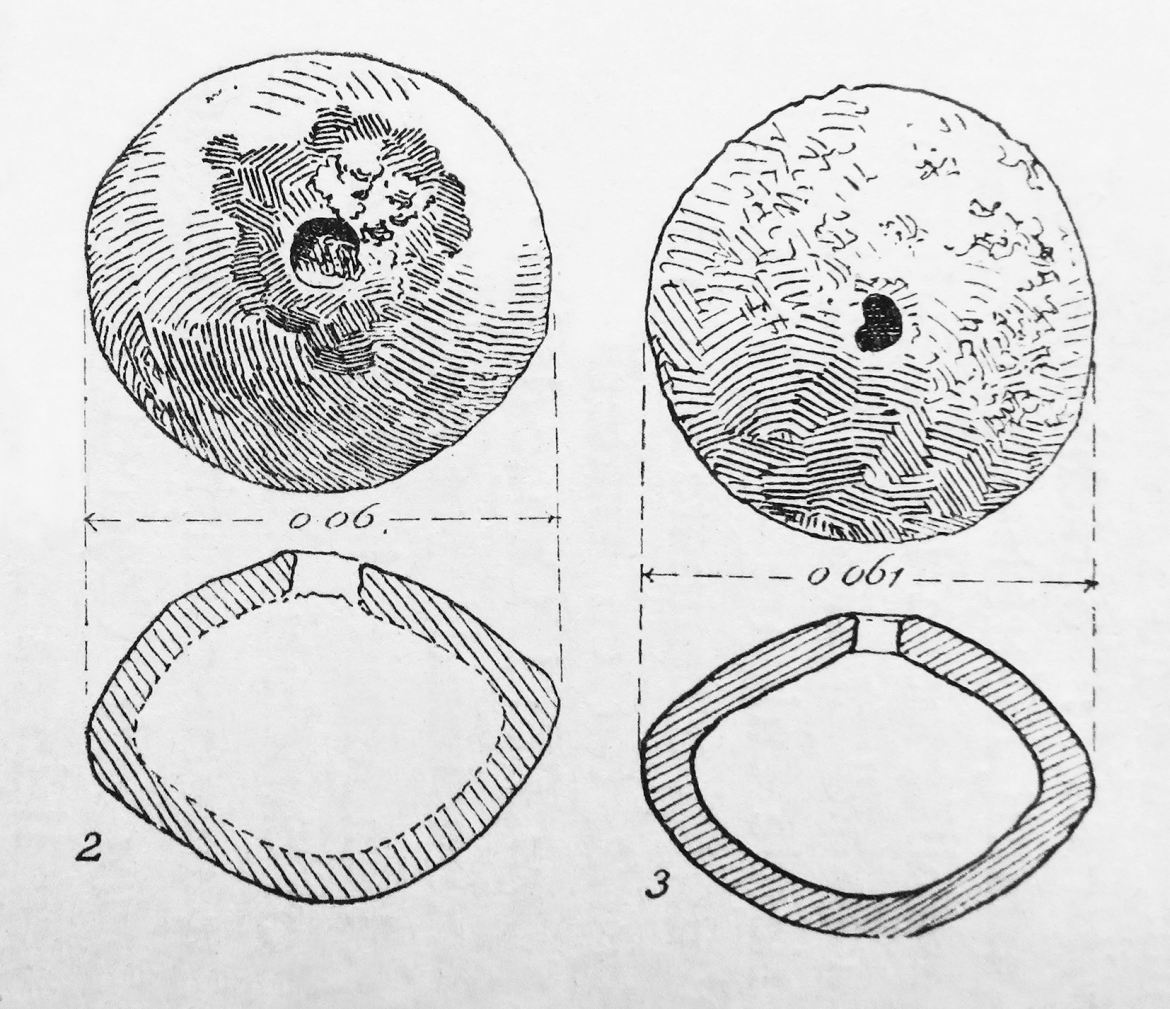
Blejeşti, left: no. 1–2, right: 1–1 (after Berciu 1948).

Two more crucibles (Figs 9 and 10; P12F7230116 and P12F723CER53.4) probably stem from a neighbouring house that was excavated in 2012. These pieces also were discovered in the direct vicinity of an oven.

So far, these two houses have provided the oldest evidence for crucibles. House 12 was dated by radiocarbon and modelling to somewhere between 4410 and 4380 BCE [15]: Bln-5930: 5478±36, cal.: 4450–4250 (charcoal F3 P06/F/366); Bln-5932: 5473±32, cal.: 4370–4250 (charcoal F3 P06/F/372). Two other crucibles found in houses in Trench B show a similar date.

Another find worth mentioning is the crucible fragment P12R1190577, as it appears to be a grave gift (Fig 4 bottom). The deceased was buried in a flexed position facing West–East (Fig 9). The crucible found in this grave had been placed upon or in the left hand in front of the deceased’s skull. The vessel was found already in a broken state, and only this piece had been deposited into the grave. Graves containing vessels that were fragmented or incomplete at the time of burial is typical for burials in Pietrele.

The crucible is a very specific object, and it was by no means chosen by chance. It was taken possibly because the deceased person was experienced in manipulating matter. The deceased interred in Grave R119 is one of the first individuals known thus far who was represented in the grave as a person who dealt with the processing of metal ore.

**Fig 11:**
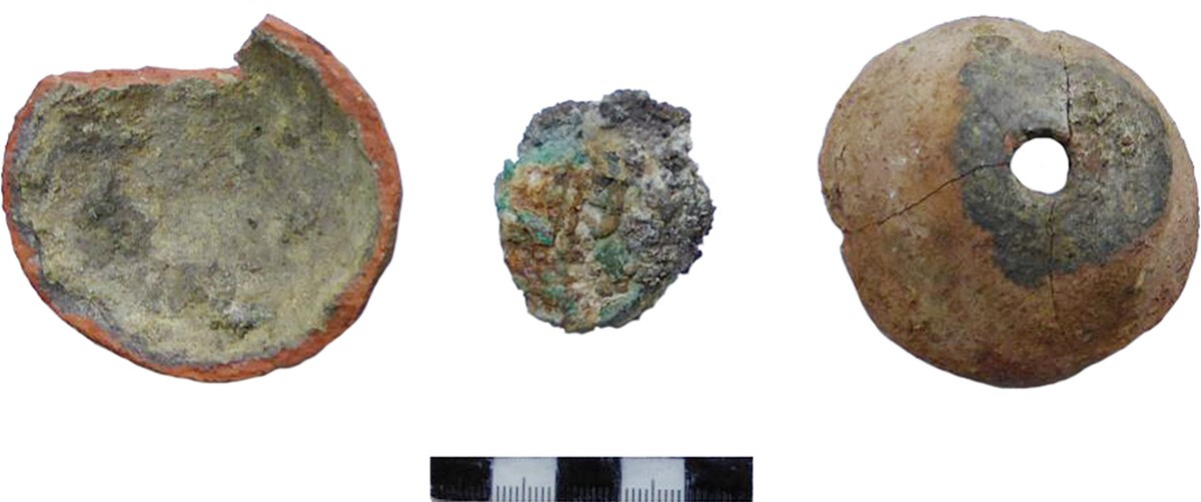
Blejeşti 1–2; left: view inside the vessel’s base; middle: slag-like lump from inside; right: view of the outside of the top of the vessel with the opening and dark patches of slaggy material (Photo D. Steiniger).

For this study, two other finds from a settlement mound in southern Romania were included, namely the two crucibles uncovered in 1948 during excavations in a settlement mound near Blejeşti, jud. Teleorman (Fig 10), west of Pietrele [16]. A piece of slag-like material (or ‘lump’) was found in one of the crucibles (Fig 11), which was analysed by two of the authors (SR and IM) in addition to XRF. The results are described below. The detailed find context is not known, and there are no radiocarbon dates available.

The finds from Pietrele and Blejeşti can be complemented by comparable crucibles from several settlement mounds in Romania and Bulgaria [17], but it is still impossible to get a clear picture from the publications of the dissemination of these crucibles during the 5^th^ millennium, as most of them are probably still unpublished. The Copper Age necropolis of Vărăști, belonging to the Gumelniţa culture, should be mentioned here as well: biconical objects were found in ten graves from this site [18].

The significance of these finds is obvious; for the first time, metallurgical activities could be detected in a settlement mound. This is, moreover, the first time the use of lead ore could be proven in southeastern Europe during the 5^th^ millennium BCE. It seems clear that the crucibles with lead traces were not limited to one or two villages, but instead were widespread among the Lower Danube area and beyond. Further investigations are needed in order to gain more analytical data from other find spots.

Along with copper and gold, silver was likely also used as early as the 5^th^ millennium, as indicated by finds from Greece and Moravia [19]. The silver disc from Kotouč near Štramberk in Moravia (21.4 cm in diameter) is likely the earliest silver object in Central Europe, typologically dated to the late 5^th^ millennium BCE [20]. Small silver discs from Greece probably also belong to the 5^th^ millennium [21]. There is a lack of analytical data necessary to determine whether the silver finds from the 4^th^ millennium were made from native silver or from lead ores using cupellation [22].

Lead had been utilised in the Near East since the Late Neolithic. The bracelet from layer 12 in Yarim Tepe I [23] and a piece of lead from house TT6 in Arpachiyah [24] are worth mentioning for its use during the 6^th^ millennium BCE.

Recently, a lead object from the Ashalim cave in the northern highlands of the Negev was reported [25]. It is a double conical object, 3.7 cm in height and weighs 156 g with a wooden shaft 22.4 cm in length. Radiocarbon dates suggest that the object was used between 4300 and 4000 BCE. The lead isotopes accord well with those of the Taurus mountains. Far-reaching contacts to the Taurus and even the Caucasus can also be traced through the metal composition of the more complex cast objects in the Nahal Mishmar cave [26–28]. The finds in Nahal Mishmar, dated to the last quarter of the 5^th^ millennium BCE, show a large variety of alloys. The assemblage could almost be seen as a laboratory of a metal worker experimenting with different metal compositions.

Lead from the 5^th^ millennium has not been recorded yet for southeastern Europe, and it appears to have been missing during the 4^th^ millennium. The crucibles from Pietrele and Blejeşti are the first pieces of evidence for the use of lead ore in southeastern Europe, though there could be one earlier case from the Vinča settlement of Donja Tuzla, where grey powder detected in a normal pot was determined to be lead sulphide [29].

We do not have clear evidence for the purpose of smelting the lead ore in Pietrele and Blejeşti; however, the prescientific and alchemistic dimensions of metalworking activities should be regarded as important for every advancement in metal technology. The transformation of matter in fire had been observed long before metal was produced [30]. The metalworker experimented with all kinds of substances and observed every change by adding different metals with different colours to others. This was the path from pure copper objects to copper alloys.

## Materials and methods

The decision to use XRF was based on the importance of keeping the samples and ‘crucibles’ intact. There is also a responsibility for archaeological finds in general, as they are objects of cultural heritage and are extremely scarce. As there are several well-known and crucial problems involved in using a p-XRF device–especially when analysing prehistoric ceramic and slag-like material without first homogenising and pulverising it–a short discussion of the limits and possibilities of the method is required (see: S1 Supporting Information). Although XRF is often used only to provide qualitative data, a comparison with results from SEM indicates that XRF yield qualitative measurement results with a variable bias that can also be used to sort the samples according to higher or lower amounts of metal traces.

Two samples–one consisting of a section of the wall of the Blejeşti 1–1 vessel and the other of the lump Blejeşti 1–2 found inside it–were analysed with the Scanning Electron Microscope (SEM) to determine the chemical composition of the constituent phases and their distribution in the sample (Hitachi 3400n Type II, with microanalyser EDAX Bruker Quantax 4010, MICROLAB Laboratory of the Instituto de Historia, Centro de Ciencias Humanas y Sociales, CSIC, Madrid). In working conditions, the accelerating voltage of 20kV or 30kV was used, depending on the matrix effect, scanned area to analyse, etc. Both samples were mounted using bi-component autopolymerising resin (methyl methacrylate) and suitably polished using an automatic polishing machine (PRESI Mod. Mecapol P230, with P.E.R.U. arm, MICROLAB Laboratory). Metallisation was with graphite; the error in semiquantitative determinations is variable and very dependent on the matrix effect of the material in the scanned area. Thus, for example, the Pb measured in litharge or galena has approximately 6% error. On the other hand, when this element is found in smaller quantities, the error drops below 1%. The mineralogical composition was determined by X-ray diffraction (XRD, Bruker AXS D8 diffractometer equipped with a Co X-ray tube, Goebel mirror optics, and a LynxEye Linear Position Sensitive Detector for ultra-fast XRD measurements). A current of 30 mA and a voltage of 40 kV were employed as tube settings. The XRD data were collected over a 2θ range of 10–120° with a step size of 0.015°. In this study, the version 4.2 of Rietveld analysis program TOPAS, Bruker AXS, was used for the quantification of crystalline phases. In order to eliminate the instrumental contribution to peak broadening, instrument functions were empirically parameterised from the profile shape analysis of a corundum sample measured under the same conditions (Laboratorio de Difracción de Rayos X, CENIM-CSIC, Madrid). One of the samples has also been investigated by optical microscopy (Leica DMML with a digital photo camera DFC 480, MICROLAB Laboratory).

## Formal description and initial pXRF measurements of the vessels from Pietrele and Blejeşti

The general biconical shape, in addition to the roughly similar size and weight, are features that are common to all ‘crucible’ vessels with traces of lead, which were analysed with p-XRF and state-of-the-art laboratory methods (Table 1).

The lead values measured on the surface of the vessels range between ca. 26 and 20 wt% maximum, while on other vessels it remained only at around 2 or 0.1 wt%. This is nonetheless much higher than the mean Pb value of normal pottery at e.g. Pietrele, which is around 30–60 ppm. These values are, of course, for informative purposes only, as the surface analysis with p-XRF gives an estimate of the chemistry on the measured surface, which also underwent various alteration processes during prehistoric use and deposition as well as during its post-depositional phase.

The fragmentary and partially eroded state of vessel P13F723CER53.4 and a very thick layer of gypsum on the surface of the vessel Blejeşti-2, are most probably responsible for the lack of Pb traces in these measurements. The p-XRF also detected a certain amount of trace elements besides Pb in the slag-like outer surfaces of some vessels from Pietrele (and in the Blejeşti-1 slag), for example, Zn and Cu. These trace elements will not be discussed here in detail. They indicate most probably a polymetallic sulphidic ore body containing copper and zinc in addition to lead sulphides. A common ore source for both Pietrele and Blejeşti samples is confirmed by LIA, as described below.

Weight seems to be another distinctive property that enables us to identify small vessels which may still contain remains of slag-like material. The slag in the Blejeşti-1 vessel weights 46.6 g the vessel itself 47.6 grams (totalling 94.2 g). We have to consider that some melted material had probably already been poured out before the remaining material solidified near the mouth inside the Blejeşti-1 vessel. All ‘empty’ but complete vessels from Pietrele weigh less than ca. 40 grams, except for P07B3830171, which does not seem to be one of the ‘crucibles’, since it has different features.

**Fig 12:**
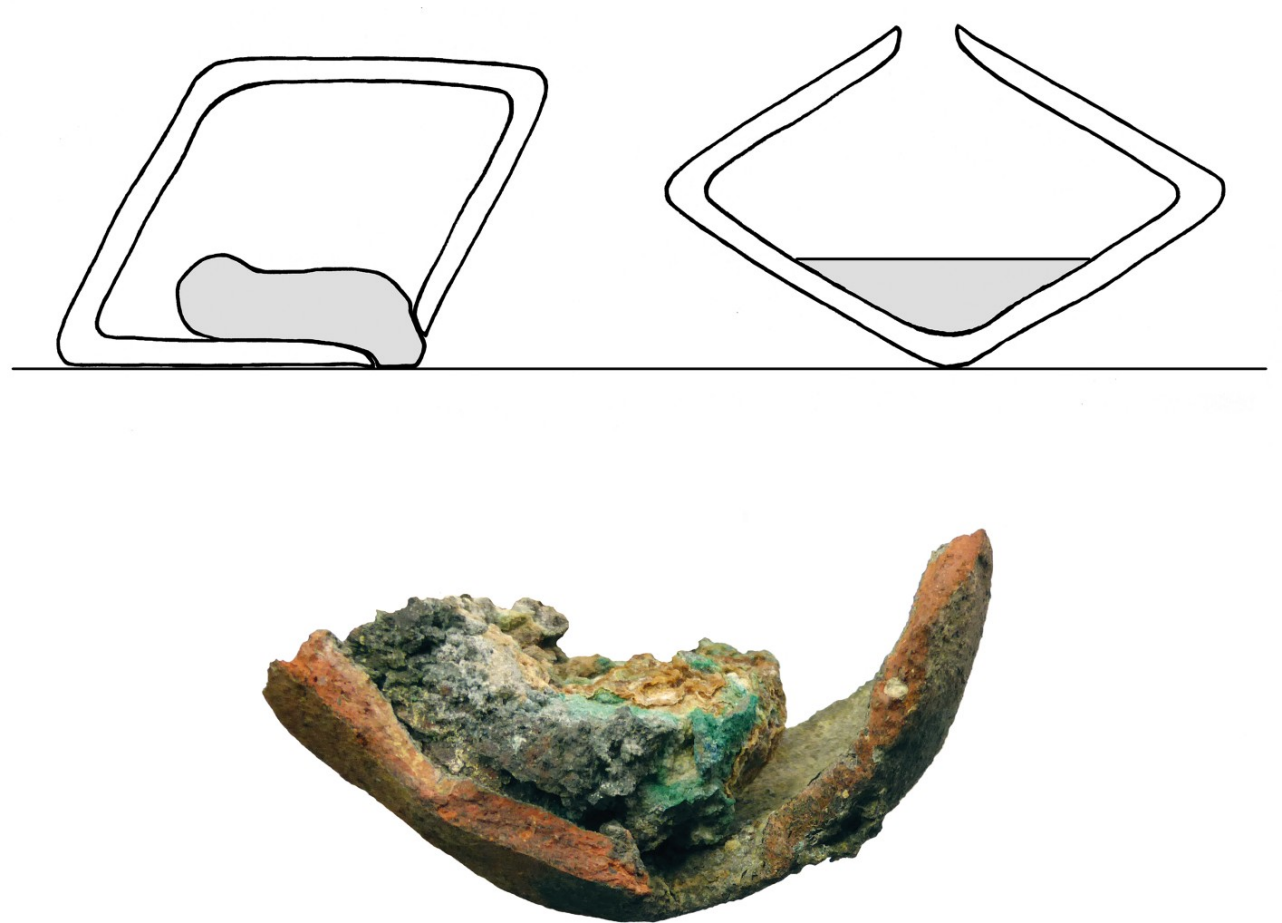
Left: sketch of a section of the ‘crucible’ vessel Blejeşti-1 as found, with the slag-like material at the mouth and upper half of the vessel (as seen in the photo below). It seems it solidified while being poured out. Right: expected position of the vessel and slag during firing (Sketch and photo D. Steiniger).

Concerning the Blejeşti-1 vessel, the discovery of the slaggy material inside provided further insight into the technical process beyond chemical analytics. The slag-like material itself solidified exactly in the moment when it was poured out of the mouth, and the upper half was partially filled with it, while at the base was none (Fig 12). Therefore, it seems that the vessel was turned over to let the melt flow out and would have rested at the base during firing. This fact also helps us to understand the biconical shape of the ‘crucibles’. When they were standing upright in a fire, they would hold this position by being pressed slightly into the ash and charcoal. In this way, additional heat could–in all probability–be applied to the hole with the aid of blowpipes, which is indicated by melted clay around the mouth and rim of some crucibles. By tipping such a vessel over at the top, which could be done with a simple stick, the shape in any case would cause it to lie on the upper side, so that the melt could flow out. No special handles or pincers would have been necessary because the shape of the containers was optimised for that purpose.

Traces of heat in the form of melted clay but also slaggy areas were visible only on some vessels. Dark patches due to secondary firing and in some cases characteristic cracks in the clay are additional features. But some vessels with absolutely no macroscopically visible traces of heat or slaggy material nevertheless gave Pb values of up to ca. 20 wt%. One vessel shows black slaggy material at the mouth and the carination (Fig 3 top), where it seems that the clay melted completely, so that melt from inside penetrated through to the surface. This happened at the carination most probably because that was the vessel’s weakest point–where the upper and lower halves were joined together somewhat imperfectly, including the charge, before it all was placed into the fire.

One vessel (Fig 6) differs from the aforementioned vessels in its shape, material and surface. While the others resemble ‘crucibles’, often with traces of heat or slag-like material and a rough brown clay surface–without any further treatment, this particular vessel, P07B3830171, is made of fine, beige-coloured clay with a polished surface. Its general shape follows somewhat that of the other ‘crucibles’, since it also has a hemispherical base and a more conical upper half, but here it is asymmetrical in form with a rather distinct shoulder and two vertically perforated small knobs opposite each other at the broadest width. It also has a distinctive flat base to stand on, while the others have round bases. Around the mouth is a distinct upper circular rim, surrounded by a small flat and oval-shaped carination; perhaps a lid fit here that closed the vessel. It was possible to recover some dust from inside the vessel, which microscopically contained small slag-like particles of less than mm-size. This material also contained lead when analysed with p-XRF (up to ca. 6 wt%). In the near future, laboratory methods will provide us with further insight into the lead-rich material that was stored in this vessel. Namely, it seems that it may have contained the ‘final product’, which was produced in the ‘crucibles’–whose general shape is copied approximately by this fine vessel, which in itself seems to be a ‘premium version’ of the rough crucibles.

## The leaded lump Blejeşti 1–2

**Fig 13:**
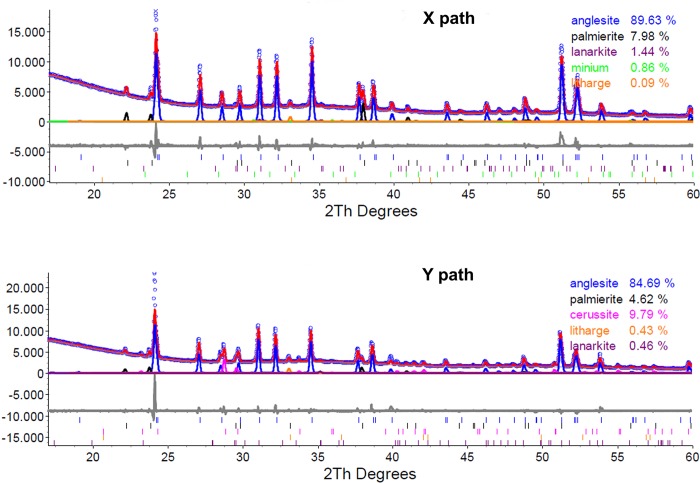
Diffraction patterns of the leaded lump Blejeşti 1–2 (S. Rovira).

Non-destructive XRD analysis was performed on the polished surface of a section of the lump according to two perpendicular paths (X and Y). Fig 13 shows the results of both analyses, which show that the most abundant mineral is anglesite (lead sulphate) followed by palmierite (potassium sulphate). Lanarkite (lead sulphate) and litharge (lead oxide) are found in low concentrations. Cerussite (lead carbonate) has been detected in the analysis of the Y path, probably due to the fact that this direction crosses a thick superficial crust (that is not in the X path), in which carbonate may have been formed by weathering over the course of time. Minium in X path may also be due to the same phenomenon.

Research using scanning electron microscopy of a lead-rich material is sometimes problematic not because of the difficulty in measuring the content of the metal, which appears perfectly identified in the spectrum, but because of the lack of contrast of the screen image to differentiate two contiguous areas with slight differences in lead content when working with backscattered electrons mode. The high atomic number of this element hinders the contrast between different compounds with similar lead composition. In this case, however, the main components of the material and their spatial distribution have been observed without difficulty. The results of the analysis of the phases observed in different areas of the sample are shown in Table 2.

**Table 2:** Phase compositions of the leaded lump Blejeşti 1–2.

Analysis #	Phase	Al_2_O_3_	P_2_O_5_	K_2_O	FeO	CuO	PbO	SO_3_	S	Pb	Cu
Blejesti 1-2/01	White phase (Anglesite)	nd	nd	nd	nd	nd	75.7	24.3			
Blejesti 1-2/02	Dark grey phase (Palmierite)	0.5	nd	18.7	nd	1.5	47	32.2			
Blejesti 1-2/04	White phase (Anglesite)	0.2	nd	nd	nd	nd	75.1	24.7			
Blejesti 1-2/05	White phase (Anglesite)	nd	nd	nd	nd	nd	76.6	23.4			
Blejesti 1-2/06	Dark grey phase (Palmierite)	nd	nd	20.3	nd	nd	49	30.7			
Blejesti 1-2/07	Bulk analysis (lump)	2.4	0.9	3.2	1.4	1.4	67.3	22.5			
Blejesti 1-2/08	Grey phase (Palmierite)	nd	nd	21.3	nd	nd	46.3	32.4			
Blejesti 1-2/09	Light grey phase (Anglesite)	nd	nd	2.5	nd	nd	73.5	24			
Blejesti 1-2/10	Light grey phase (Litharge)	nd	nd	nd	nd	nd	100	nd			
Blejesti 1-2/11	Light grey phase (Litharge)	nd	nd	nd	nd	nd	100	nd			
Blejesti 1-2/12	Light grey phase (Litharge)	nd	nd	nd	nd	nd	100	nd			
Blejesti 1-2/13	Light grey phase (Anglesite)	nd	nd	nd	nd	nd	75.9	24.1			
Blejesti 1-2/14	Grey phase (Palmierite)	nd	nd	20.3	nd	nd	48.8	30.9			
Blejesti 1-2/15	Inclusion (lead sulphide)								12.6	87.4	nd
Blejesti 1-2/17	Nucleus in inclusion (Anglesite)	nd	nd	nd	nd	2.3	71.6	26.1			
Blejesti 1-2/18	Inclusion (lead sulphide)	nd	nd	nd	nd	nd	nd	nd			
Blejesti 1-2/16	Inclusion (lead sulphide)	nd	nd	nd	nd	nd	nd	nd			
Blejesti 1-2/19	Light grey around inclusion (Litharge)	nd	nd	nd	nd	nd	100	nd			
Blejesti 1-2/20	Dark grey near inclusion (Anglesite)	nd	nd	nd	nd	nd	75.2	24.8			
Blejesti 1-2/30	Inclusion (upper part, lead sulphide)								18.5	79.9	1.6
Blejesti 1-2/31	Inclusion (upper part, lead sulphide)								14.1	85.1	0.8
Blejesti 1-2/32	Inclusion (lower part, lead sulphide)								15.4	84.6	nd
Blejesti 1-2/37	Dark grey around inclusion	nd	nd	nd	nd	8.3	75.6	16.1			
Blejesti 1-2/38	Dark grey around inclusion	nd	nd	nd	nd	19.3	47.2	33.4			

SEM microanalysis, in wt. %, nd not detected.

**Fig 14:**
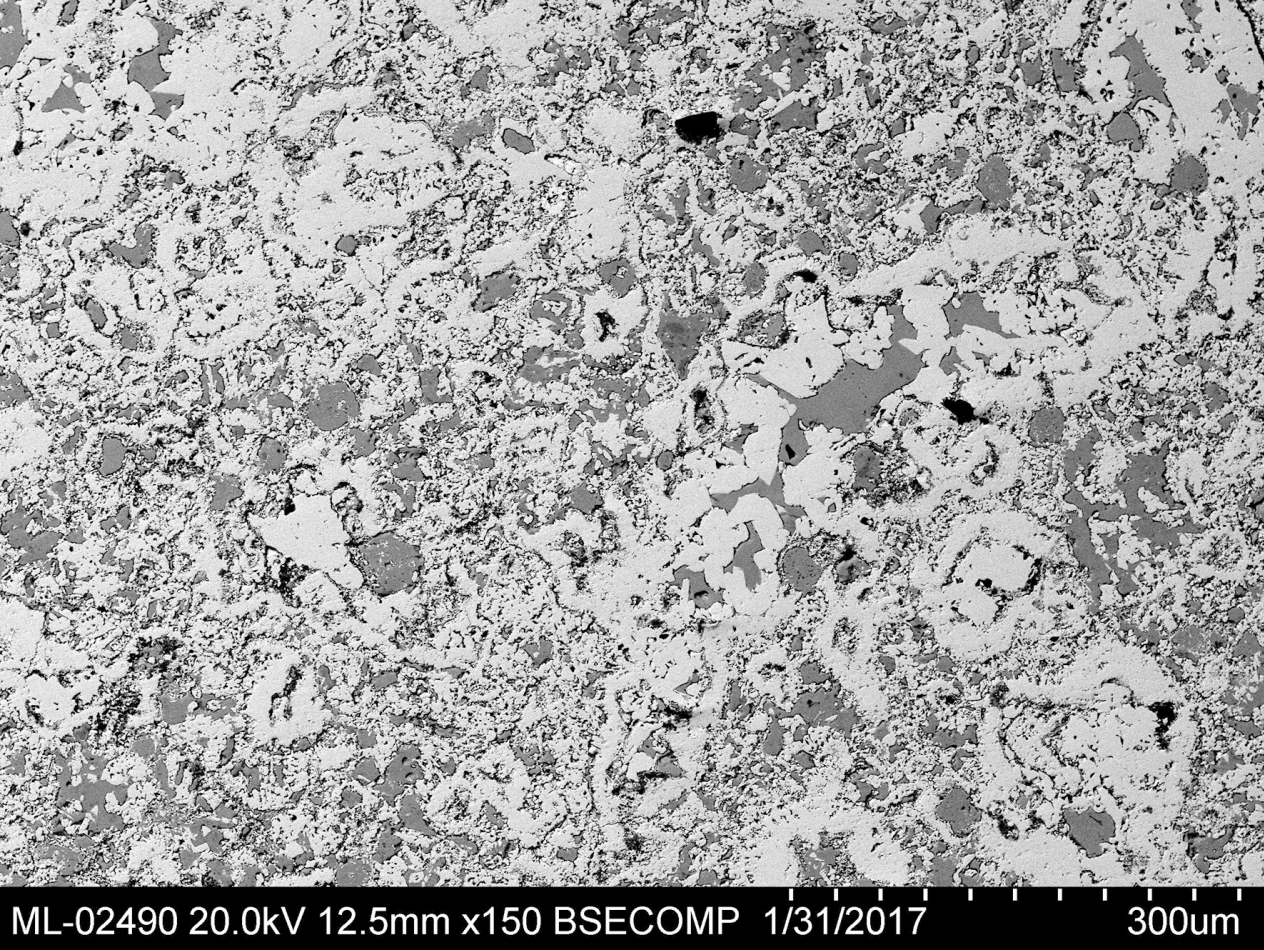
Aspect of the phase distribution in the core of the leaded lump Blejeşti 1–2. The white phase is anglesite, the grey phase is palmierite. SEM image, backscattered electrons (S. Rovira).

The core of the sample is basically composed of anglesite and palmierite; Fig 14 shows a zone of the core. The white phase is anglesite (Blejeşti 1-2/01, /04 and /05 in Table 2) and the grey phase is palmierite (Blejeşti 1-2/02 and /06).

**Fig 15:**
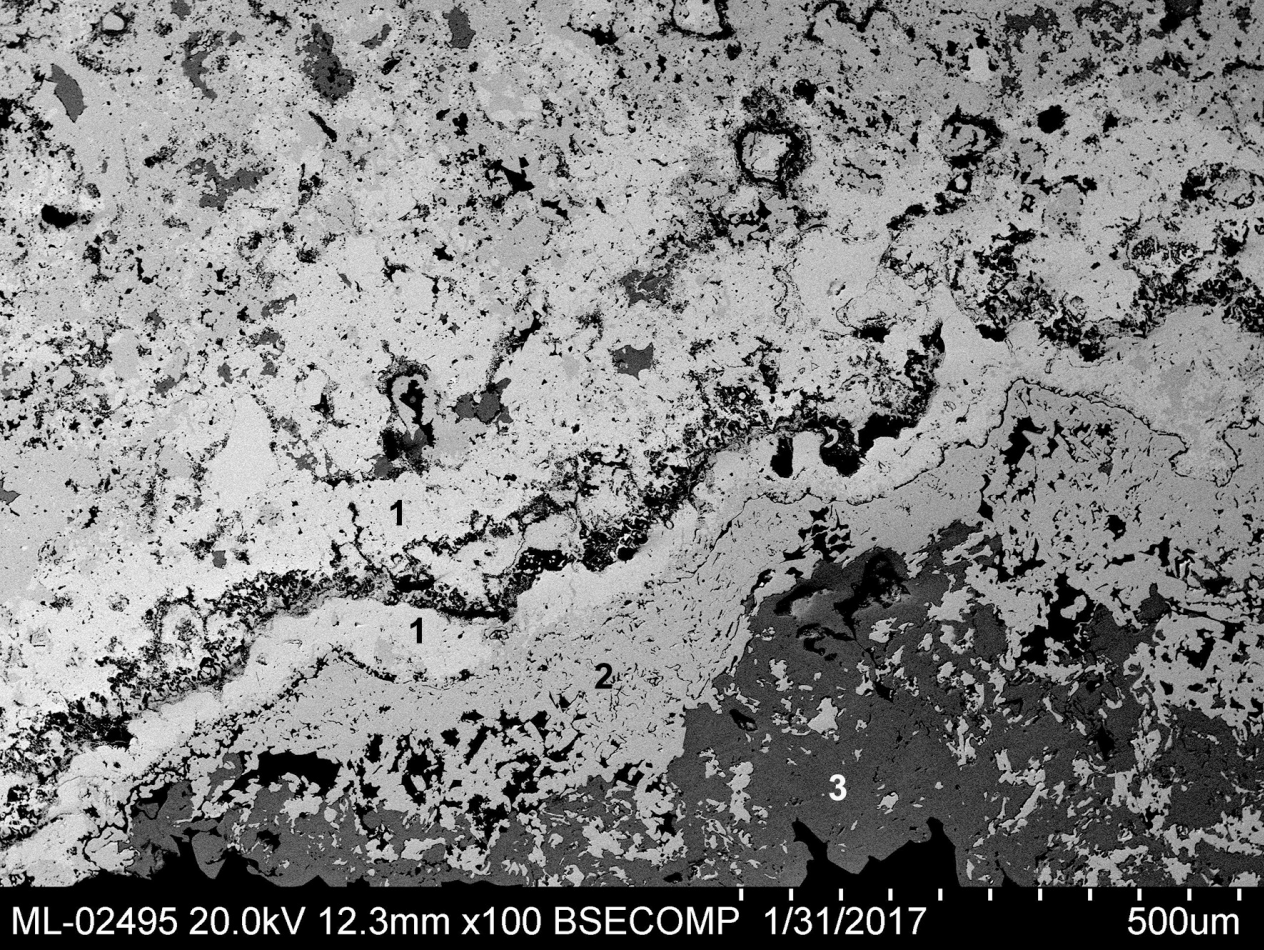
Part of the lump Blejeşti 1–2 corresponding to the superficial crust. 1 lead oxide (litharge), 2 lead sulphate (anglesite), 3 potassium-lead sulphate (palmierite). SEM image, backscattered electrons (S. Rovira).

In some parts of the sample, a crust approximately 0.5 mm in thickness is preserved. It consists of at least three layers of different lead compounds. The most internal is lead oxide (litharge, Blejeşti 1-2/10, /11 and /12). On it has formed a layer of anglesite (Blejeşti 1-2/13). Finally, the outermost layer is practically pure palmierite (Blejeşti 1-2/14). The structure of the crust can be seen in Fig 15.

**Fig 16:**
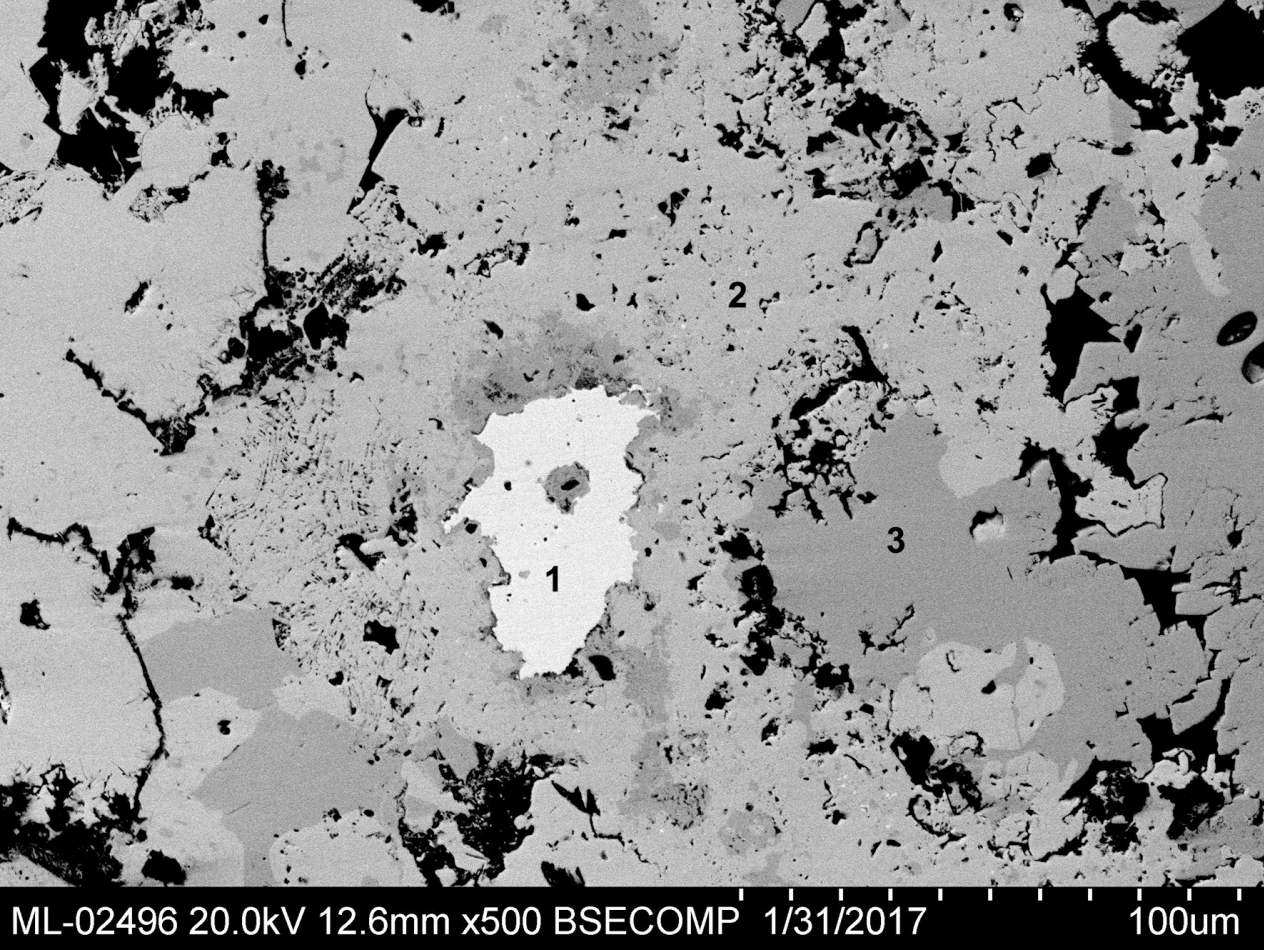
Lead sulphide inclusion in the lump Blejeşti 1–2. 1 galena, 2 litharge, 3 anglesite. SEM image, backscattered electrons (S. Rovira).

**Fig 17:**
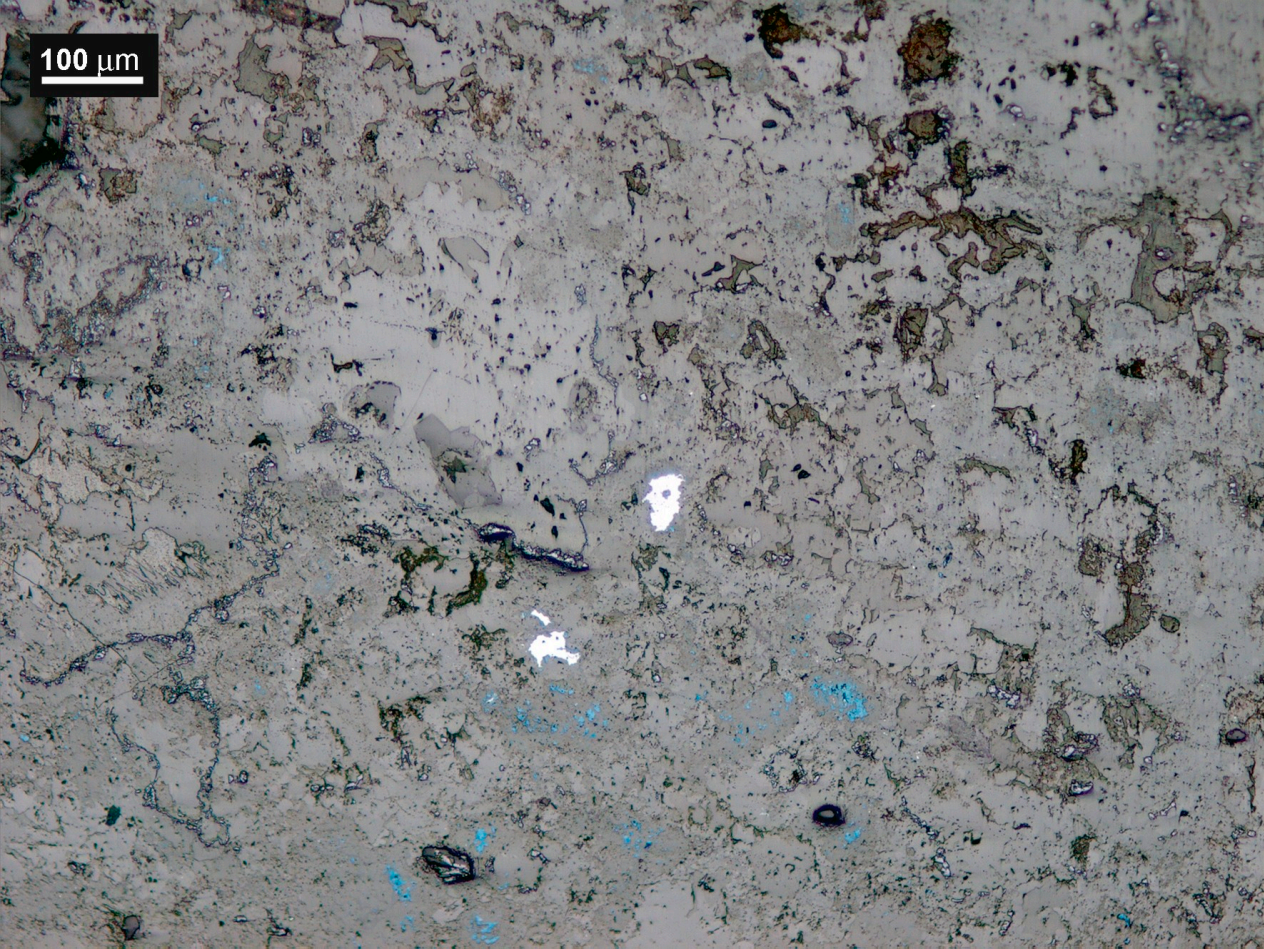
Blue lead sulphide in the lump Blejeşti 1–2, near galena inclusions. OM, bright field (S. Rovira).

A few scattered lead sulphide inclusions were found (Blejeşti 1-2/15, /30, /31 and /32). The irregular edges of these inclusions indicate that they are residual grains of galena (not of secondary sulphide formations, which usually acquire rounded shapes) (Fig 16). The optical microscopy (OM) also confirms the existence of sulphides in the lump, which appear as bluish dots scattered on the field (Fig 17). Galena has not been detected in the XRD analysis, because very few traces remain in the sample; their sizes are probably below the detection limits of the instrument used, or perhaps they are absent in the paths tracked for analysis.

Although the remains of galena are very scarce, their finding is crucially important for to understand the chemical processes that took place inside the closed vessel in which the leaded lump was formed. For this reason we have paid special attention to the study and characterisation in the SEM of the inclusion of a larger size (not exceeding 50 μm in its greater length). In addition, other data suggest that the inclusion in question is the mineral galena and not a secondary sulphide. It is significant that it is not a pure lead sulphide, but is accompanied by a small amount of copper (see analyses Blejeşti 1-2/31 and /32 in Table 2). Galena outcrops also often contain other metallic sulphides, copper and zinc being the most frequent. Copper salts accumulated around the inclusion (dark grey areas surrounding it, Fig 16), which is a clear indication that they were produced by oxidation of the copper sulphide in the original ore (Blejeşti 1-2/37 and /38).

**Fig 18:**
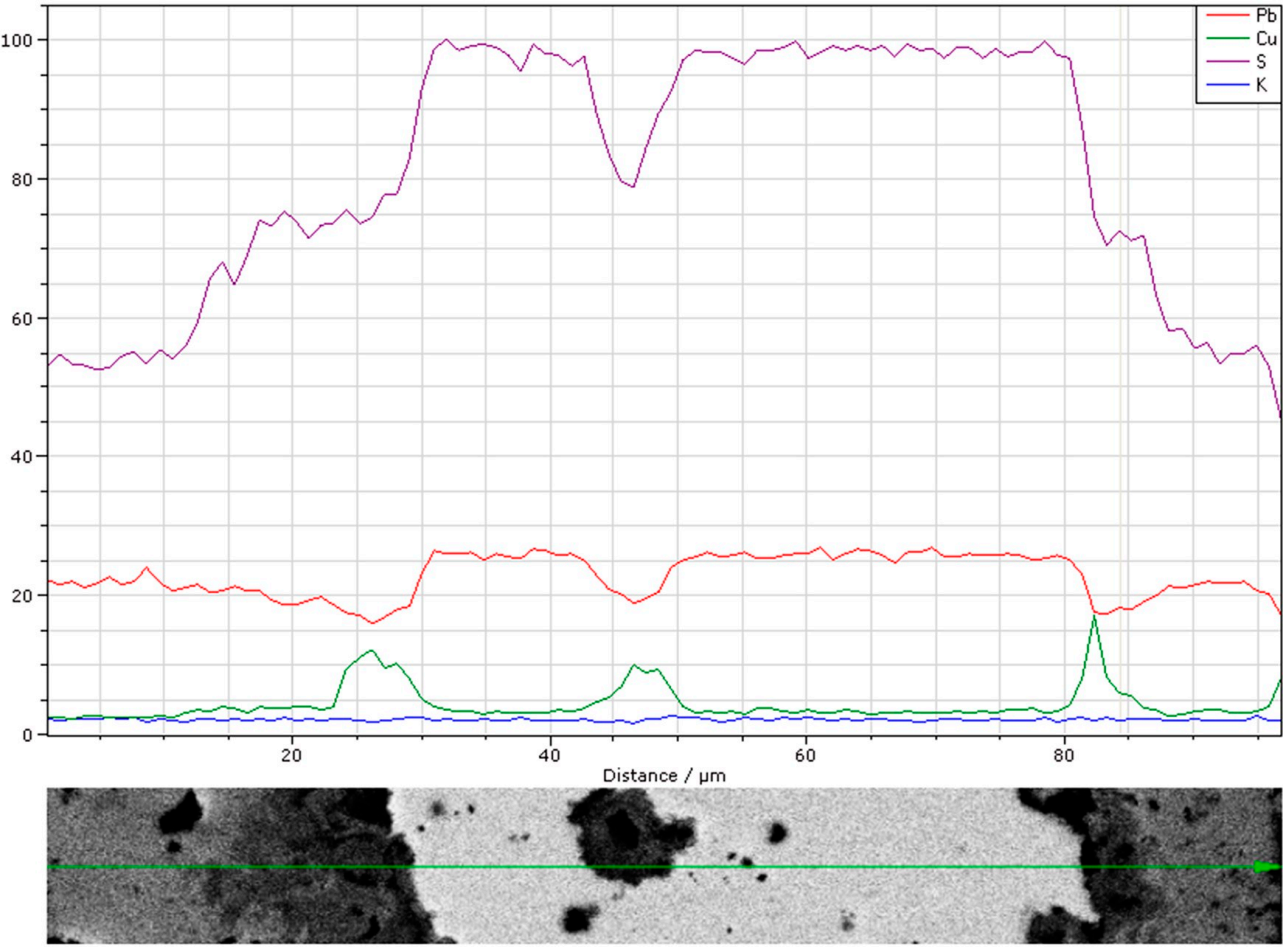
Line scan for the relative concentration of S, Pb, Cu and K in the inclusion of galena and its surroundings in Blejeşti 1–2. Explanation in the text. SEM analysis (S. Rovira).

A line scan through the galena inclusion and neighbouring regions has been performed for elements S, Pb, Cu and K. The corresponding graph draws the relative heights of the contents of those chemical elements along the line (Fig 18). As we see, the copper compounds accumulate at the edges of the inclusion, confirming what has already been suggested by the microprobe analyses carried out on these regions. The total amount of copper in the core of the lump (analysed as CuO) is 1.4%, which is a significant figure (Blejeşti 1-2/07).

## The vessel Blejeşti 1–1

The ceramic vessel Blejeşti 1–1 that held the leaded lump inside shows signs of glazing throughout its internal surface. To study the chemical and mineralogical composition of this layer, a small sample was cut and its section studied using SEM. The semiquantitative analytical results are presented in Table 3.

**Table 3:** Phase composition of the glassy layer on the inner surface of the vessel Blejeşti 1–1.

Analysis #	Phase	MgO	Al_2_O_3_	SiO_2_	P_2_O_5_	Na_2_O	K_2_O	TiO_2_	CaO	FeO	CuO	ZnO	PbO
Blejesti 1-1/01	White phase (lead oxide)	nd	nd	nd	nd	nd	nd	nd	nd	nd	nd	0	100
Blejesti 1-1/02	Grey inclusion	nd	nd	nd	nd	nd	nd	nd	nd	76.4	nd	15.6	8.0
Blejesti 1-1/03	White phase (lead oxide)	nd	nd	nd	nd	nd	nd	nd	nd	nd	nd	nd	100
Blejesti 1-1/04	Grey inclusion	nd	6.4	0.7	1.3	nd	nd	0.6	nd	56.1	2.3	12.7	18.7
Blejesti 1-1/05	Grey inclusion	nd	3	1.1	nd	nd	nd	nd	nd	64.3	1.6	17.3	12.7
Blejesti 1-1/06	Grey inclusion	nd	nd	nd	nd	nd	nd	0.6	1.4	26.6	nd	4.8	66.6
Blejesti 10-/07	White crystal in geoda (lead oxide)	nd	nd	nd	nd	nd	nd	nd	0.6	nd	nd	nd	99.4
Blejesti 1-1/08	Wall of geoda (lead oxide)	nd	nd	0.4	nd	nd	nd	nd	nd	nd	nd	nd	99.6
Blejesti 1-1/09	Grey inclusion	nd	1.8	1.1	0.3	1.4	nd	0.5	nd	69.8	0.7	15.5	8.7
Blejesti 1-1/10	Grey inclusion	nd	3.5	nd	nd	0.6	nd	nd	1.2	66.3	1.8	15.9	10.7
Blejesti 1-1/11	Bloated ceramic below white phase	2.1	14.4	25.8	0.7	nd	2.1	0.6	1.5	13.7	2.0	7.0	30.0
Blejesti 1-1/12	Ceramic fabric (bulk analysis)	2.1	36.7	43.0	nd	0.4	4.2	1.4	2.5	9.6	nd	nd	nd

SEM microanalysis, in wt. %, nd not detected.

**Fig 19:**
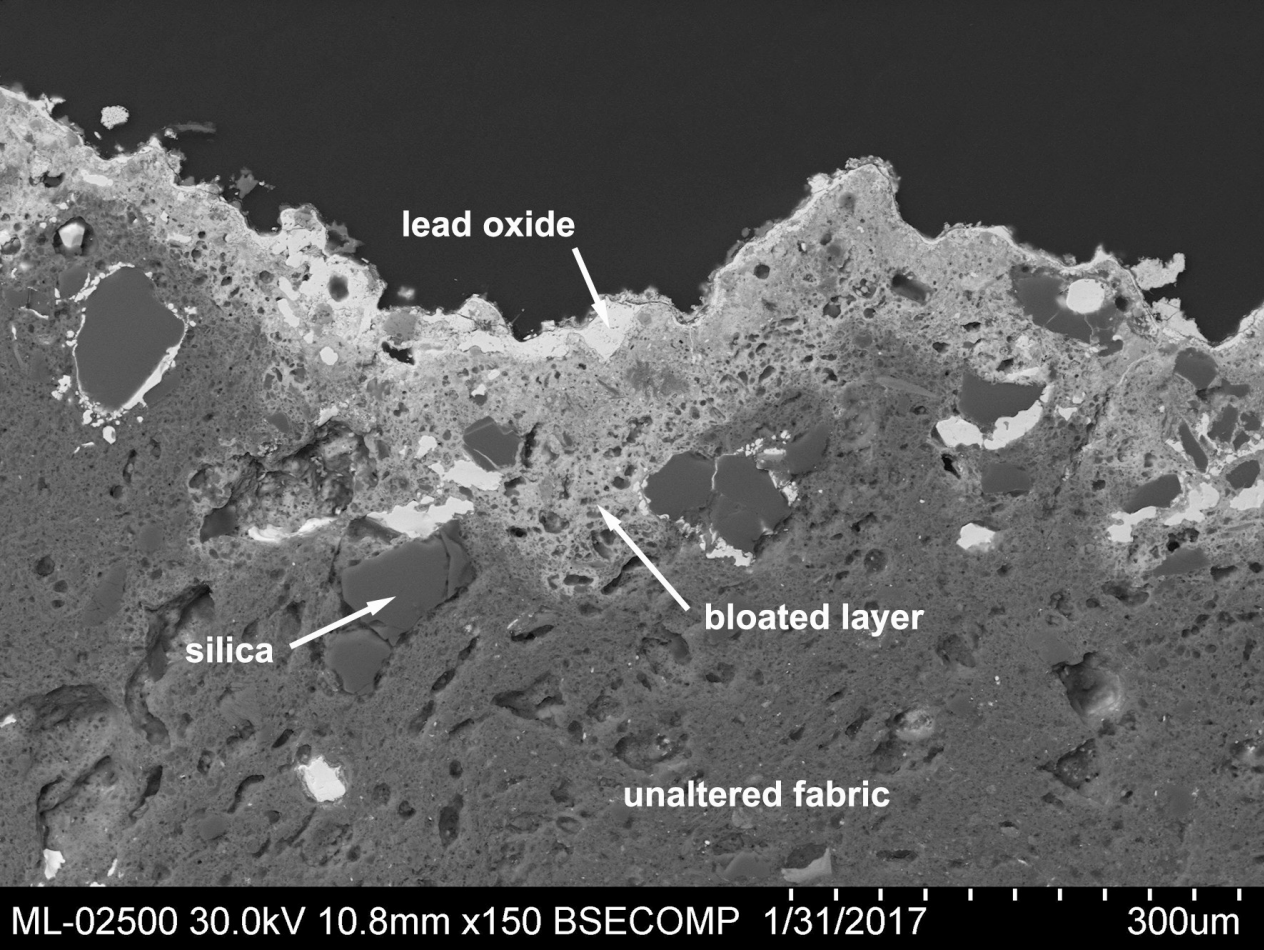
Section of the Blejeşti 1–1 piece showing the location of the main structural phases. SEM image, backscattered electrons (S. Rovira).

The outermost surface layer consists of a continuous, very thin film of lead oxide (Blejeşti 1–1 / 01 in Table 3, Fig 19). Under this layer is a thicker one with abundant vacuoles caused by the thermal effect on the ceramic fabric and by the reaction of the silicates with the lead compound. As is well known, lead oxide readily reacts with the silicates at a high temperature to form glass.

**Fig 20:**
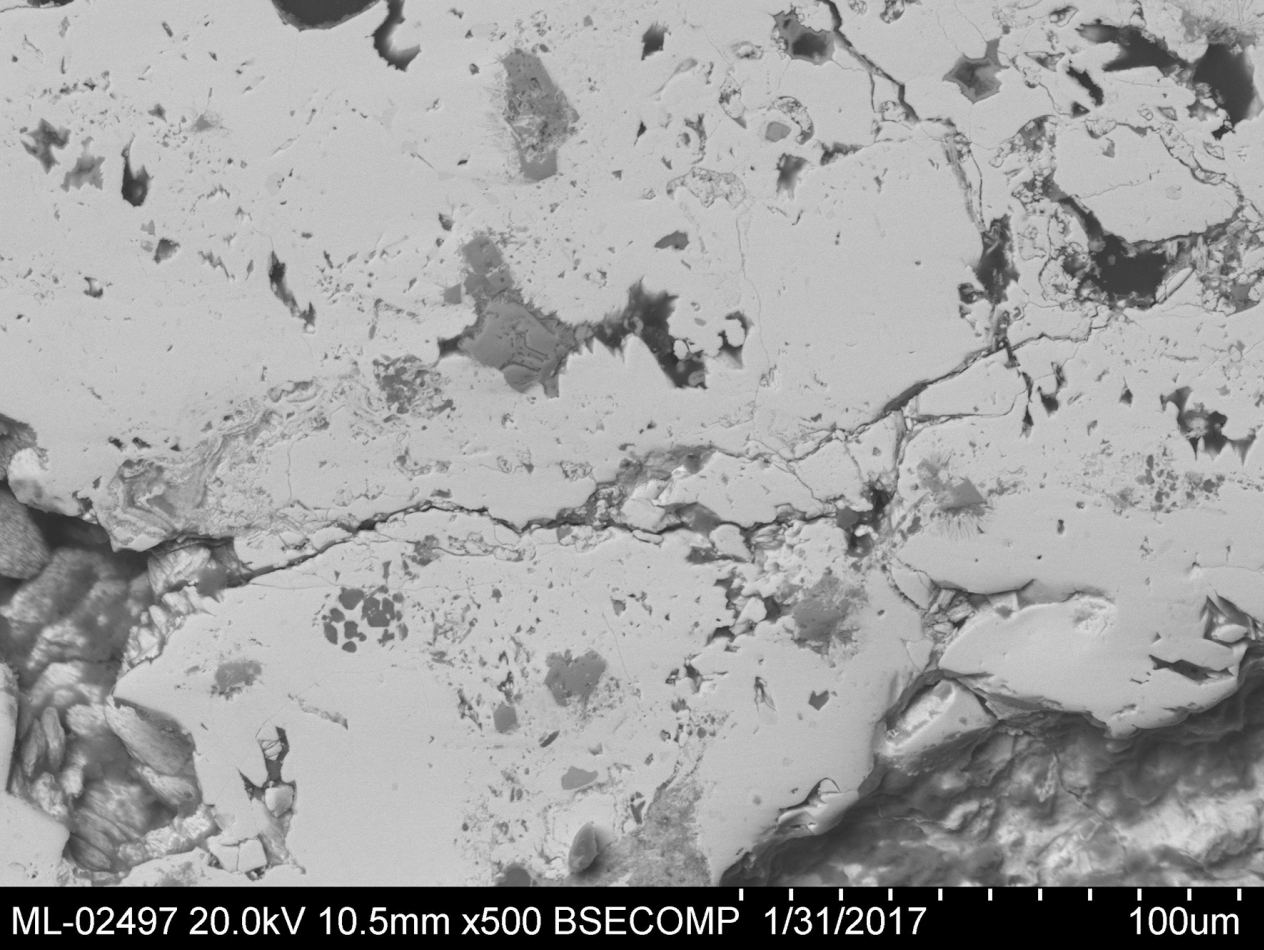
Detail of the lead oxide layer (white) showing the grey inclusions of complex oxides containing Fe, Zn, Pb and Cu. Blejeşti 1–1 sample. SEM image, backscattered electrons (S. Rovira).

**Fig 21:**
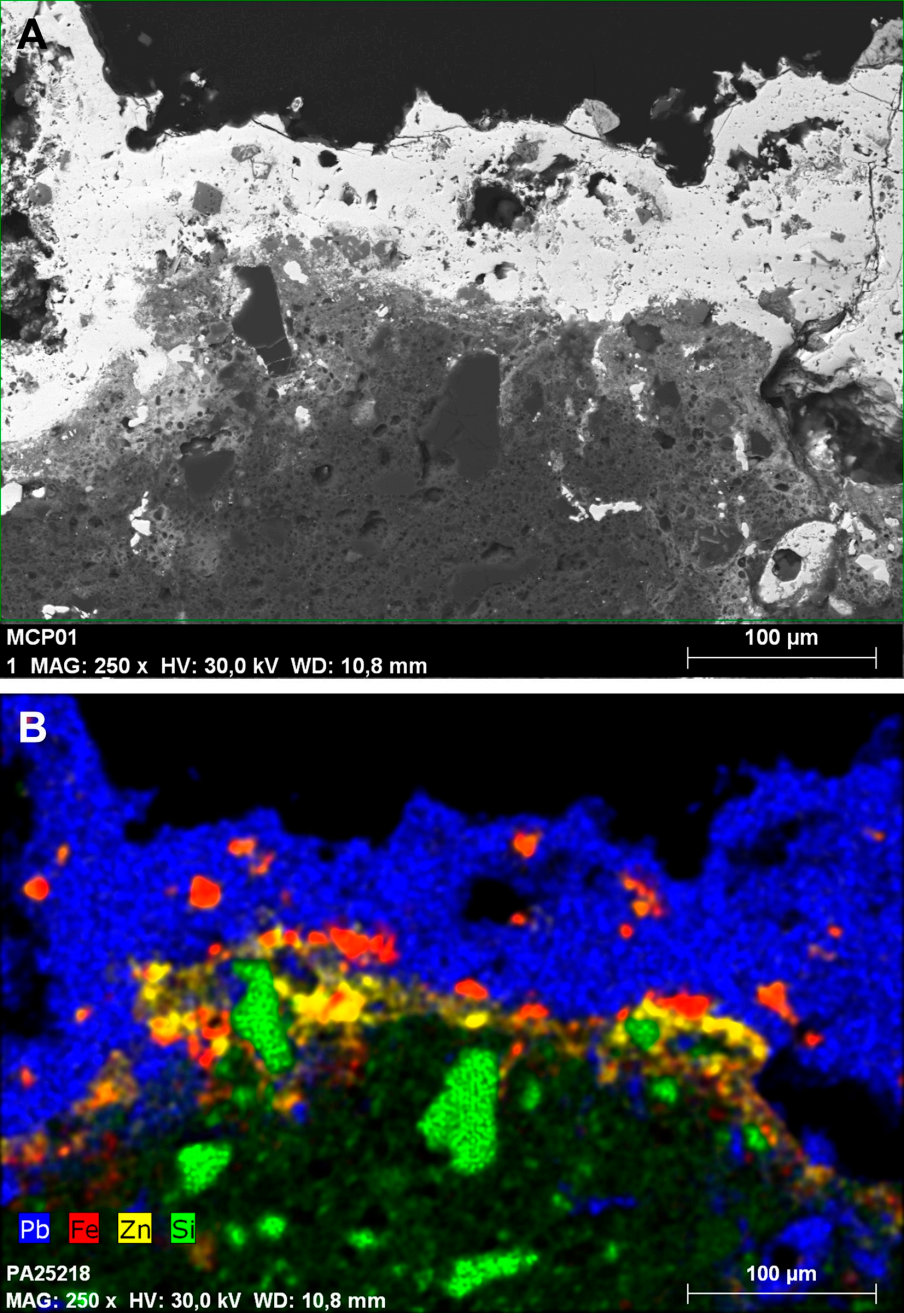
Section of the Blejeşti 1–1 sample (A) and distribution map of the elements Pb, Fe, Zn and Si (B). SEM image, backscattered electrons (S. Rovira).

The lead oxide layer also contains numerous grey inclusions, which appear to be composed of complex oxides in which iron predominates along with zinc, lead and copper (Blejeşti 1.1/02, /4, /05 and /06 in Table 3, Fig 20). These ferruginous inclusions are preferably lodged in the litharge layer and in the contact zone between the litharge and the ceramic (Fig 21). These such oxides clearly come from the original mineral load.

The ceramic fabric was made of clay with abundant quartz grains whose slightly rounded edges suggest that they are a natural temper contained in clay of alluvial origin. If they were ground quartz, the edges of the grains would be sharper. The fabric’s composition–determined in the SEM by scanning a window at 100X –is that of clay with a high content of aluminium oxide (Blejeşti 1-1/12). However, the SiO_2_/Al_2_O_3_ ratio (43.0/36.7) seems to be too different from what is customary in prehistoric ceramics, in which the aluminium oxide content is much lower [31]. At first glance it seems that clay with good refractory properties was selected for the elaboration of this crucible. When we have more analysis of the fabric of other similar crucibles we can assess whether we are facing a singular case or a trend with technological significance.

**Fig 22:**
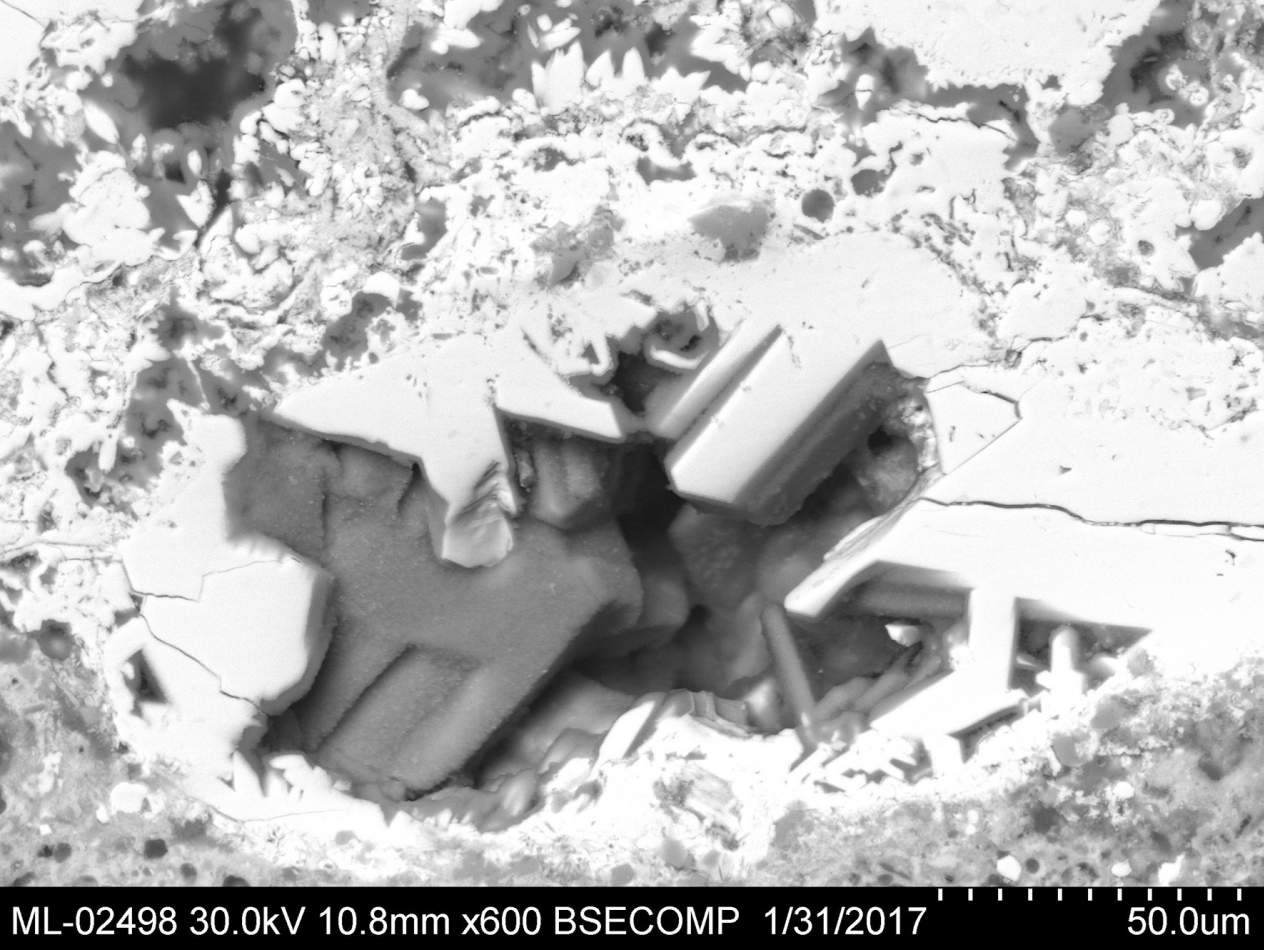
Secondary crystalline lead oxide within a microscopic cavity in the Blejeşti 1–1 sample. SEM image, backscattered electrons (S. Rovira).

Some cavities of the most glazed surface layer are filled by crystalline litharge formations, which suggest that there was a post-depositional oxidative process that in turn generated some micro-geodes (Fig 22).

## Interpretation of the analytical data

The galena relicts found in the leaded lump suggest that this is the starting mineral. From this, we will draw some hypotheses about the chemical processes that led to the production of the lump.

The galena powder introduced into the vessel through its small hole would react under oxidising conditions to become anglesite (lead sulphate, PbSO_4_). Lead sulphate is stable at temperatures below 1,000° C. If the temperature is higher, it decomposes into lead oxide (PbO, litharge) and sulphur trioxide (SO_3_, gas) that escapes into the atmosphere through the hole.

Palmierite is another major component of the lump (up to 9%). Natural palmierite [(K,Na)_2_Pb(SO_4_)_2_] is a rare and scarce mineral associated with volcanic fumaroles [32], but the variety in which sodium is replaced by potassium [K_2_Pb(SO_4_)_2_], artificially formed, is found in slags related to metallurgy of copper, silver and zinc [33–34]. To this species belongs the palmierite found in the lump Blejeşti 1–2, as sodium has not been detected in the analyses. To obtain palmierite it is necessary to introduce potassium into the system; that is to say, a compound containing potassium must first be added to the galena powder.

The most likely source of potassium during the Neolithic could have been potash (potassium carbonate K_2_CO_3_), gained by leaching ash from burning the wood of leafy trees. Wood ash has a variable composition, but in general the most abundant elements are calcium, potassium and magnesium, in descending order. With regard to this, pine ash has around 16% K, aspen 11% K, poplar 9% K and oak 6–10% K. The calcium content is between 20 and 40% [35]. As the lump does not contain calcium (see Table 2), it means that ash was previously treated to remove this element. That measure is quite simple, because potash is highly soluble in water, whereas calcium carbonate is far less soluble. Therefore, by treating the ash with abundant water (leaching) in a large vessel, the potash dissolves while the insoluble components precipitate as solids at the bottom. The water with dissolved potash is then removed and boiled until all the water evaporates, leaving a solid white residue–i.e., potash.

Unfortunately, we do not know of any evidence for the use of potash until historical times, when it was used in the soap-making industry. In any case, it does not appear that we can trace back the saponification of fats with potash beyond 2500 BCE in Sumerian times [36]. This is a severe setback for the hypothesis we are working on, although in the end we will see that there is another option to explain the presence of K_2_O in the system.

In order to understand how palmierite could be formed inside the vessel, we must accept that a very complex system of chemical reactions took place simultaneously. As a mere assumption, at a temperature slightly above 1,000° C, a part of the anglesite (PbSO_4_) decomposes into lead oxide releasing sulphur trioxide. At the same time, potash is decomposed by the thermal effect on potassium, oxygen and carbon dioxide (gas). The affinity of potassium for sulphur is large, so it must have reacted immediately with sulphur trioxide and oxygen to form potassium sulphate (K_2_SO_4_), which, in turn, joined the anglesite already present in the system to form palmierite [K_2_Pb (SO_4_)_2_]. Given the concentrations of anglesite and palmierite (see Fig 13), only a small part of anglesite had to decompose (meaning that the temperature exceeded 1,000° C a short time). Consequently, the amount of potash needed was also small and a little lead oxide (litharge) was retained in the lump.

This hypothesis, however, has several weaknesses, one of which is that the substance obtained must be in a liquid state to be able to evacuate it from the vessel without breaking it, and the anglesite has a melting point of 1,170° C. At that temperature, the anglesite is unstable and decomposes. In addition, we have already said that the time at which the temperature exceeded 1,000° C was likely short, too short to melt a large anglesite mass.

**Fig 23:**
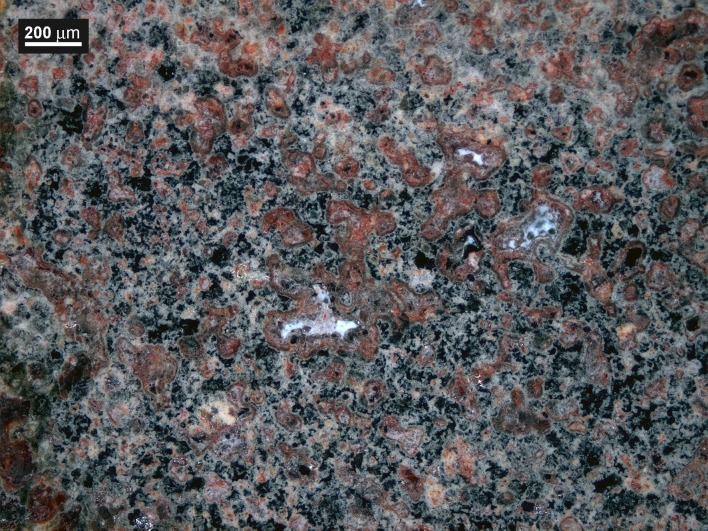
Appearance of the lump Blejeşti 1–2 core showing the colour diversity of its phases. The anglesite appears black, reddish, white and transparent crystalline. Palmierite is dispersed in clear crystalline form. OM image, Dark Field (S. Rovira).

Another weak point is to decide what use these substances might have had during the Neolithic. Although in their pure state they are colourless crystalline substances, the mineral catalogues report colours ranging from white to black through yellow, brown, red and grey shades for anglesite; palmierite is colourless, of crystalline texture. An image of the lump nucleus seen in the optical microscope with reflected light shows white, reddish, colourless and black anglesite together with colourless crystalline palmierite (Fig 23). Was it a pigment or a paint? After grinding the material, the resulting colour should be blackish grey with brown shades.

In the present day, the main industrial use of pure lead sulphate (crystalline white) is in the chemical industry as an intermediate reaction product to obtain other substances. It is also used, although in much smaller quantities, in the production of certain paints and varnishes, more as a flux than as a dye. It is a highly toxic product when in contact with skin, so its use as a cosmetic is unlikely [37]. Also, its usefulness as a colorant is doubtful. However, anglesite has been identified in three Egyptian cosmetics dated between 2000 and 1200 BCE. The main component shared by all is galena powder, while the concentration of anglesite oscillates between 4.1% and 13.3%, which makes them less toxic. Another cosmetic contains 8.4% palmierite [38]. The aforementioned work only notes the analytical results without going into the details of manufacturing. In any case, the composition of these Egyptian cosmetics has little to do with that of the Blejeşti lump, although in both cases the starting mineral is galena. Nevertheless, it must be noted that galena ore is frequently found in Predynastic Egypt from the Naqada IIC period onwards (second half of the 4^th^ millennium BCE) and is traditionally interpreted as “eye make-up” [39–40].

At this point, and without any other contemporary archaeological evidence upon which we can rely, another hypothesis arises: the leaded lump was not the desired end product, but instead a by-product of unfinished chemical reactions. The desired product may well have been another colouring material: the yellow or red lead oxide (litharge, PbO).

As mentioned above, at temperatures over 1,000° C the anglesite is decomposed into litharge and SO_3_. Litharge is liquid at the formation temperature (melting point 888° C) and can be easily evacuated from inside the vessel through the hole.

From the point of view of chemical reactivity, lead oxide is obtained directly by heating galena to 1,000° C without going through the intermediate state of anglesite. But if some anglesite is formed (which obviously happened in this case) and the temperature of the system does not reach the melting point of this such mineral (1,170° C), it will remain in a solid state inside the vessel after pouring off the liquid litharge. This intermediate product is not necessarily disposable. It can be recycled and used in further processes.

Some analytical data support this hypothesis that we refer to. First, the inner surface of the ceramic exhibits a continuous litharge coating layer (see Fig 21) with no inclusions other than those of Fe, Pb, Zn and Cu complex oxides from galena impurities. In this figure it is also observed that some grains of silica (green in Fig 21) penetrate the litharge layer, which suggests that the part of the ceramic in contact with the filler reacts to form the silicates with lead of the crust (Blejeşti 2–1 / 11 in Table 3). In this reaction K_2_O is released, since the amount of potassium in the bloated zone (2.1% K_2_O) is significantly lower than in the intact ceramic paste (4.2% K_2_O). This difference could justify the formation of the small amount of palmierite in the lump without resorting to the addition of potash.

## Conclusions from the laboratory analysis

Although these curious closed vessels with a single perforation on top were known for many years alongside the hypothesis according to which they could be used to make a dye or a paint, for the first time we have specific analytical data that allow us to better approximate the thermochemical mechanisms that justify this hypothesis. There still remains doubt about which colorant would be obtained from galena (anglesite or litharge?), as the data suggest both options. Yet it is also true that the probable lack of control over the chemistry of the process with such a precarious (but innovative) technology as that proposed by the Blejeşti and Pietrele vessels could lead to materials such as those studied here, which might be masking the reality we barely glimpse. Both the laboratory study of a larger number of samples as well as the systematic analysis of paints in archaeological objects may help in the future to better configure an explanatory hypothesis.

The technology put into practice is undoubtedly very novel and involves a high degree of empirical knowledge of the properties of a particular metal sulphide (galena) and the by-products of its decomposition by thermal effect in a controlled atmosphere such as the one provided by an almost closed chamber. The necessary oxidising environment could be achieved by introducing air through the opening using blowing pipes. The critical points of the chemistry related to the working temperature could be learned with practice, as the temperature is closely related to the colour of the hot body. Thus, the light red colour indicates approximately 870° C, light orange 980° C, yellow 1,050° C, light yellow 1,100° C and white 1,200° C. After having experienced what was happening inside the vessel, the craftsperson must have realized that by heating the ceramic long enough to a colour between light red and light orange, he could achieve the desired results, if his intention were to obtain litharge.

## Comparing laboratory analysis with portable XRF

A description of the p-XRF method and its limitaions is given in: (S1 Supporting Information). When comparing the results obtained by SEM and p-XRF on a freshly cut surface of the Blejeşti slag-like material (Table 4), the data correspond quite well. Slight deviations are visible, which are normal among different analytical methods. All values range in the same order of magnitude or lie close by each other. Additionally, the surface measurements taken by p-XRF do not differ significantly from the fresh surfaces. This means that we can use the p-XRF for a first analysis of similar objects that are not yet studied in laboratories (see below).

**Table 4:** Comparison of SEM and pXRF data for the lump Blejeşti 1–2.

Method	Pb	Cu	S	K	Al	P	Fe
SEM	62.5	1.1	9.3	2.8	1.3	0.4	1.1
pXRF fresh cut	45	0.48	8.4	3.9	0.6	0.7	1
pXRF surface	48	3.9	6.7	6.2	1.2	0.6	2.1

Mean values in wt% (for discussion see text).

The SEM of the melted ceramic surface were taken on clean and cut samples from inside the vessel, while p-XRF measurements were made inside and on the outer surface of the vessel, but not on clean or cut surfaces (Table 5). The p-XRF measurements of the inner surface correspond well with the SEM; only sulphur differs, for which a high relative bias is known for p-XRF (see: S1 Supporting Information). There is a strong difference in the sulphur content, though the potassium and calcium are roughly comparable, as is iron. It is important to note that the considerable variability in iron (and most likely calcium) attested by SEM differs from the mean values given by XRF, because here a bulk of an 8-mm diameter field of the surface is measured, while the SEM gives values for distinct points, thus showing greater variance. Also, a certain effect of deeper layers with differing composition is reflected in the p-XRF results. The inner and outer surfaces do not differ strongly, which confirms the expectation that the outer slaggy areas are most probably slag that was poured out from inside. However, we must take into account the inhomogeneity of the material itself and possible contaminations on the outside measurements from the adhesion of soil and probably alteration processes when we compare SEM and p-XRF. Nevertheless, the p-XRF analyses provide us with a useful set of data concerning the composition of the glassy molten ceramic layer, compared to SEM.

**Table 5:** Comparison of SEM and pXRF data for the glassy slaggy layer on the inner surface of the ceramic vessel Blejeşti 1–1 and pXRF data for slaggy patches on the outer surface of the vessel.

Method	S	K	Ca	Fe	Cu	Zn	Pb
SEM	0	0–1.8 (very rare)	0–0.9	0–59	+	+	+
pXRF inside	2.6	0.2	12.2	3	0.4	0.7	73.8
pXRF outside	3.7	0.7	0.8	4.9	1.5	0.8	20

Mean values and ranges in wt%, traces are marked with: + (for discussion see text).

The oxidic main components of the vessels clay show roughly similar values when p-XRF is compared to SEM (Table 6). It should be noted that the SEM was made using a clean-cut section of the sample, while the p-XRF was done on an outer surface of the vessel, upon which a certain amount of contamination by sediment and gypsum crust from the site may have remained. These are most probably responsible for the higher amounts of Ca, S, and P as well as the lower amount of Al alongside the deviations inherent in p-XRF. The difference in Al_2_O_3_ may be due to surface alteration and partially due to a bad resolution/high bias for Al_2_O_3_ of the pXRF device, since Al is one of the lightest elements that can be measured (see: S1 Supporting Information).

**Table 6:** Comparison of SEM and pXRF data for the clay composition of the vessel Blejeşti 1–1.

Method	SiO_2_	TiO_2_	Al_2_O_3_	FeO; Fe_2_O_3_	MnO	MgO	CaO	K_2_O	P_2_O_4_	SO_3_
SEM	43	1.4	36.7	9.6	-	2.1	2.5	4.2	0	0
pXRF surface	53	0.9	11.6	10.3	0.08	2.0	12.2	5.9	1.4	2.3

Mean values in wt%, normalized, MnO is not measured with SEM, FeO is due to SEM, Fe2O2 by pXRF (for discussion see text).

According to the analysis of different parts of the vessel and the leaded lump from Blejeşti, it is evident that the p-XRF provides us with a relatively precise dataset that is comparable to–and verified by–SEM (and furthermore by XRD and optical microscopy, see above). Therefore, we can use the p-XRF as an initial screening to sort out vessels with low, high or without lead traces relatively quickly and non-destructively. The p-XRF can be seen as an extension of laboratory-based geochemistry, employable on-site with the finds. It cannot and should not replace laboratory analysis, but it nevertheless presents a viable option when dealing with a large amount of finds from different sites and museums. Using this method, vessels with clear positive values can subsequently be chosen for further time- and cost-intensive laboratory analysis.

## Provenance by Lead Isotope Analysis

A sample from Blejeşti (1–2) and another from Pietrele (P06F3741096) were selected for Lead Isotope Analysis (LIA) and sent to the Geochronology and Geochemistry SGIker-Facility at the University of the Basque Country UPV/EHU (Spain). Pb isotopic ratios were measured on a high-resolution multicollector ICP-MS instrument (Neptune, Thermo Fisher Scientific) and the mass fractionation internally corrected after the addition of thallium isotopic reference material NBS-997. The detailed protocols were similar to those described by [41]. The accuracy of the results was confirmed by repeated analyses of lead isotopic reference material NBS-981.

**Fig 24:**
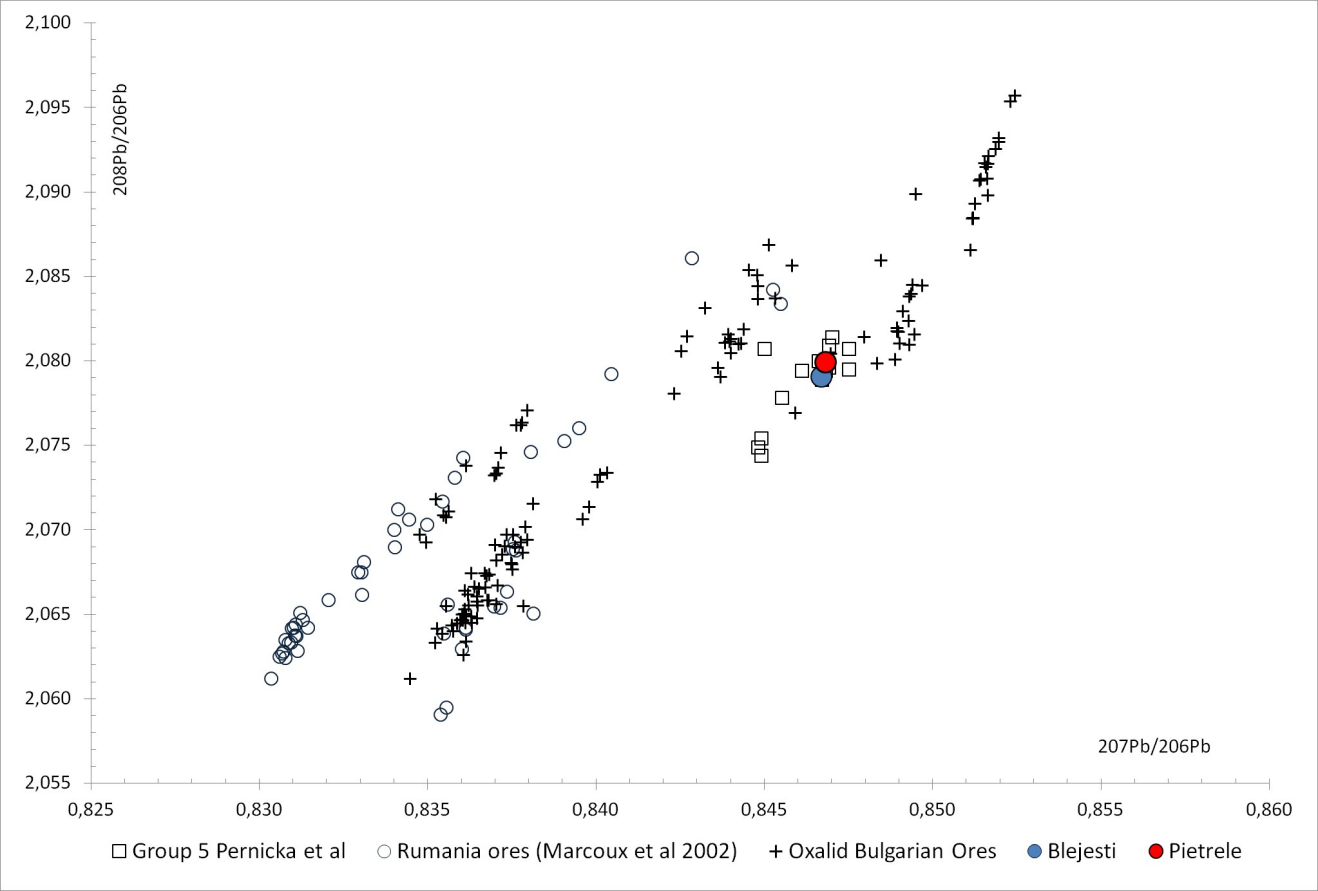
Lead isotope ratios of Blejeşti 1–2 and Pietrele P06F3741096 samples compared with Romanian ores by [42], Bulgarian ores by [44] and Bulgarian metal group 5 by [43] (I. Montero).

The results (Table 7) show that both samples probably have a similar provenance; however, there is no relationship with the ores from Romania [42] as is clearly stated in Fig 24.

**Table 7:** Lead isotope ratios in the Blejeşti 1–2 and Pietrele P06F3741096 samples analyzed by MC-ICP-MS.

Sample	^206^Pb/ ^204^Pb	2SE (abs)	^207^Pb/ ^204^Pb	2SE (abs)	^208^Pb/ ^204^Pb	2SE (abs)	^208^Pb/ ^206^Pb	2SE (abs)	^207^Pb/ ^206^Pb	2SE (abs)
**Pietrele**	18.4857	0.0008	15.6536	0.0008	38.4493	0.0020	2.07995	0.00004	0.84679	0.00001
**Blejeşti**	18.4753	0.0008	15.6429	0.0008	38.4117	0.0021	2.07909	0.00004	0.84669	0.00001

The probable provenance could be found in the Bulgarian resources, but it is difficult to be more precise. The samples from Blejeşti and Pietrele match the distribution of the archaeological grouplet 5 from Bulgaria proposed by [43], mainly formed by Late Chalcolithic copper items from Durankulak and Ruse Tell. This grouplet 5 was linked to the Amov’s isotopic filed C with a geographic distribution covering ores from northwestern Bulgaria in the West Balkan metallogenic zone, but also the Strandzha ore district, located in the eastern part of the Srednogorje metallogenetic zone.

**Fig 25:**
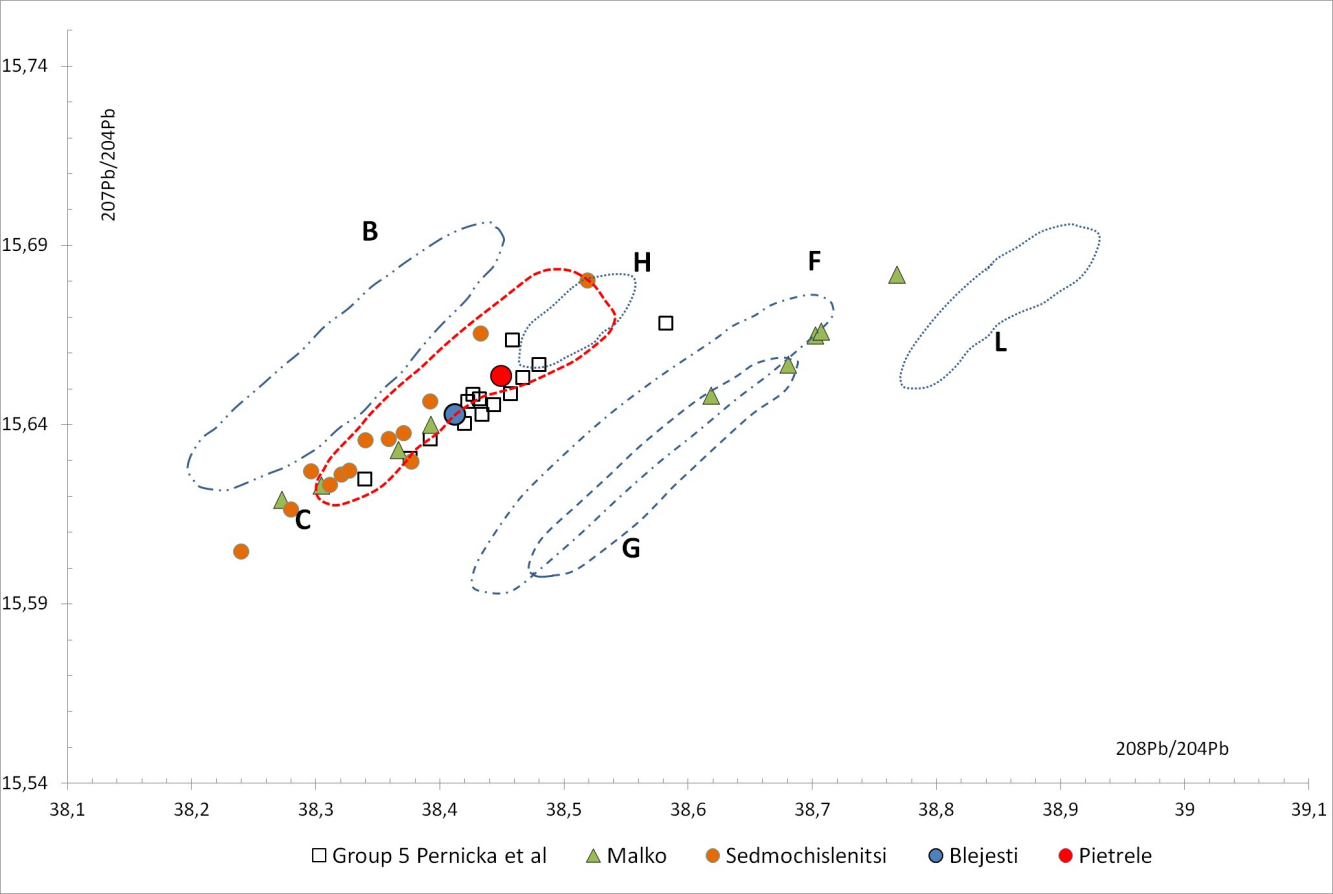
Lead isotope ratios of Blejeşti 1–2 and Pietrele P06F3741096 samples compared with isotopic fields defined by [47], Bulgarian metal group 5 by [43] and ores from Sedmochislenitsi and Malko Turnovo deposits [44] (I. Montero).

The paper by [43] were mainly focused on copper because at that moment there was no reference to the use of lead at this period, so a broader perspective is achieved by managing the actual LIA dataset from [44] and not only previously published papers about Bulgarian metallurgy and ores [45–46]. In this comparison we must also mention the discrepancies in Bulgarian ore measurements between those of Oxford and Amov’s laboratory [45] and the absence of use of the standard NIST981 in the analysis made before 1983 by the latter that could explain some inconsistencies. In this case, the plot combining ^207^Pb/^206^Pb– ^208^Pb/^206^Pb ratios (Fig 24) shows very few ores that could match the Blejeşti and Pietrele samples and also the grouplet 5. These ores come from the Sedmochislenitsi Pb-Zn-Cu deposit in the northwest (Iskar Vratsa) region and from the Malko Turnovo area in the southeast, close to the Black Sea, where lead and lead-silver deposits are located. The LIA from Malko Turnovo, however, has two distribution areas (Fig 25): one inside the isotopic field C, and the other in the isotopic field G. The data from [47] only contains samples matching field G, but [45] published another 4 samples, 2 in each field. In fact, 3 of the 9 samples in the Oxalid database are described as galena, chalcopyrite and sphalerite ores, but only two of them matched the isotopic field C. This double isotopic distribution found in the ores from Malko Turnovo needs a geological explanation.

At this point, two options remain open regarding the provenance of the lead used in the Romanian sites of Blejeşti and Pietrele: the northwestern region of Bulgaria, closest to the Romanian border, or the Malko Turnovo mines, close to the Black Sea coast in the southeast of Bulgaria, both containing lead and copper ores also compatible with Chalcolithic copper items with similar isotopic ratios (grouplet 5).

## Conclusions

The biconical vessels have been previously interpreted as lamps, and still others–without clear traces of heat–as miniature vessels or rattles, and when taking into account our new results, we also struggle to find an appropriate designation. The term ‘crucible’ is traditionally used for open forms or bowl-shaped, concave vessels in archaeometallurgical contexts (sometimes also in the field of glass-making), but it seems obvious that we are dealing with an additional kind of closed crucible. For the Chalcolithic Southeastern Europe, these objects define a very real new category of artefacts belonging to a sphere in between traditional fields of definition. They are related to a wider metallurgical context, primarily due to the ore stemming from raw material sources that were used extensively for copper production at that time. But they reach far beyond what we have hitherto called metallurgy. The mysterious final product seems to no longer belong the category of simple metal tools or jewellery; it remains unclear as to what final product was gained by smelting galena ore in this way.

The number of these vessels in Pietrele, their use as grave goods here as well as in Vărăști, and their supposed occurrence in a number of other Copper Age settlements in Romania and Bulgaria show the significance of this phenomenon. It must have been a widespread and more or less well known practice and a significant aspect of cultural habit during a distinct period and in a distinct place, i.e. ca. 4400–4300 BCE in the Lower Danube region and very probably far beyond this region. Minerals and metal ores were more than just raw material to be transformed in a direct and straightforward causal way into more or less pure metal. They had become a resource for ongoing pyrotechnical experimentation that reached a high level. For the first time, experimentation with lead ore can be shown in a clear chronological horizon in 4400–4300 BCE in Southeastern Europe.

## Supporting information

S1 Table Standard reference materials used for data quality evaluation of the p-XRF measurements with indications of their composition, amount of volatile components (loss on ignition, LOI) and Na2O, if specified (data according to: CanmetMINING 1995; Potts 2015; Potts et al. 2003; Thermo Scientific 2013; Webb et al. 2015; Wise–Watters 2009, 2012). (PDF)

S2 Table Expected values, mean results of the p-XRF measurements, number of p-XRF measurements (n), standard deviation (1 σ SD), relative standard deviation (RSD), bias and relative bias for oxides (in wt%) and selected elements (in ppm) for certified reference materials (CRM data according to: CanmetMINING 1995; Potts 2015; Potts et al. 2003; Thermo Scientific 2013; Webb et al. 2015; Wise–Watters 2009, 2012). (PDF)

S3 Table Coefficient of correlation (r^2^) intercept and slope for oxides and elements by p-XRF. (PDF)

S1 Fig Coefficient of correlation (r^2^) intercept and slope according to biplots for the comparison of p-XRF and recommended values (CRM) for the oxides SiO2, TiO2, Al2O3, Fe2O3(T), MnO, MgO, K2O P2O5 and the elements S, Pb, Cu, Zn. (PDF)

S1 Supporting Information (DOCX)
