# Supplementary material for: The earliest lead ore processing in Europe. 5th millennium BC finds from Pietrele on the Lower Danube
Source: PLoS One. 2019 Apr 10;14(4):e0214218. doi: 10.1371/journal.pone.0214218 (PMC6457500; doi:10.1371/journal.pone.0214218)
Supplement: S1 Table — (PDF) [file pone.0214218.s001.pdf]

standard	composition	LOI	CO_2, Na, Na_2O
LOESS-1 Koeln	loess	16.3 wt%	CO_2 14.9 wt%, Na_2O 1.1 wt%
NIST 2709aPP 180-649	agricultural soil	not specified	Na 1.22 wt%,
NIST 2780	hard rock mining waste	not specified	Na 0.221 wt%,
TILL-4 180-646	till, blended with molybdenium rich soil	5.7 wt% (1000 °C)<br>4.4 wt% (500°C)	Na_2O 3.46 wt%
SAR-M 180-673	not specified	not specified	not specified
SdAR-M2	blended metal-rich sediment	1.6 wt%	Na_2O 2.58 wt%
SiO_2 180-647	not specified	<0.005 wt%	not specified
