# Supplementary material for: The earliest lead ore processing in Europe. 5th millennium BC finds from Pietrele on the Lower Danube
Source: PLoS One. 2019 Apr 10;14(4):e0214218. doi: 10.1371/journal.pone.0214218 (PMC6457500; doi:10.1371/journal.pone.0214218)
Supplement: S2 Table — (PDF) [file pone.0214218.s002.pdf]

oxide	refrence	n	pXRF mean	expected	SD	rel SD (%)	bias	rel bias (%)
SiO_2	LOESS-1 Koeln	84	61,91	53,24	0,57	0,92	8,67	16,29
SiO_2	NIST2709aPP	47	71,47	69,24	0,27	0,37	2,23	3,22
SiO_2	NIST2780	209	71,31	70,62	0,33	0,47	0,69	0,98
SiO_2	TILL-4	82	73,16	65,00	0,22	0,30	8,16	12,56
SiO_2	SAR-M 180-673	26	77,90	76,09	0,23	0,29	1,82	2,39
SiO_2	SdAR-M2	5	80,09	73,45	0,11	0,13	6,64	9,05
SiO_2	SiO2	40	99,99	99,99	0,01	0,01	0,00	0,00
TiO_2	LOESS-1 Koeln	84	0,48	0,42	0,02	4,40	0,06	14,32
TiO_2	NIST2709aPP	47	0,76	0,60	0,04	5,46	0,16	27,24
TiO_2	NIST2780	209	1,33	1,24	0,07	5,52	0,09	7,08
TiO_2	TILL-4	82	0,93	0,81	0,04	4,07	0,12	15,30
TiO_2	SAR-M 180-673	26	0,63	0,67	0,05	7,87	-0,04	-6,03
TiO_2	SdAR-M2	5	0,27	0,30	0,01	2,32	-0,03	-9,45
Al_2O_3	LOESS-1 Koeln	84	6,07	6,20	0,13	2,18	-0,13	-2,17
Al_2O_3	NIST2709aPP	47	12,58	14,87	0,42	3,31	-2,29	-15,41
Al_2O_3	NIST2780	209	15,87	17,85	0,70	4,39	-1,98	-11,08
Al_2O_3	TILL-4	82	12,34	14,40	0,38	3,09	-2,06	-14,32
Al_2O_3	SAR-M 180-673	26	10,20	12,53	0,62	6,10	-2,32	-18,53
Al_2O_3	SdAR-M2	5	9,58	12,47	0,25	2,63	-2,89	-23,15
Fe_2O_3	LOESS-1 Koeln	84	2,77	2,10	0,06	2,20	0,67	31,79
Fe_2O_3	NIST2709aPP	47	6,26	5,13	0,25	3,99	1,13	21,93
Fe_2O_3	NIST2780	209	4,69	4,24	0,25	5,41	0,45	10,72
Fe_2O_3	TILL-4	82	6,95	5,63	0,25	3,56	1,32	23,48
Fe_2O_3	SARM	26	4,88	4,50	0,31	6,44	0,38	8,52
Fe_2O_3	SdAR-M2	5	2,77	2,63	0,10	3,71	0,14	5,28
MnO	LOESS-1 Koeln	84	0,08	0,06	0,00	3,74	0,02	23,65
MnO	NIST2709aPP	47	0,08	0,07	0,00	5,22	0,01	13,03
MnO	NIST2780	209	0,08	0,06	0,01	6,96	0,02	24,92
MnO	TILL-4	82	0,08	0,06	0,00	4,43	0,02	25,27
MnO	SAR-M 180-673	26	0,61	0,71	0,04	6,92	-0,10	-14,66
MnO	SdAR-M2	5	0,12	0,13	0,00	2,61	-0,01	-8,15
MgO	LOESS-1 Koeln	84	1,65	2,90	0,35	21,32	-1,25	-43,12
MgO	NIST2709aPP	47	2,07	2,59	0,24	11,46	-0,52	-19,96
MgO	NIST2780	209	0,84	0,94	0,24	28,30	-0,10	-10,70
MgO	TILL-4	82	1,00	1,26	0,20	20,14	-0,26	-20,45
MgO	SAR-M 180-673	26	0,75	0,87	0,21	28,44	-0,12	-13,71
MgO	SdAR-M2	5	0,52	0,49	0,18	34,65	0,03	5,73
CaO	LOESS-1 Koeln	84	25,17	16,31	0,53	2,11	8,86	54,33
CaO	NIST2709aPP	47	3,59	2,85	0,13	3,63	0,74	25,88
CaO	NIST2780	209	0,33	0,29	0,02	5,79	0,04	14,30
CaO	TILL-4	82	1,43	1,25	0,06	4,22	0,18	14,36
CaO	SARM	26	0,86	0,90	0,07	7,88	-0,04	-4,47
CaO	SdAR-M2	5	0,88	0,84	0,03	3,20	0,04	4,55
K_2O	LOESS-1 Koeln	84	1,87	1,30	0,04	2,35	0,57	43,77
K_2O	NIST2709aPP	47	2,98	2,71	0,10	3,46	0,27	9,93
K_2O	NIST2780	209	5,35	4,34	0,25	4,65	1,02	23,50
K_2O	TILL-4	82	3,95	3,25	0,14	3,49	0,70	21,58
K_2O	SAR-M 180-673	26	3,95	3,73	0,21	5,34	0,22	6,00
K_2O	SdAR-M2	5	5,65	5,00	0,19	3,38	0,65	12,99
P_2O_5	LOESS-1 Koeln	84	0,00	0,13	0,00	0,00	-0,13	-100,00
P_2O_5	NIST2709aPP	47	0,20	0,17	0,01	5,10	0,03	17,42
P_2O_5	NIST2780	209	0,19	0,10	0,01	4,02	0,08	80,09
P_2O_5	TILL-4	82	0,15	0,20	0,01	7,47	-0,05	-23,09
P_2O_5	SAR-M 180-673	26	0,22	0,02	0,01	4,20	0,20	1184,48
P_2O_5	SdAR-M2	5	0,11	0,08	0,00	2,95	0,03	42,02

element	refrence	n	pXRF mean	expected	SD	RSD%	bias	rel bias (%)
S	NIST2780	209	5070	12630	279	6	-7.560,4	-59,9
S	TILL-4	82	385	800	30	8	-415,4	-51,9
S	SdAR-M2	5	487	970	8	2	-483,5	-49,8
Cu	LOESS-1 Koeln	84	15	11,31	3	21	3,8	33,7
Cu	NIST2709aPP	47	29	33,9	3	9	-4,6	-13,7
Cu	NIST2780	209	165	216	47	28	-51,2	-23,7
Cu	TILL-4	82	209	237	6	3	-28,2	-11,9
Cu	SAR-M 180-673	26	263	331	16	6	-68,3	-20,6
Cu	SdAR-M2	5	195	236	4	2	-41,0	-17,4
Zn	LOESS-1 Koeln	84	32	34,4	2	6	-2,9	-8,4
Zn	NIST2709aPP	47	86	103	3	3	-16,8	-16,3
Zn	NIST2780	209	1994	2570	114	6	-575,8	-22,4
Zn	TILL-4	82	82	70	3	4	11,7	16,7
Zn	SAR-M 180-673	26	765	930	33	4	-164,6	-17,7
Zn	SdAR-M2	5	665	760	10	2	-94,9	-12,5
Pb	LOESS-1 Koeln	84	11	11,34	1	8	0,0	0,4
Pb	NIST2709aPP	47	17	17,3	1	6	-0,8	-4,6
Pb	NIST2780	209	5049	5770	65	1	-721,3	-12,5
Pb	TILL-4	82	54	50	2	3	4,0	7,9
Pb	SAR-M 180-673	26	993	982	17	2	10,5	1,1
Pb	SdAR-M2	5	851	808	10	1	43,1	5,3
