# Supplementary material for: The earliest lead ore processing in Europe. 5th millennium BC finds from Pietrele on the Lower Danube
Source: PLoS One. 2019 Apr 10;14(4):e0214218. doi: 10.1371/journal.pone.0214218 (PMC6457500; doi:10.1371/journal.pone.0214218)
Supplement: S3 Table — (PDF) [file pone.0214218.s003.pdf]

oxide	r^2	intercept	slope
SiO_2	0,9533	17,483	0,8145
TiO_2	0,9580	0,0006	1,0917
Al_2O_3	0,9574	0,2277	0,8335
Fe_2O_3	0,9611	-0,1957	1,2173
MnO	0,9994	0,0235	0,8187
MgO	0,8966	0,2826	0,5677
CaO	0,9994	-0,494	1,5696
K_2O	0,9615	0,3222	1,0737
P_2O_5	0,0357	0,1716	-0,2312
element	r^2	intercept	slope
S	1,0000	86,201	0,3946
Cu	0,9917	4,8983	0,7945
Zn	0,9985	28,868	0,7724
Pb	0,9987	54,561	0,8702
