# Supplementary figures and images for: The earliest lead ore processing in Europe. 5th millennium BC finds from Pietrele on the Lower Danube
Source: PLoS One. 2019 Apr 10;14(4):e0214218. doi: 10.1371/journal.pone.0214218 (PMC6457500; doi:10.1371/journal.pone.0214218)

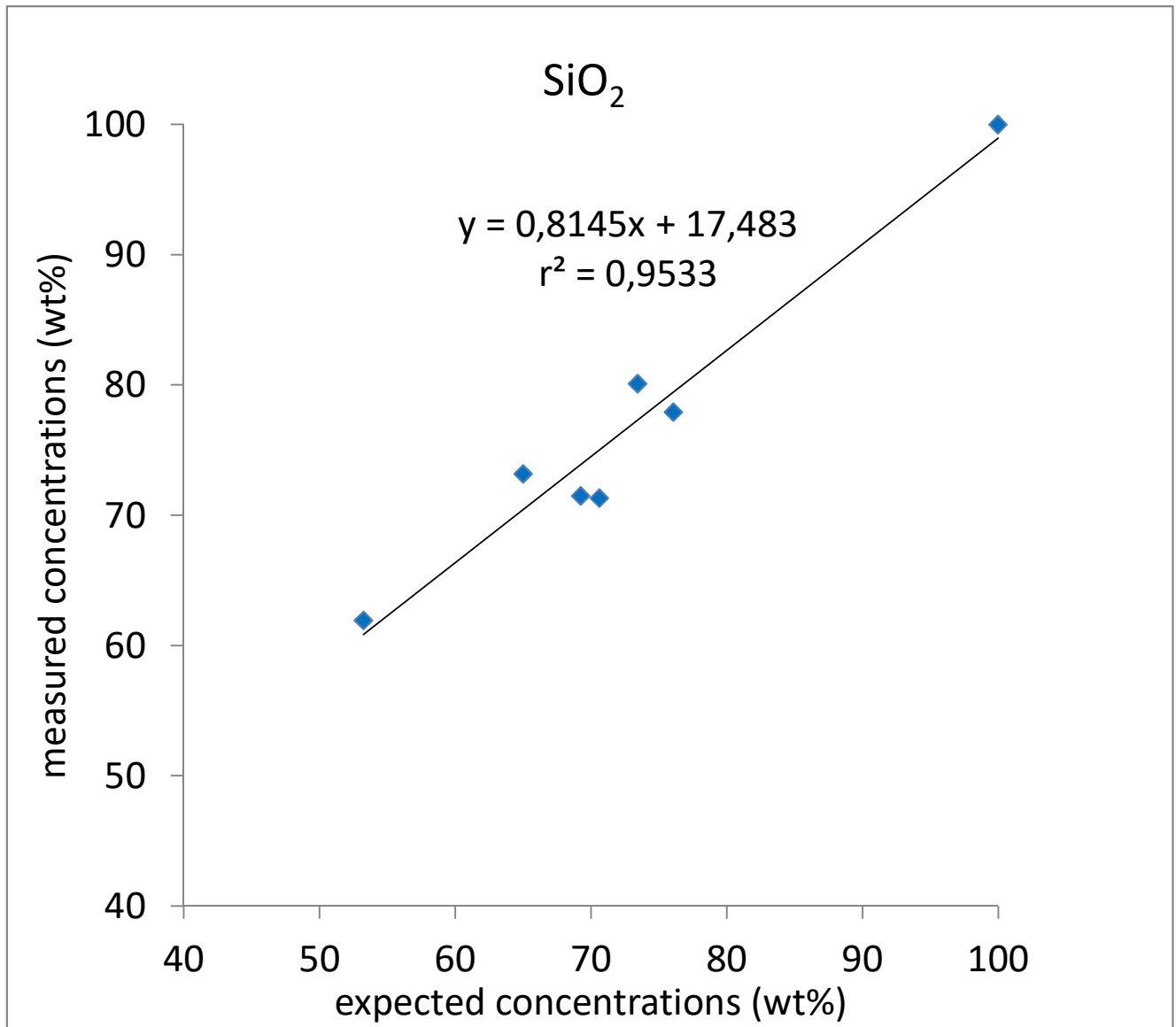

TiO<sub>2</sub>

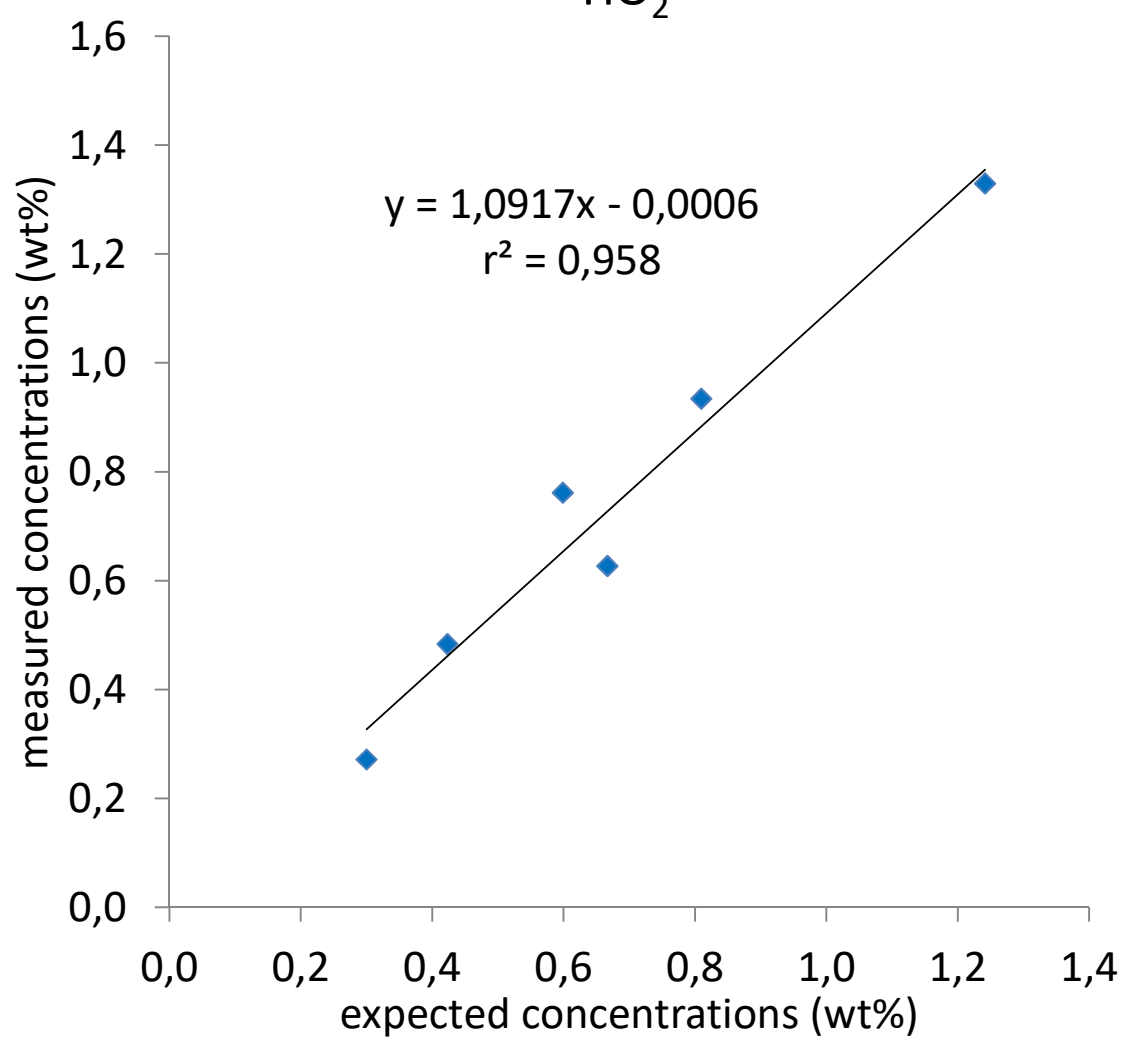

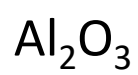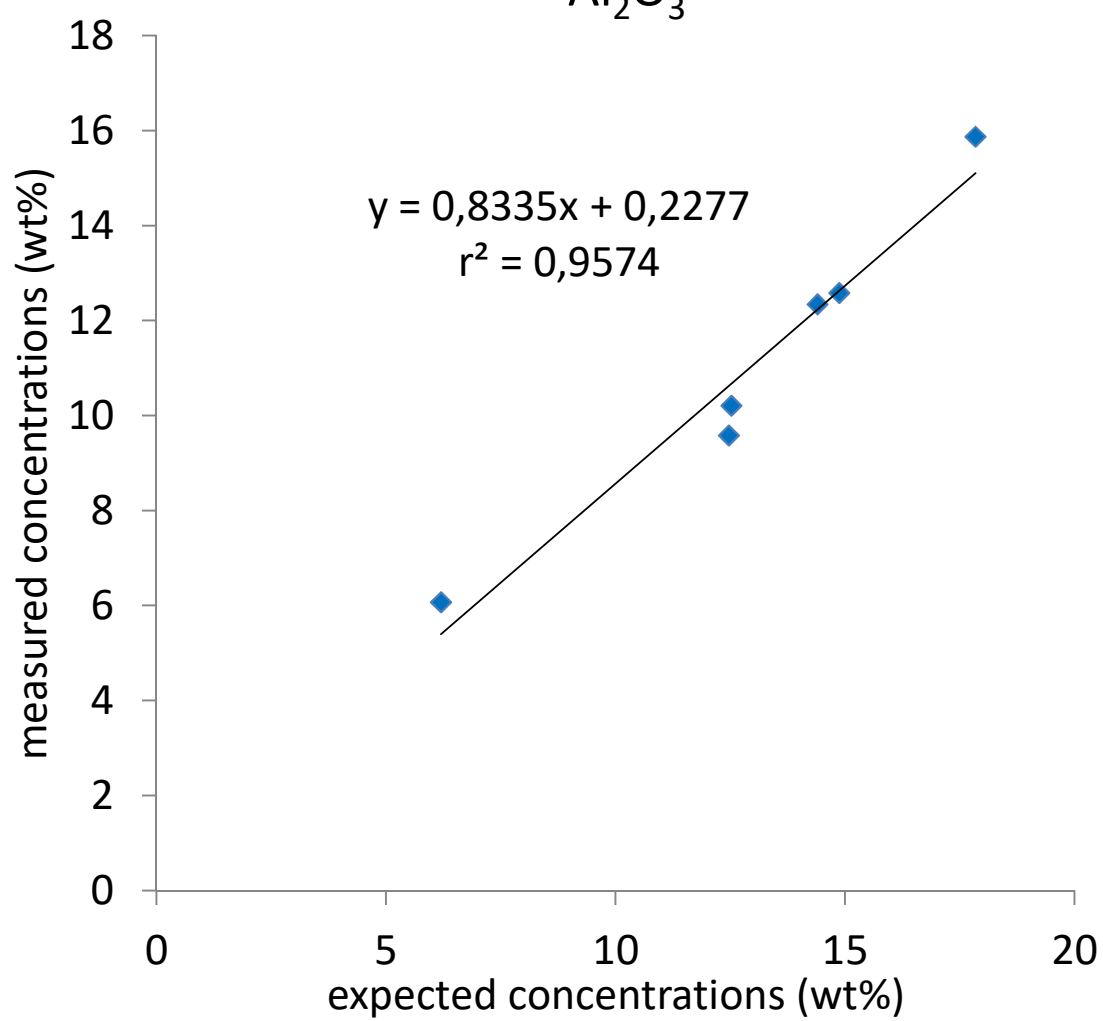

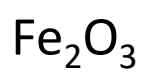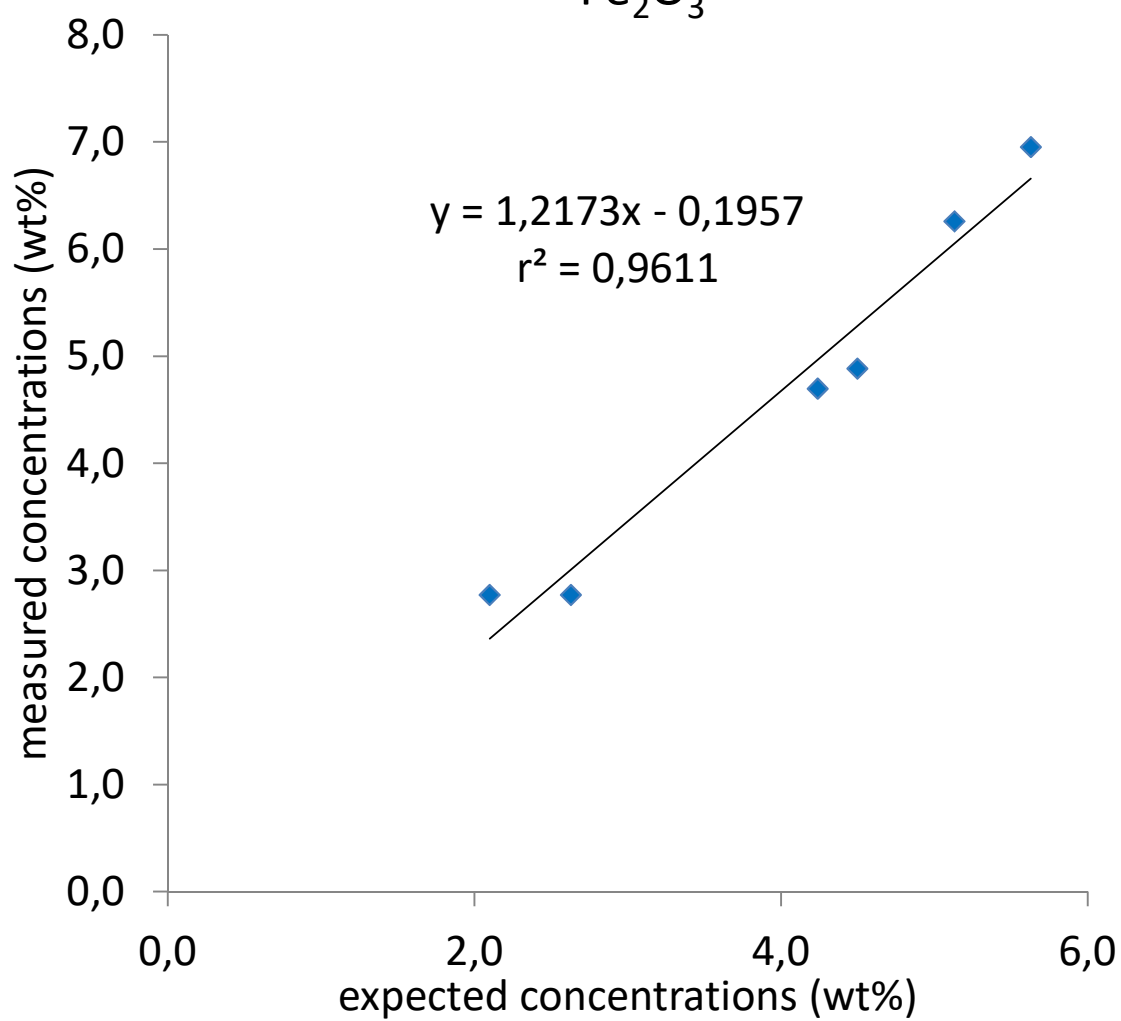

MnO

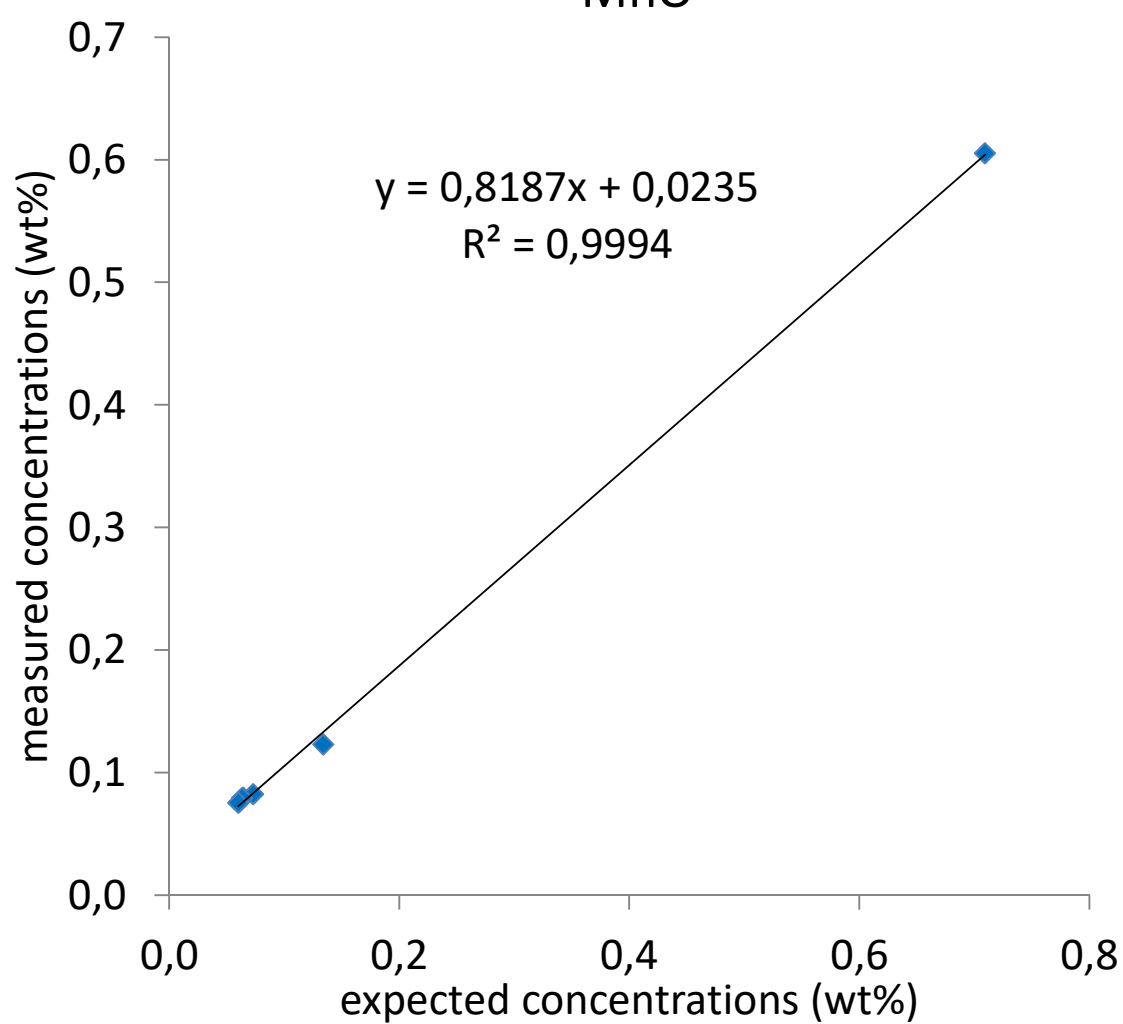

MgO

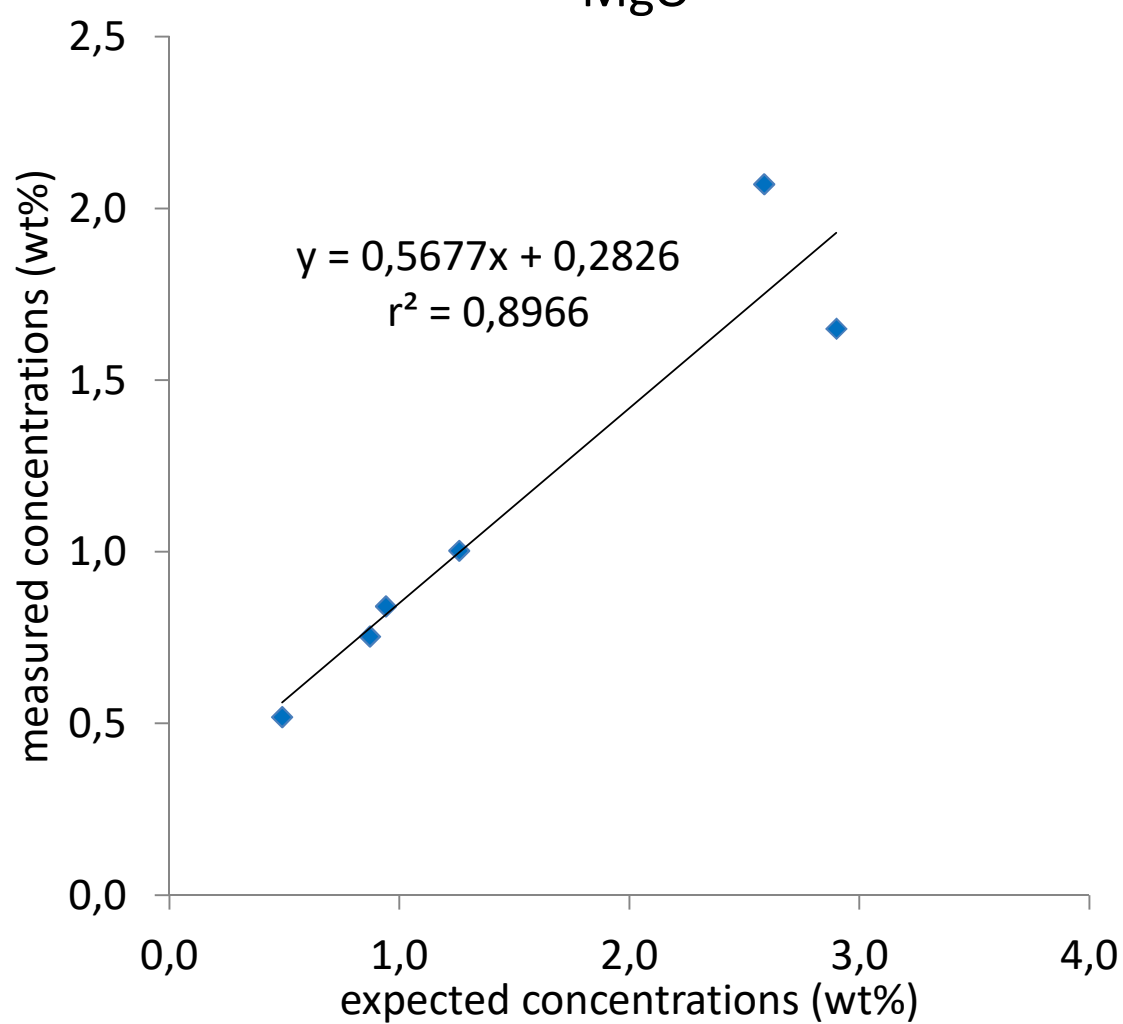

CaO

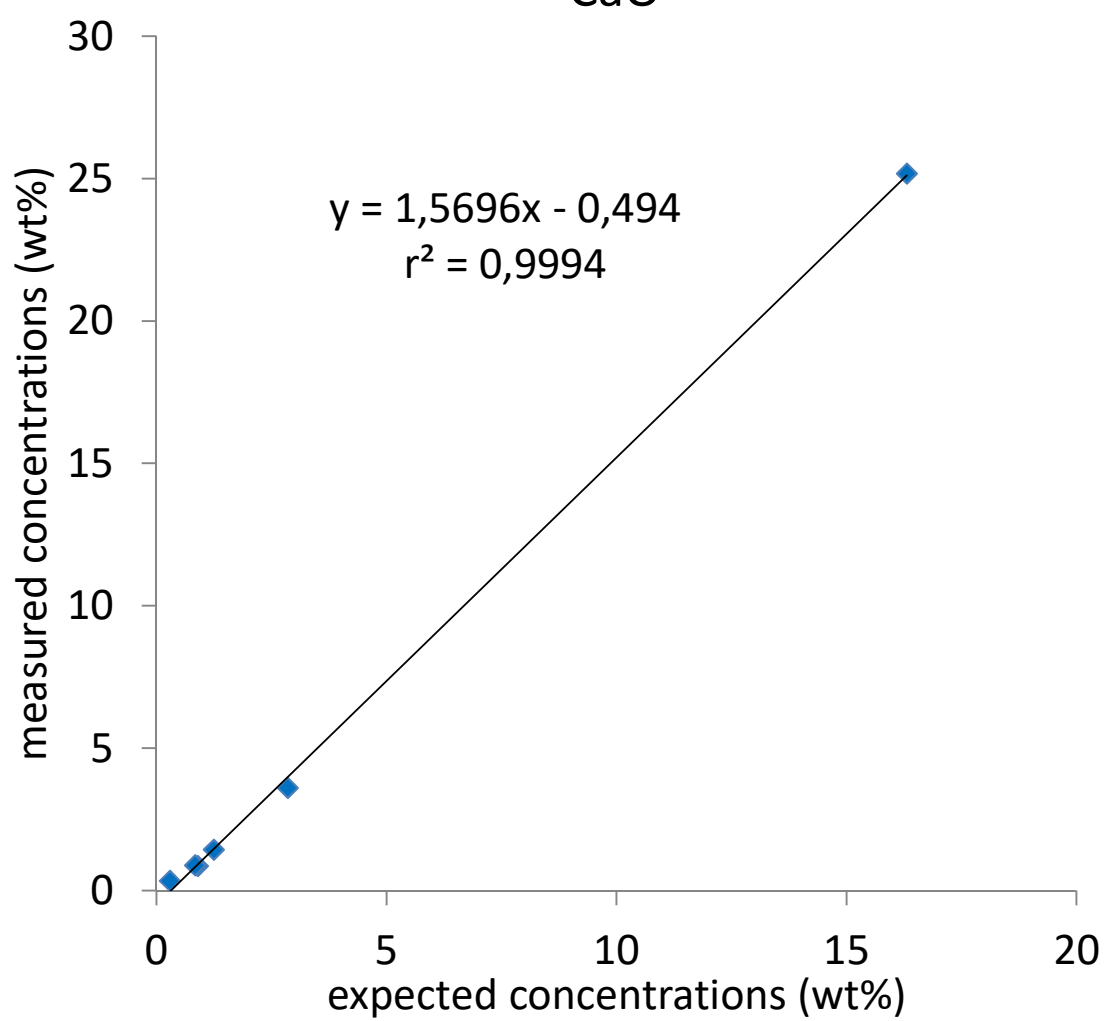

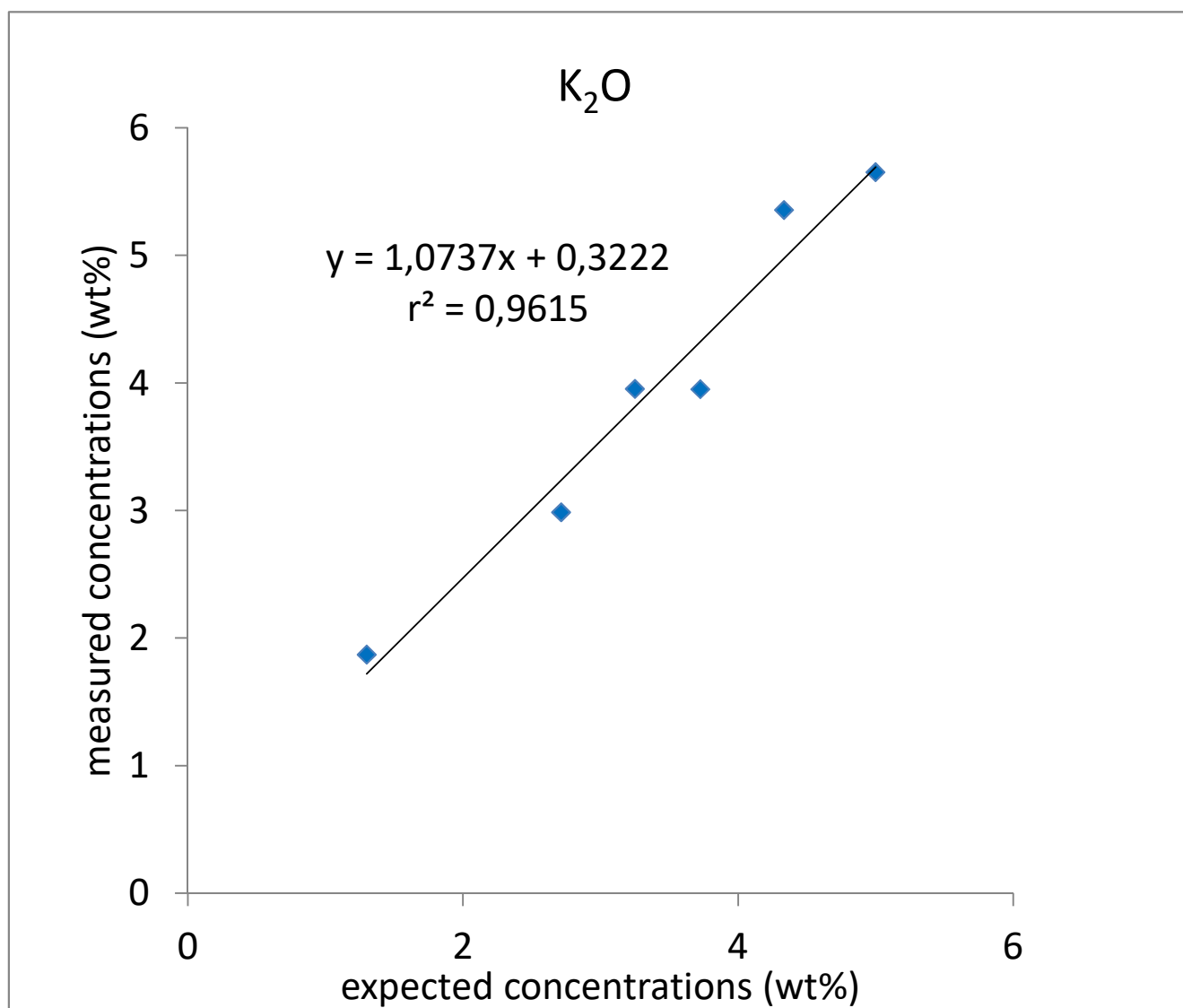

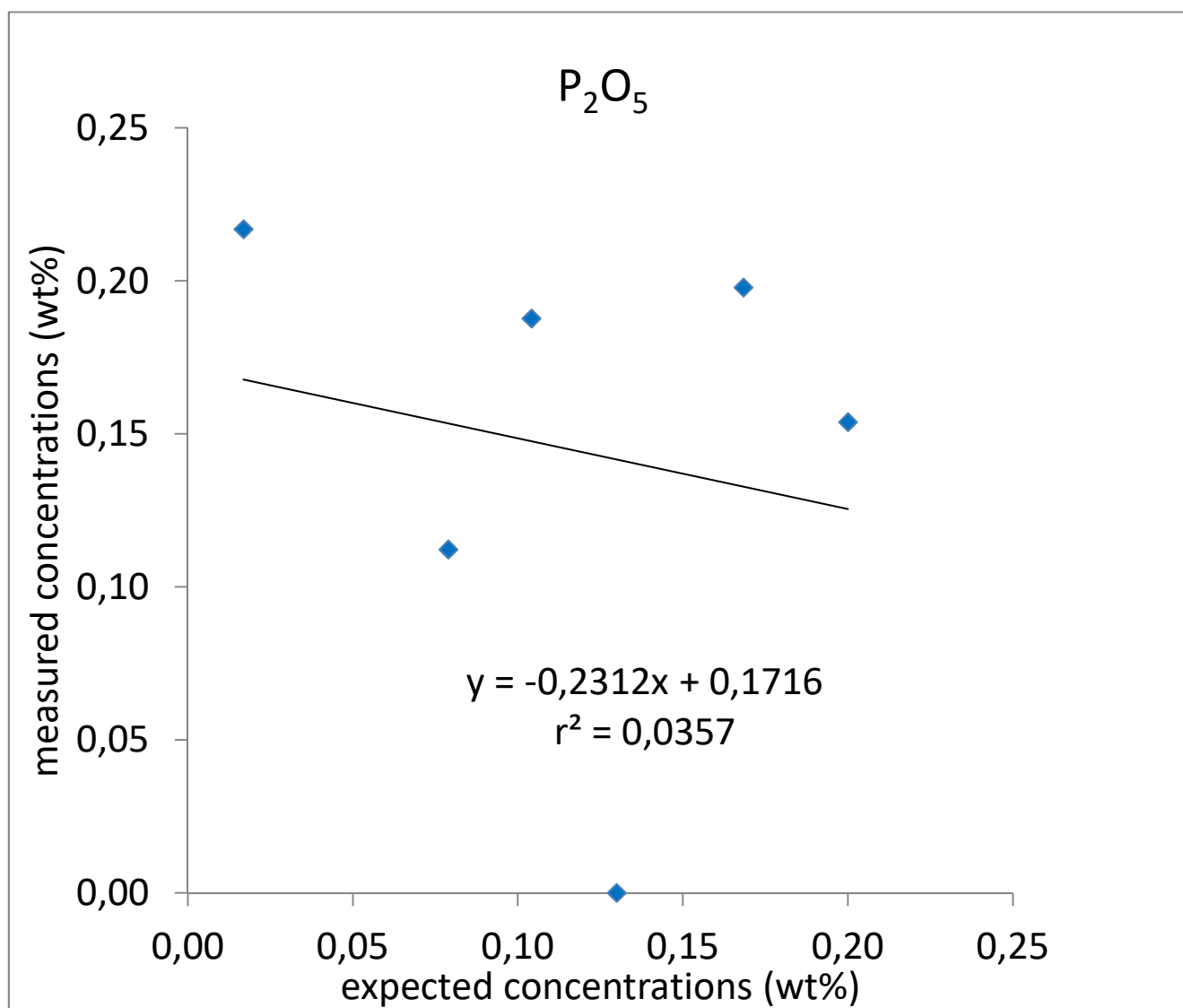

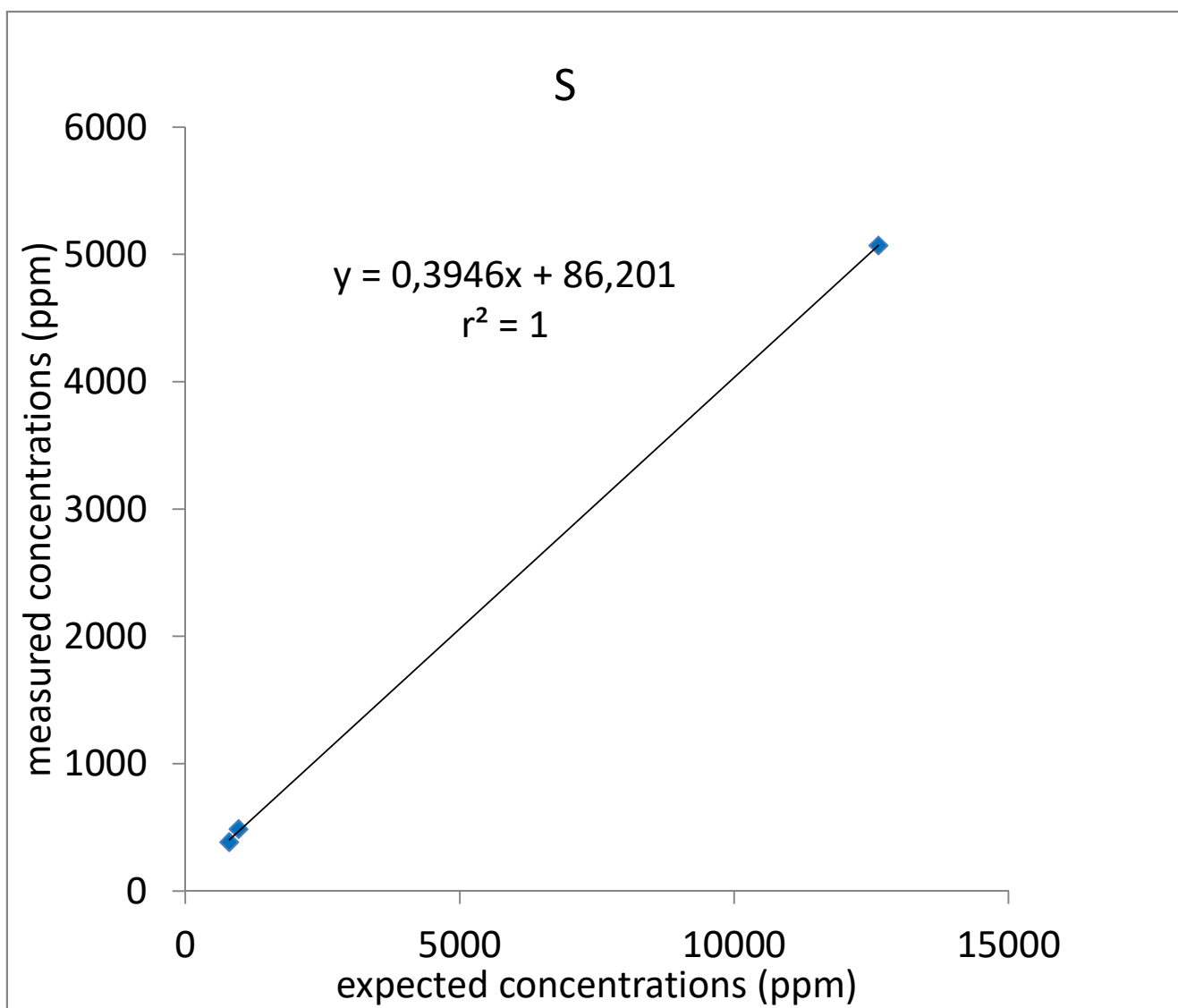

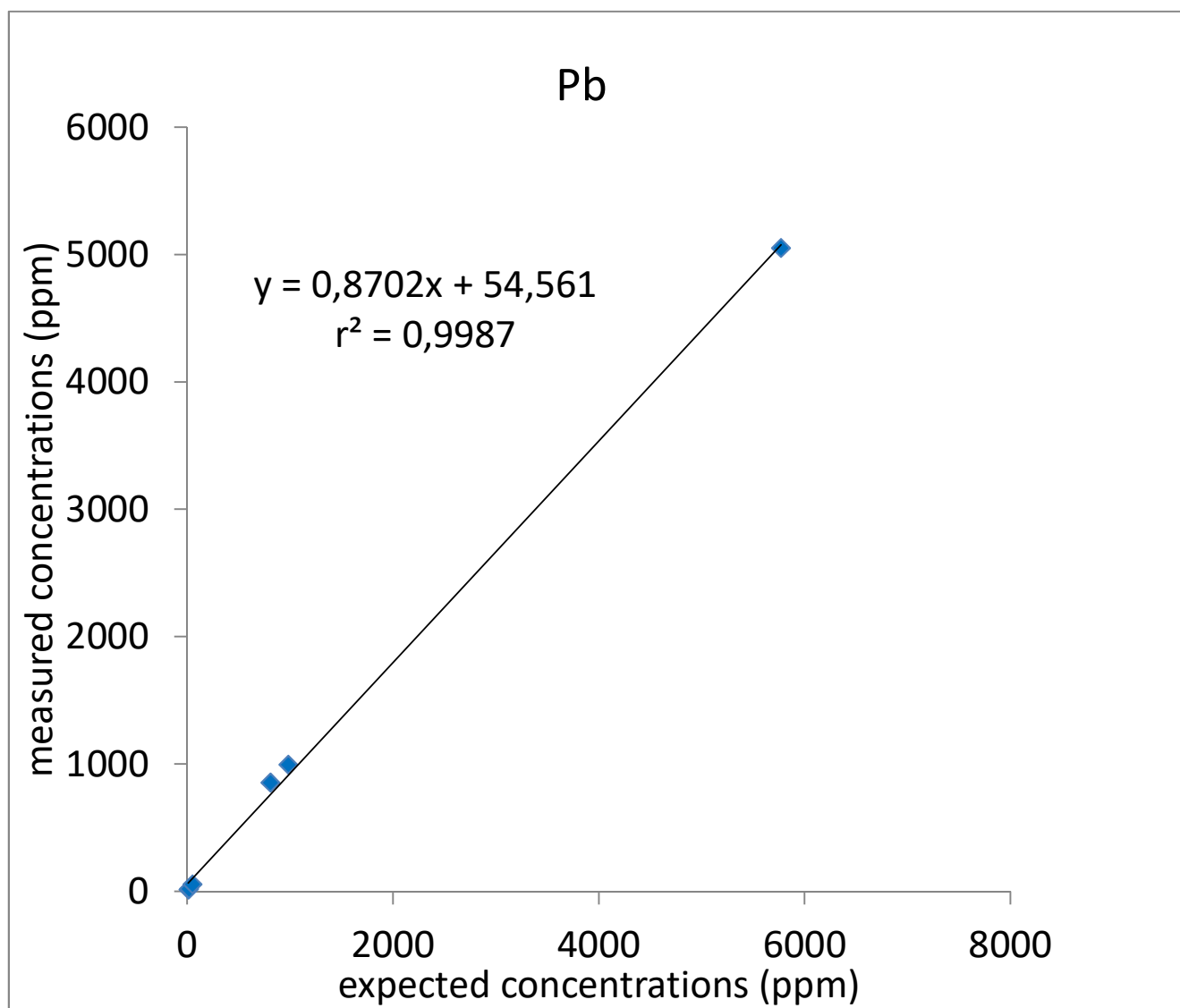

Cu

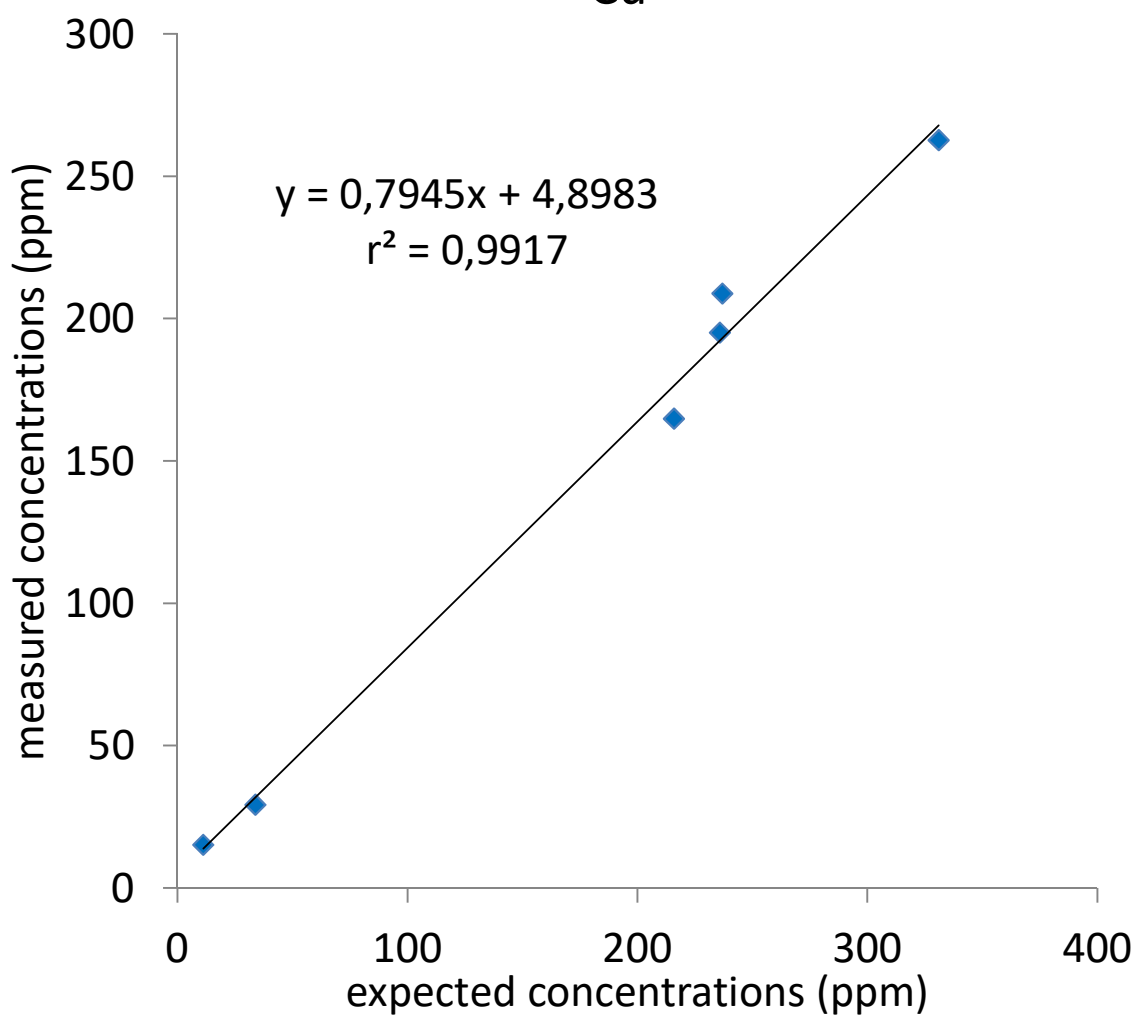

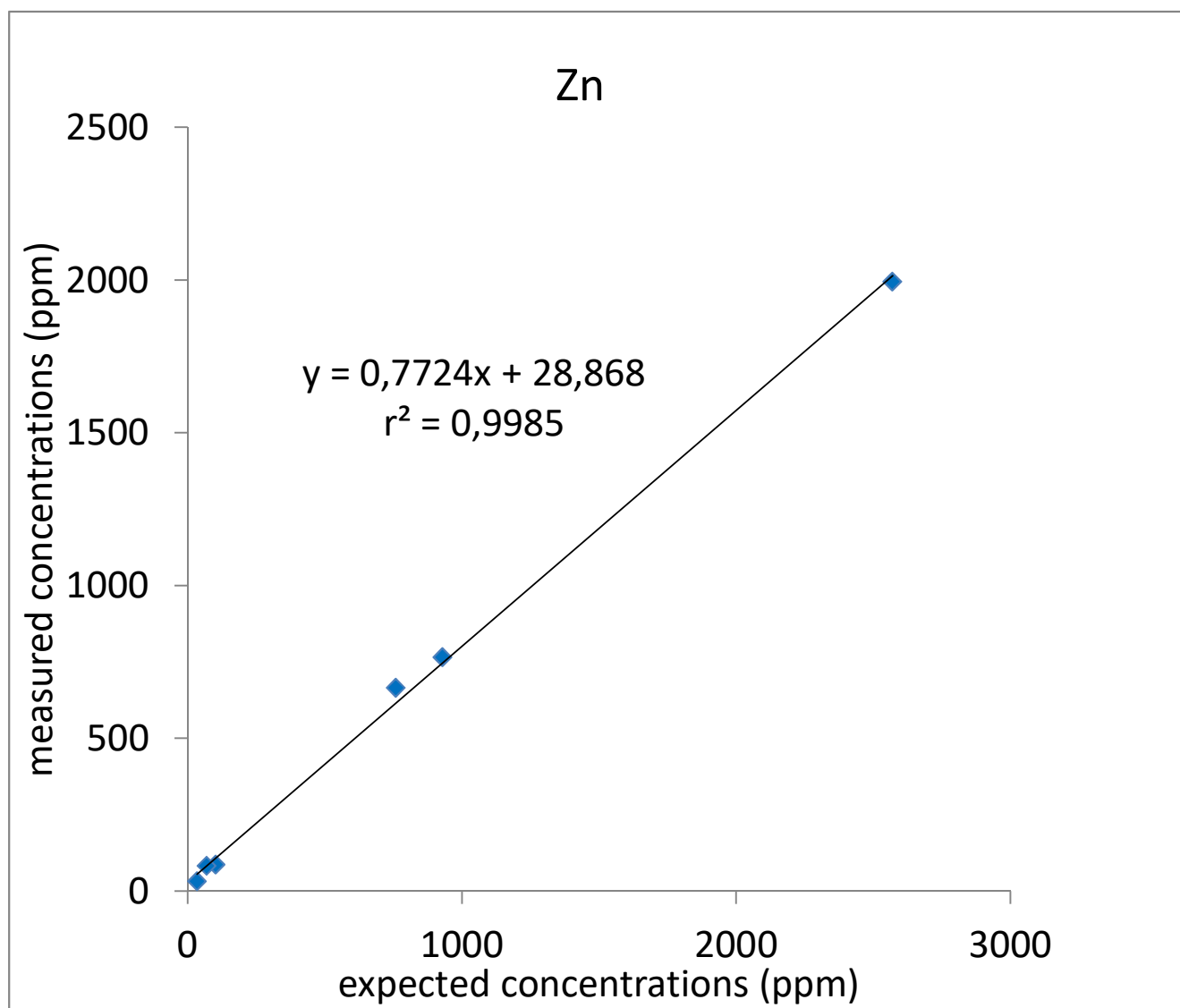

Supplement: S1 Fig — (PDF) [file pone.0214218.s004.pdf]
