# Supplementary material for: The earliest lead ore processing in Europe. 5th millennium BC finds from Pietrele on the Lower Danube
Source: PLoS One. 2019 Apr 10;14(4):e0214218. doi: 10.1371/journal.pone.0214218 (PMC6457500; doi:10.1371/journal.pone.0214218)
Supplement: S1 Supporting Information — (DOCX) [file pone.0214218.s005.docx]

S1 Supporting Information. Portable-XRF – methods and limitations

The decision to use a p-XRF device, among other reasons, was based on keeping the samples and ‘crucibles’ intact. There is also a responsibility for archaeological finds in general, as they are artefacts of cultural heritage and are extremely scarce. As there are several well-known and severe problems presented when using the p-XRF device, especially when analysing prehistoric ceramic and slag-like material without first homogenizing and pulverizing it, a short discussion of the limitations and possibilities of the methodology is required. Although the p-XRF is often referred to provide only qualitative data, a comparison with results from SEM indicates that the p-XRF yields qualitative measurement results with a certain, but often minimal bias, that can be used to sort the samples according to higher or lower amounts of metal traces.

Device settings and procedure

It is outside the framework of this appendix to delve into excessive details, so we believe only a brief review of the most important factors enabling us to get an idea of the reliability of the p-XRF measurements on the finds in focus is sufficient. A great variety of topics on p-XRF analysis has been published concerning the sample preparation, device settings, the environmental conditions during the measurements, the data processing and calibration, down to preliminary or final interpretation of the results. These are discussed in detail in a multitude of excellent papers on which we rely and to which we refer. For pottery and geological/ore samples, see: Böhme – Helfert 2010; Helfert 2010, 2013; Behrendt et al. 2012; Hunt – Speckman 2015; Hall et al. 2012, 2014; Gallhofer – Lottermoser 2018; Speckman et al. 2011; Piercey – Devine 2014; Kenna et al. 2011; Rouillon – Taylor 2016; Shugar 2013; Shugar – Mass 2012; Speakman – Shackley 2013; Brand – Brand 2014. The p-XRF analysis of metal artefacts is recently discussed in: Cesareo et al. 2008; Lutz – Pernicka 1996; Pernicka 2014; Potts – West 2008; Prange et al. 2016; Shugar 2013; Shugar – Mass 2012) and for slags and crucibles in: (Bandama et al. 2016; Dungworth 2015; Orfanou – Rehren 2015; Rademakers – Rehren 2016; Scott et al. 2015; Slater – Doonan 2012; Tighe et al. 2018; Young et al. 2016; Zori – Tropper 2013.

For the measurements, a portable ED-XRF device was used (Thermo Scientific Niton XL3t 950-HE GOLDD+ Serial nr. 89086), equipped with an Ag anode at max. 50 kV, silicon drift detector. A series of four filters were applied (main, low, high and light) by running the so-called test-all-geo mode, which is a combination of both the mineral- and the soil-modes. The filter settings were 90/90/60/120 seconds, resulting in a total measuring time of 6 minutes. The aperture has a diameter of 8 mm and neither helium nor vacuum was applied. In order to keep the window clean, the measurements were made with a 4 micrometer polypropylene foil (Fluxana, TF-240-255) between the p-XRF window and the sample. All measurements were performed in a benchtop stand using AC adapter. These settings were used for the measurements of certified reference material (CRM) and the samples from Pietrele and Blejeşti presented in this work. They follow the recommended procedure for the device, established during several years of evaluating and optimizing ceramic analyses with the Niton XL3t, by the Forschungsstelle Keramik at the Institut für Archäologische Wissenschaften, Goethe-Universität (Frankfurt a. M.) in cooperation with Analyticon Instruments (Rosbach v. d. Höhe), the company responsible for the service of Niton devices in Germany (Böhme – Helfert 2010; Helfert 2010, 2013). All Niton XL3t devices purchased by Analyticon Germany for archaeological use underwent a specialised calibration procedure for archaeometric purposes by the technical support of this company, which adjusts the mineral mode and the general and precious metals mode for archaeological material and artefacts. The result is a calibration process better adapted to archaeological needs compared to factory calibration. Unfortunately, few details of the exact procedure and used samples are available since it is proprietary software – a problem known to all p-XRF users, no matter what device or brand (Hunt – Speckman 2015; Hall et al. 2012; 2014).

General precaution

The device was allowed to warm up for ca. one hour before measuring blanks CRM and samples, as temperature drift can have effects on the results in the form of so-called noise. For the Niton XL3t, such drift disappears after the machine has been running for a while (Böhme – Helfert 2010; Helfert 2013; Hall et al. 2014; Gallhofer – Lottermoser 2018; Speakman – Shackley 2013).

Air humidity and air temperature should be kept at a normal indoor climate, as the water molecules in the air between the analyzer and the sample have severe effects on the measurements and high ambient temperature can cause overheating of the device, resulting in a shutdown. Both are monitored continuously. Also, a low water content of the sample is important (Helfert 2013; Shugar 2013). As with all studied samples from excavations years or decades ago, they were stored under controlled humidity conditions for a significant period of time, so we can expect them to be dry.

Problems and limitations

The measurements had to be performed on the surfaces of the samples, which were either freshly cut, freshly broken, results of post-excavation breaks or old surfaces. To get optimal results, the samples should be homogenized by grinding them into powder. But, as mentioned above, that was not an option for this study. A slight variation between the results can therefore be expected, due to contamination from deposition and storage.

To get an idea of the variability of the chemical composition related to uneven concentrations, each sample was measured at least three times on different spots. They give a mean concentration for the samples surface in an area of 8 mm in diameter (aperture of the used device). Also, it is clear that inhomogeneity could be tracked down to the sub-microscopic scale, but the question remains as to whether or not this is necessary in this case. As our focus is on the general chemical composition of the samples and the detection of peculiar lead traces, the sum of each of the 8 mm in diameter surface measurements is sufficient.

It is known that a samples surface geometry influences the refraction of radiation. Another source of distortion is related to the uneven distribution of e.g. mineral temper in a clay matrix and chemical inhomogeneity in general. Crystallized components and grain size strongly influence the path of X-rays and, especially in combination with amorphous material, they amplify distortions in the readout of the detected fluorescence energies and their interpretation in the spectra. As radiation cannot penetrate materials of varying density and crystal structure in the same way, denser particles in the surface layer may prevent deeper-lying lighter components from being activated and detected. Thus, influences on the count rate of the detector lead to false calculations of the sample’s composition. These interactions are summed up to some extent in the term “matrix effect” and are further reasons why ground and homogenized samples are advantageous (Hunt – Speckman 2015; Hall et al. 2012, 2014; Gallhofer – Lottermoser 2018; Piercey – Devine 2014; Kenna et al. 2011; Shugar 2013).

Even if ideal fine-grained and homogenous samples are analysed, a multitude of complex phenomena can arise when interpreting the fluorescence spectra in order to determine the presence of a certain element and its quantity by plotting the count rate of detected radiation versus its energy. The final result is usually generated by proprietary software preloaded in the system – and herein lies its sensitive spot. To calculate the quantity of an element, the identification of its main lines (e.g. Kα and Kβ) and their peak’s height relation to each other are necessary data. As the peaks of several elements differ only slightly in their energy, they can overlap to some extent with a peak of another element. Arsenic and lead are examples of this. The result is a sum peak with count rates too high for an element while the other element may not be reported by the software at all. Besides radiation effects in the sample itself (e.g. Bremsstrahlung), so-called escape peaks in the detector can produce radiations that are mere interferences inherent to the system, but they can contribute severely to the count rate of an element (Hunt – Speckman 2015; Hall et al. 2012, 2014; Gallhofer – Lottermoser 2018; Piercey – Devine 2014; Kenna et al. 2011; Shugar – Mass 2012; Speckman et al. 2011).

Because of spectral interference, many elements are only adequately detectable in real world samples when present in amounts considerably higher than their limit of quantification (LOQ) would be when measured in an ideally clean matrix, i.e. in combination with elements that do not interfere with each other. The limit of detection (LOD) is defined as the lowest concentration for which the mere presence of an element can be detected and the LOD is often given for elements in SiO_2_ matrix by the device’s provider. This avoids many interference sources present in most rocks or artefacts and suggests a very low LOD instead. A LOD should not be confused with the LOQ, the lowest of an element’s quantifiable concentration, which is by definition 3.3 times higher than the LOD and is the truly relevant parameter (Thomsen et al. 2003).

All these spectra related topics are well-studied but too complex for automatic software to be solved by computing algorithms alone. The human researcher has to control and decide here, as is the usual procedure for many laboratory-based analytics. It is outside the objective of this paper to discuss these subjects thoroughly. They are described in depth in several brilliant studies (Hunt – Speckman 2015; Hall et al. 2012, 2014; Gallhofer – Lottermoser 2018; Conrey et al. 2013; Kenna et al. 2011).

Evaluation of data quality

The usual procedure to overcome some of the mentioned severe problems is the so-called “matrix matched calibration”. By measuring samples of known composition (determined by other methods) with roughly the same element range that consists of the same material, plus ideally having comparable surfaces as the objects of study, the deviation of the p-XRF measurement in relation to the expected value is used for calibration.

For archaeological ceramics and potter’s clay, a SiO_2_-rich matrix is generally assumed as sufficient for calibration. Some teams use powdered obsidian as reference material because its components are homogenously distributed and the material is predominantly amorphous (Speckman et al. 2011). Nevertheless, calcium- or alumina-rich pottery has to be kept in mind when e.g. lime-rich temper (coarse or fine-ground) was added or clay mineral’s concentration increased by intense levigation.

One aim of this paper is to discuss if a study using p-XRF of the material under investigation turns out to be sufficient to pose several questions, e.g. if traces of lead or metal in general are present on or in some ceramic vessels or not. According to the aforementioned difficulties and limitations of the p-XRF method, the accuracy of the measuremts of the device used must be evaluated first. As stated above, an ideal matrix matched calibration cannot be applied at the current state of the project. To illustrate the accuracy and comparability of the results given by the p-XRF device used, we present and discuss the evaluation of 497 measurements made between 2015 and 2018 on several commonly used CRM composed of different geological and industrial material with different elemental concentrations (S1 and S2 Tables). According to geochemical standard procedure, the results of the main components (except Na) are calculated as oxides and normalised to 100 wt%. Besides lead, this study focused only on the traces of zinc, copper and sulfur. The possibilities and difficulties of their detection in ceramics with p-XRF is discussed in literature also to some extent in the following papers: Hunt – Speckman 2015; Hall et al. 2012, 2014; Gallhofer – Lottermoser 2018; Conrey et al. 2013; Bandama et al. 2016; Dungworth 2015; Orfanou – Rehren 2015; Rademakers – Rehren 2016; Scott et al. 2015; Slater – Doonan 2012; Tighe et al. 2018; Young et al. 2016; Zori – Tropper 2013.

Although the CRM concentrations of several elements in question do not cover the complete range necessary to establish ideal calibration factors for pottery, the dataset is useful to get an idea of the effective magnitudes. This can serve as a practical estimation of the reliability and comparability of the results and is expressed in several parameters. The expected values given for the standards are compared to the mean value of the p-XRF measurements, the standard deviation (SD) of the p-XRF result is given as well as the relative standard deviation (RSD) or precision (S2 Table). Furthermore, the regression analysis and graphical comparison against reference values is used to assess the accuracy of p-XRF measurements, i.e. the so-called ‘goodness of fit’ or comparability, expressed by the linear coefficient of correlation (r²) and the graph parameters intercept and slope (S3 Table and S1 Figure). (Rawski et al. 2016; Rouillon – Taylor 2016; Ramsey – Ellison 2007; Hunt – Speckman 2015; Hall et al. 2012, 2014; Gallhofer – Lottermoser 2018; Piercey – Devine 2014; Kenna et al. 2011).

We follow the criteria for characterising data quality proposed by Rouillon & Taylor 2016, respectively US EPA 1998, for defining three data quality levels, supplemented by a review of relative bias following Rostron & Ramsay 2017. For the highest level known as the ‘definitive level’, the statistical parameters are r² = 0.85 – 1 and RSD ≤ 10 %. The medium level, named ‘quantitative screening’, has r² = 0.7 – 1.0 and RSD < 20 % and the lowest level, called ‘qualitative screening’, with r² < 0.7 and RSD > 20 %. In other words, level one means that two datasets (e.g. the given standard and actual measurement) are statistically similar (at a level of 5 %), level two means that there is a linear correlation between them (i.e. their deviation) that can be corrected (by calibration) and level three means that they are statistically different datasets (no linear relation means no calibration is possible).

The performance of the Niton XL3t for seven of nine main oxides is good, as eight have relative standard deviations (precision) less than 10% for all analysed CRM and also seven have a coefficient of correlation higher than 0.85. Thus, the device gives accurate and precise data for SiO_2_, TiO_2,_ Al_2_O_3_, Fe_2_O_3(Total)_, MnO, CaO and K_2_O. The RSD for MgO is greater than 20% for most CRM and the correlation of P_2_O_5_ far below 0.7 which means both fall out of the quantitative screening level and within the level of qualitative screening only, calibration by the aid of the given CRMs is not possible. This result is not surprising, as both are reported frequently as problematic for p-XRF, especially when they appear in low concentrations (Hunt – Speckman 2015; Hall et al. 2012, 2014; Gallhofer – Lottermoser 2018).

In addition to the oxidic main components, we took a closer look also to a few selected trace elements, namely lead, zinc, copper and sulfur. According to their RSD and r², the device gives data for three elements within the definitive quality level. Only copper has in two cases (Loess 1 and NIST 2780) a RSD higher than 20% while it remains below 10% for the other standards. Its coefficient of correlation is excellent, with 0.99. Although this outcome is promising and consistent with published results of many teams evaluating different portable and benchtop XRF devices in archaeology and geology, also concerning Mg and P (Hunt – Speckman 2015; Hall et al. 2012, 2014; Gallhofer – Lottermoser 2018), it is appropriate to have a look at the magnitude of bias between the expected and measured values of the Niton XL3t (S2 Table).

In sum, the elements Pb, Cu, Zn and S and most oxides (with the exception of MgO and P_2_O_5_) give datasets that are in linear correlation to the standards while having little variation and low noise. Stronger discrepancy is noticeable when monitoring bias and relative bias, the latter should ideally be less than 10% (Rostron & Ramsay 2017). None of the oxides can catch up completely with this requirement because for several CRM the relative bias exceeds 10% considerably while the value is satisfying for other standards. For SiO_2_, relative bias between ca. 1 and 16% is reported (0% for the blank check sample of pure SiO_2_). Between ca. 6 and 15% for TiO_2_, ca. 2 – 23% for Al_2_O_3_, between ca. 5 and 32% for Fe_2_O_3(T)_, ca. 8 – 25% for MnO, ca. 4 to up to 54% for CaO and ca. 6 – 44% for K_2_O. For Cu, Zn and S also high relative bias is attested while only Pb remains below ca.10% for all except one CRM (NIST 2780, which has also the highest rel. bias for Cu, Zn and S).

These are high bias values, but the bias is precisely what will be adjusted much more towards the correct value by calibration. At the moment we will keep the data as they are to discuss several other issues not often referred to in literature.

Especially the CRM Loess-1, but also Till-4 and others to some extent, show high relative bias which can relate partially to a high content of elements not measurable with the p-XRF, i.e. Na_2_O and a sum of light volatile components combined under the term loss on ignition (LOI, cf. S1 Table). Alongside H_2_O and OH, larger amounts of CO_2_ bound in calcium carbonate (calcite, a main component in marls and limestones) have strong effects on the final result of the calculation and normalisation of the main oxidic components to a sum of 100 wt%; in CaCO_3_, considerably less Ca is bound than in CaO (factor c. 0.56). By calculating the complete Ca of the Loess-1 measurements as carbonate (by relying also on the so-called ‘balance’, a value that is calculated by the devices’ software that should express the amount of non-measurable components), one can come up with an amount of CO_2_ around 14.4 wt%, given by the standard 14.9 wt%. Recalculating CaO then leads to 18.3 wt%, which is not ideal but quite a bit closer to the expected 16.3 wt% and considerably better than the former 25.2 wt% obtained by previous normalisation. If this procedure is accepted, then the recalculation of the remaining oxides results in a better fit to their specified values also. The limitations of this paper prevent us from following that hypothesis thoroughly and this procedure does not work as effectively as in this example for all CRM we investigated, where surely also other components and effects complicate the matter. It nevertheless highlights an important topic for both the analysis of ceramic and clay. As soon as samples contain higher amounts of carbonates, resulting from e.g. a lime or marl rich clay or from adding temper, the increased amount of carbonate not detectable for any non-invasive surface method will create considerable problems because it is overlooked in the normalisation (Hunt – Speckman 2015; Hall et al. 2012, 2014; Conrey et al. 2013). Only invasive analytical methods can give proper estimations for the amount of LOI (e.g. by heating powdered samples in an oven and monitoring its weight loss), but also the detection of calcite or marl in thin section microscopy can help with estimating the presence of carbonates in the samples and give indications to help fit the normalisation on calcium carbonate instead of calcium oxide.

To conclude, it is clear that the Niton XL3t can provide precise and comparable data – to some extent – for most main and selected trace elements with the exception of Mg and P. As the difficulties related to these two components are well known and widespread, at least for samples with low content and without adjusted calibration, Mg and P have to be omitted in studies of elemental composition or they have to be compared to results from other analytical methods. The remaining elements have a high potential for archaeological and geochemical interpretation, but as long as no matrix matched calibration is performed, the relative bias described above remains operative for all components and has to be taken into account when talking about the concentration of an element or an oxide. The strong influence of LOI and carbonates remains a serious factor. As described above, we reject the calibration with the set of CRM used at the present stage of research since we prefer to discuss the p-XRF results in contrast to analysis with other methods (SEM, XRD and optical microscopy) on original material of the study.

References

Bandama F, Moffett AJ, Thondhlana TP, Chirikure S. The Production, Distribution and Consumption of Metals and Alloys at Great Zimbabwe. Archaeometry 58, 2016, Issue S1, 164-181.

Behrendt S, Mielke DP, Mecking O. Die portable Röntgenfluoreszenzanalyse (p-RFA) in der Keramikforschung: Grundlagen und Potenzial, Restaurierung und Archäologie 5, 2012, 93 –110.

Böhme D, Helfert M. Herkunftsbestimmung von römischer Keramik mittels portabler energiedispersiver Röntgenfluoreszenzanalyse (P-ED-RFA) – Erste Ergebnisse einer anwendungsbezogenen Teststudie, in: B. Ramminger – O. Stilborg (Hrsg.), Naturwissenschaftliche Analysen vor- und frühgeschichtlicher Keramik I. Methoden, Anwendungsbereiche, Auswertungsmöglichkeiten. Erster u. zweiter internationaler Workshop für junge Wissenschaftlerinnen u. Wissenschaftler in Hamburg am 9. Februar 2008 u. 7. Februar 2009, UPA 176 (Bonn 2010) 11–30.

Brand NW, Brand CJ. Performance comparison of portable XRF instruments: a mineral exploration industry prospective. Advances in X-ray Analysis, 2014 Volume 57, 236-262.

CanmetMINING. Certified Reference Material: Geochemical Soil and Till Reference Materials. Version: November 1995, Ottawa.

Cesareo R, Ridolfi S, Marabelli M, Castellano A, Buccolieri G, Donativi M, et al. Portable systems for energy-dispersive X-ray fluorescence analysis of works of art. In: Potts PJ, West M, editors. Portable X-ray Fluorescence Spectrometry - Capabilities for In Situ Analysis. Cambridge: Royal Society of Chemistry; 2008; pp. 206-246.

Conrey RM, Goodman-Elgar M, Bettencourt N, Seyfarth A, Van Hoose A, Wolff JA. Portable X-ray fluorescence spectrometer calibration for archaeological. Geochemistry: Exploration, Environment, Analysis. <http://dx.doi.org/10.1144/geochem2013-198> Published Online First.

Dungworth D. Archaeometallurgy: Guidelines for Best Practice. Historic England, 2015.

Frahm E. Validity of “Off-the-Shelf” Handheld Portable XRF for Sourcing Near Eastern Obsidian Chip Debris. Journal of Archaeological Science 40(2) 2013:1080-1092.

Gallhofer D, Lottermoser BG. The Influence of Spectral Interferences on Critical Element Determination with Portable X-Ray Fluorescence (pXRF). Minerals 2018, 8, 320; doi:10.3390/min8080320 [www.mdpi.com/journal/minerals](http://www.mdpi.com/journal/minerals).

Hall G, Buchar A, Bonham-Carter G. Quality Control Assessment of Portable XRF Analysers: Development of Standard Operating Procedures, Performance on Variable Media and Recommended Uses. CAMIRO Project 10E01, Phase 1, 2012 <http://www.appliedgeochemists.org>.

Hall GEM, Bonham-Carter GF, Buchar A. Evaluation of portable X-ray fluorescence (pXRF) in exploration and mining: Phase 1, control reference materials. Geochemistry: Exploration, Environment, Analysis, Vol. 14, 2014, pp. 99–123.

Helfert M. From a Pile of Potsherds to the Data Mountain – Archaeological and Geochemical Studies on Ceramic Production in the Roman Kastellvicus of Groß-Gerau "Auf Esch", in: B. Ramminger – O. Stilborg (Hrsg.), Naturwissenschaftliche Analysen vor- und frühgeschichtlicher Keramik I. Methoden, Anwendungsbereiche, Auswertungsmöglichkeiten. Erster u. zweiter internationaler Workshop für junge Wissenschaftlerinnen u. Wissenschaftler in Hamburg am 9. Februar 2008 u. 7. Februar 2009, UPA 176 (Bonn 2010) 123–135.

Helfert M. Die portable energiedispersive Röntgenfluoreszenzanalyse (P-EDRFA) – Studie zu methodischen und analytischen Grundlagen ihrer Anwendung in der archäologischen Keramikforschung, in: B. Ramminger – O. Stilborg, M. Helfert (Hrsg.), Naturwissenschaftliche Analysen vor- und frühgeschichtlicher Keramik III. Methoden, Anwendungsbereiche, Auswertungsmöglichkeiten, UPA 238 (Bonn 2013) 15–47.

Hunt AMW, Speakman RJ. Portable XRF analysis of archaeological sediments and ceramics. Journal of Archaeological Science 53 (2015) 1-13.

Kenna TC, Nitsche FO, Herron MM,Mailloux BJ, Peteet D, Sritrairat S, Sands E, Baumgarten J. Evaluation and calibration of a Field Portable X-Ray Fluorescence spectrometer for quantitative analysis of siliciclastic soils and sediments. J. Anal. At. Spectrom., 2011, 26, 395–405.

Lutz J, Pernicka E. Energy dispersive X-ray fluorescence analysis of ancient copper alloys: empirical values for precision and accuracy. Archaeometry. 1996;38,2: 313-323.

Orfanou V, Rehren T. A (not so) dangerous method: pXRF vs. EPMA-WDS analyses of copper-based artefacts. Archaeol. Anthropol. Sci. (2015) 7: 387–397.

Pernicka E. Provenance Determination of Archaeological Metal Objects. In: Roberts BW, Thornton CP, editors. Archaeometallurgy in Global Perspective. New York: Springer; 2014: pp. 239-268.

Piercey SJ, Devine MC. Analysis of powdered reference materials and known samples with a benchtop, field portable x-ray fluorescence (pXRF) spectrometer: evaluation of performance and potential applications for exploration lithogeochemistry. Geochemistry: Exploration, Environment, Analysis <http://dx.doi.org/10.1144/geochem2013-199> Published Online First 2014.

Potts P. Reference Material Data Sheet, SdAR-M2 Metal-rich sediment, International Association of Geoanalysts. 2015.

Potts PJ, Thompson M, Chenery SRN, Webb PC, Kasper HU. GEOPT13 – an international proficiency test for analytical geochemistry laboratories – report round 13 / July 2003 (Köln Loess).

Potts PJ, West M, editors. Portable X-ray Fluorescence Spectrometry - Capabilities for In Situ Analysis. Cambridge: Royal Society of Chemistry; 2008.

Prange M, Modarressi-Tehrani D, Demant D. Some Thoughts on the Benefits and Challenges of Working with pXRF. In: Körlin G, Prange M, Stöllner T, Yalçın Ü, editors. From bright ores to shiny metals – Festschrift for Andreas Hauptmann. Rahden/Westfahlen: Leidorf; 2016: pp. 247-256.

Rademakers FW, Rehren T. Seeing the forest for the trees: Assessing technological variability in ancient metallurgical crucible assemblages. Journal of Archaeological Science: Reports 7 (2016) 588–596.

Ramsey MH, Ellison SLR. Measurement uncertainty arising from sampling: A guide to methods and approaches. Eurachem. University of Sussex, UK, 2007.

Rawski RI, Sanecki PT, Kijowska KM, Skital PM, Saletnik DE. Regression Analysis in Analytical Chemistry. Determination and Validation of Linear and Quadratic Regression Dependencies. S. Afr. J. Chem., 2016, 69, 166–173, <http://journals.sabinet.co.za/sajchem/>.

Rostron PD, Ramsey MH. Quantifying heterogeneity of small test portion masses of geological reference materials by PXRF: implications for uncertainty of reference values. Geostandards and Geoanalytical Research, 2017, Vol. 41, Iss. 3, 459-473.

Rouillon M, Taylor MP. Can field portable X-ray fluorescence (pXRF) produce high quality data for application in environmental contamination research? Environmental Pollution 214 (2016) 255-264.

Scott RB, Eekelers K, Fredericks L, Degryse P. A methodology for qualitative archaeometallurgical fieldwork using a handheld X-ray fluorescence spectrometer, STAR: Science & Technology of Archaeological Research, (2015) 1:2, 70-80.

Shugar, AN. Portable X-ray fluorescence and archaeology: limitations of the instrument and suggested methods to achieve desired results. Archaeological chemistry VIII, vol 1147, American Chemical Society Washington DC (2013) 173-193.

Shugar AN, Mass JL, editors. Handheld XRF for art and archaeology. Studies in Archaeological Sciences 3. Leuven University Press (2012).

Slater JL, Doonan RCP. Envaluing past practice: a framework for the spatial analysis of metal production in first millennium BC Britain.Proceedings of the 39th International Symposium for Archaeometry, Leuven (2012) 113-119.

Speakman RJ, Shackley MS. Silo science and portable XRF in archaeology: a response to Frahm. Journal of Archaeological Science 40 (2013) 1435-1443.

Speakman RJ, Little NC, Creel D, Miller MR, Iñañez JG. Sourcing ceramics with portable XRF spectrometers? A comparison with INAA using Mimbres pottery from the American Southwest. Journal of Archaeological Science 38 (2011) 3483e3496.

Thermo Scientific. Certificate of Analysis, Thermo Scientific Niton XRF Analyzers. Part Number 410-014b, [www.thermoscientific.com/niton](http://www.thermoscientific.com/niton), Thermo Scientific Inc. Boston, 2013.

Thomsen V, Schatzlein S, Mercuro D. Limits of Detection in Spectroscopy. Spectroscopy 18 (12), December 2003, 112-114.

Tighe M, Rogan G, Wilson SC, Grave P, Kealhofer L, Yukongdi P. The potential for portable X-ray fluorescence determination of soil copper at ancient metallurgy sites, and considerations beyond measurements of total concentrations. Journal of Environmental Management 206 (2018) 373-382.

US EPA. United States Environmental Protection Agency (US EPA), 2004. Method 6200-Field Portable X-ray Fluorescence Spectrometry for the Determination of Elemental Concentrations in Soil and Sediment, pp. 1e32 (accessed 27 08 15). http:// www3.epa.gov/epawaste/hazard/testmethods/sw846/pdfs/6200.pdf.

Webb PC, Thompson M, Potts PJ, Wilson SA. GeoPT36A – an international proficiency test for analytical geochemistry laboratories – report on round 36A (Metal-rich sediment, SdAR-M2) / January 2015.

Wise SA, Robert L. Watters RLJr. National Institute of Standards & Technology, Certificate of Analysis, Standard Reference Material 2709a, San Joaquin Soil, Baseline Trace Element Concentrations, 2009.

Wise SA, Robert L. Watters RLJr. National Institute of Standards & Technology, Certificate of Analysis, Standard Reference Material 2780, Hard Rock Mine Waste, 2012.

Young KE, Evans CA, Hodges KV, Bleacher JE, Graff TG. A review of the handheld X-ray fluorescence spectrometer as a tool for field geologic investigations on Earth and in planetary surface exploration. Applied Geochemistry 72 (2016) 77-87.

Zori C, Tropper P. Silver lining: evidence for Inka silver refining in northern Chile. Journal of Archaeological Science (2013) 40, Issue 8. 3282-3292.
